# Formation of Gallium Monofluoride in the Coordination Sphere of Nickel

**DOI:** 10.1002/anie.1189853

**Published:** 2026-06-18

**Authors:** Johannes Stephan, Samia Kahlal, Jean‐Yves Saillard, Marvin Mühlau, Lisa Gremmel, Christian Gemel, Roland A. Fischer

**Affiliations:** ^1^ TUM School of Natural Sciences, Department of Chemistry, Chair of Inorganic and Metalorganic Chemistry Technical University of Munich Garching Germany; ^2^ Catalysis Research Center Technical University of Munich Garching Germany; ^3^ Univ Rennes CNRS, ISCR‐UMR 6226 Rennes France; ^4^ TUM School of Natural Sciences, Department of Chemistry TUMint.Energy Research GmbH Technical University of Munich Garching Germany

**Keywords:** carbene homologues, density functional calculations, fluorides, gallium, heterometallic complexes

## Abstract

Monovalent gallium halides are intrinsically unstable compounds at room temperature and can only be generated in the gas phase at temperatures of about 1000 °C. Although they are isoelectronic to carbon monoxide, dinitrogen, and the cyanide ion, which are pivotal ligands in coordination chemistry, monovalent gallium halides are largely unexplored as ligands with transition metals. Herein, we report the isolation of a dicationic nickel complex bearing a gallium monofluoride ligand. The +I oxidation state of the gallium centre is supported by NMR‐spectroscopic methods, X‐ray photoelectron spectroscopy, as well as density functional theory calculations. Analysis of the bonding situation between the GaF ligand and the central nickel atom suggests strong σ‐donating, but negligible π‐accepting properties for the supported GaF fragment. The GaF moiety forms as a product of C(sp^3^)–F bond activation of the weakly coordinating BAr^F24^ (tetrakis[(bis‐3,5‐trifluoromethyl)phenyl]borate) anion and serves as a fluorine source for silicon and carbon electrophiles. This leads to facile defluorination, forming the fluoride‐free complex and fluorinated products such as fluorotrimethylsilane or benzoyl fluoride.

## Graphical abstract

The elusive gas‐phase species gallium monofluoride forms selectively in the coordination sphere of a nickel(II) centre as a product of C(sp^3^)–F bond activation, with a weakly coordinating anion as the fluorine source. The herein reported gallium monofluoride ligand acts as a very strong σ‐donor ligand at nickel and serves as a fluorine donor towards silicon and carbon electrophiles.

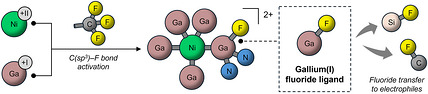

## 1 Introduction

Carbon monoxide has been the cornerstone of organometallic chemistry ever since Ni(CO)_4_ was isolated as the first transition metal carbonyl complex [1]. Other diatomic molecules, like dinitrogen or the cyanide anion (CN^–^) are also known to bind well to transition metals, following the isolobal analogy [2, 3, 4, 5]. While CO, N_2_, and CN^–^ are stable molecules at room temperature, their isoelectronic counterparts in group 13, the monovalent halides of boron, aluminium, or gallium, exhibit markedly different physicochemical properties. Although formally isolobal to CO, these species are notoriously unstable and cannot be isolated in pure form at room temperature. Group 13 monohalides can be generated only under extreme conditions, typically in the gas phase at temperatures of about 1000 °C. Reported synthetic methods involve comproportionating reactions that combine the group 13 element E with its trihalide EX_3_ (X = F, Cl, Br, I) [6], as well as reactions of the metals with hydrogen halides [7], or halogens [8]. In addition, the reaction of solid reagents, like CaF_2_ [9] or AlF_3_ [10] and gallium or aluminium metal can be harnessed to access the corresponding monofluorides [11, 12]. Regardless of the method, these compounds rapidly disproportionate at ambient conditions to give the elemental metal and the corresponding trihalide, both of which are thermodynamically favoured [13]. In general, monofluorides are the least stable members of this exotic class of compounds, with stability increasing with the size of the halide [14]. Despite their transient nature, monovalent group 13 halides are not merely chemical curiosities, as they have demonstrated in particular the ability to activate small organic molecules, such as acetylene, in gas phase reactions [15].

Their intrinsic instability, however, has made synthetic access to these species particularly challenging. Consequently, working with monovalent group 13 halides requires sophisticated experimental setups, usually involving high‐temperature syntheses combined with rapid cooling techniques. These are typically stabilised by adding donor ligands, such as ethers or amines, to produce metastable solutions. As a result, only a limited number of stable examples have been reported to date. The best‐known are donor‐stabilised cluster compounds, such as Al_4_Br_4_ [16], Al_4_I_4_ [17], Ga_8_Br_8_[18], and Ga_8_I_8_[19] reported by the Schnöckel group. In solution, AlCl [20] and GaCl [21] have been employed as precursor materials for the synthesis of monovalent group 13 organometallics, such as AlCp* [22], GaCp [23], and GaCp* [23], or larger metalloid clusters of aluminium [24, 25] and gallium [26, 27].

**FIGURE 1:**
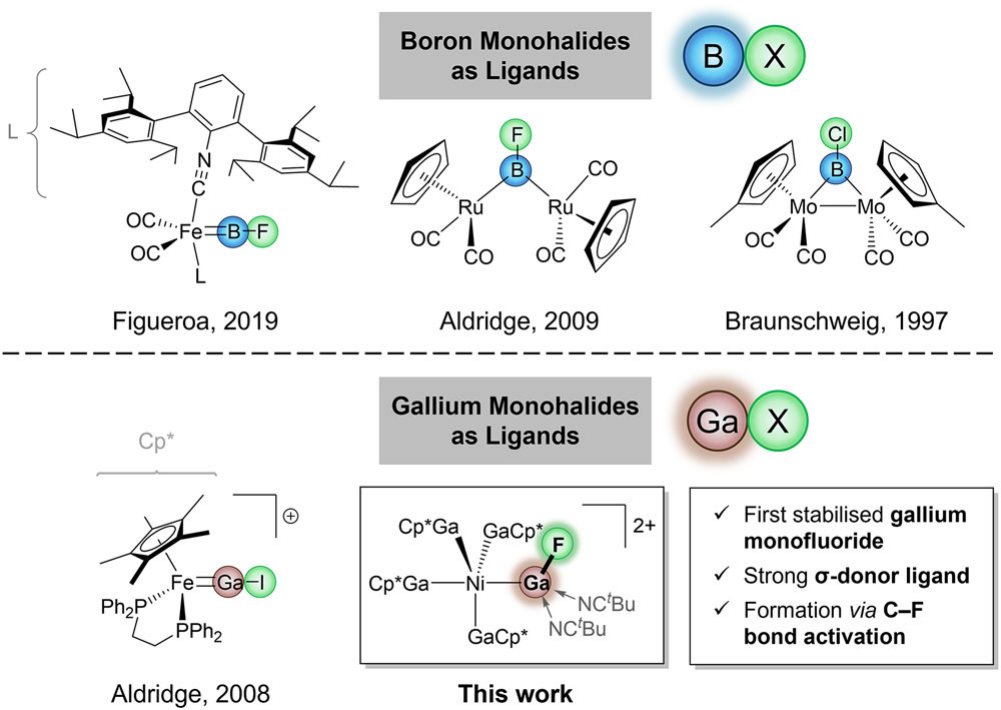
Overview of group 13 monohalides as ligands to transition metal centers. Two coordination modes (terminal and bridging) have been reported in the literature to date. Whereas two examples of boron monofluoride complexes exist, a gallium monofluoride is unprecedented.

Given their challenging synthesis, it is not surprising that the coordination chemistry of subvalent group 13 halides remains largely unexplored when compared with that of CO, N_2,_ or CN^–^. To date, only four well‐established examples are known, in which such fragments are trapped by coordination to a transition metal: boron monofluoride (BF) [28] and gallium monoiodide (GaI) [29, 30] as terminal ligands to iron, a BF bridging two ruthenium centres [31], and a BCl fragment bridging two molybdenum centres [32] (Figure 1). However, these complexes are not accessible directly from the corresponding group 13 monohalides. Instead, wet‐chemical approaches, such as salt metathesis or halide abstraction, have been utilised for their synthesis [28, 29, 30, 31, 32].

In this contribution, we report a rare example of a room‐temperature‐stable gallium(I) fluoride fragment, stabilised by nitrile ligands, that coordinates to a Ni(II) centre. This species forms selectively through C(sp^3^)–F bond activation of a fluorinated weakly coordinating anion. To our knowledge, this represents the first structurally characterised instance of a “true” gallium(I) fluoride moiety acting as a ligand at a transition metal centre. DFT calculations support the retention of the +I oxidation state at gallium and reveal that the Ga–F unit is a significantly stronger σ‐donor than GaCp*. Upon exposure to silicon or carbon electrophiles, the fluorine atom of the gallium fluoride moiety is readily transferred at room temperature, furnishing the fluoride‐free complex and fluorinated (organic) products.

## 2 Results and Discussion

This study builds upon our long‐standing interest in mixed transition‐metal/main‐group‐metal complexes and clusters that feature polarised metal‐metal bonds. In particular, we have demonstrated that incorporating cationic metal centres enhances bond polarisation, thereby increasing the Lewis acidity of the main‐group metal and promoting bond‐activation processes. Such reactivity has been exemplified by transformations such as the dimerisation of acetonitrile by a heterometallic nickel‐gallium complex based on a formal Ni(II) centre, which is indeed driven mainly by Lewis acidity [33].

### 2.1 Synthesis of [(Cp*Ga)_4_Ni(GaF(NC*^t^* Bu)_2_)](BAr^F24^)_2_ (**1**)

**FIGURE 2:**
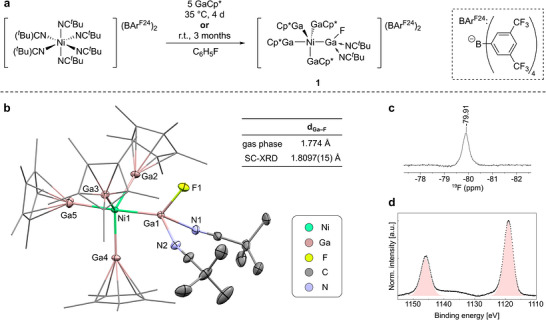
(a) Synthesis of **1** *via* C–F bond activation. (b) Molecular structure of the dicationic part of **1** as determined by SC‐XRD. Ellipsoids are drawn at 50% probability; Cp* ligands are drawn in wireframe representation. Protons, BAr^F24^ anions, as well as co‐crystallised solvent molecules, have been omitted for clarity. The coordination polyhedron of **1** around the central nickel atom deviates slightly from a perfect trigonal bipyramid due to tilting of the equatorial GaCp* units (Ga1–Ni1–Ga2: 81.898(13)°, Ga1–Ni1–Ga3: 85.173(13)°, Ga1–Ni1–Ga4: 86.667(14)°). (c) The gallium monofluoride moiety may be detected using ^19^F NMR spectroscopy as a comparably broad singlet at –79.9 ppm. (d) The Ga 2p spectrum as determined by XPS suggests the presence of exclusively Ga(I) centres in **1**.

Along this line, we sought to explore more challenging reactivity patterns, such as C–F bond activation, by our previously described, strong bimetallic Lewis acids [33]. When the Ni(II) complex [Ni(NC*^t^* Bu)_6_](BAr^F24^)_2_ (BAr^F24^: tetrakis[(bis‐3,5‐trifluoromethyl)phenyl]borate) is treated with five equivalents of GaCp* (Cp*: 1,2,3,4,5‐pentamethylcyclopentadienyl) in fluorobenzene, the reaction mixture immediately turns to an intense violet. A dark microcrystalline solid, which has been later identified as [(Cp*Ga)_4_Ni(NC*^t^* Bu)](BAr^F24^)_2_ (**2**), forms rapidly, accompanied by a pale‐yellow supernatant solution. Upon standing at room temperature for three months without agitation or alternatively by heating to 35° C for four days, the solution gradually changes colour to a dark orange. The initially formed compound **2** is consumed, ultimately yielding a bright orange solution and dark red single crystals of [(Cp*Ga)_4_Ni(GaF(NC*^t^* Bu)_2_)](BAr^F24^)_2_ (**1**). Single crystal x‐ray diffraction (SC‐XRD) of **1** revealed a five‐coordinate dicationic nickel complex in an all‐gallium coordination sphere. As a striking structural motif, **1** unexpectedly exhibits a yet unprecedented gallium monofluoride moiety including two *tert*‐butylnitrile ligands at the gallium centre (Figure 2a,b). The nickel atom in **1** is coordinated in a nearly perfect trigonal bipyramidal mode with four GaCp* metalloligands in total, that include Ga1 and Ga5 as the axial and Ga2, Ga3, and Ga4 as the equatorial ligands. With respect to the parent gallium monofluoride with a bond length of 1.774 Å in the gas phase [34], the Ga–F bond is slightly elongated to 1.8097(15) Å in **1**. For comparison, the Ga–F bond length in Ga(III) complexes with exclusively one fluoride ligand is also in a similar range of 1.797(2)–1.832(1) Å [35, 36]. In addition, a Ga(II) β‐diketiminate complex with a Ga–Ga bond exhibits a similar Ga–F bond length of 1.7726(12) Å [37]. The two axial gallium atoms in **1**, Ga1 and Ga5, are almost perfectly collinear to each other with an angle of 175.91(2)° that only slightly deviates from 180°. There is also a slight tilt of the equatorial plane with respect to the Ni1–Ga1 bond, which indicates strong σ‐donor character for the Ga–F moiety [38]. Similar effects have been calculated and observed in the seminal work on boron monofluoride as a ligand at an iron centre, which leads to a significant deviation from an ideal trigonal bipyramid as a coordination polyhedron [28]. Overall, [(Cp*Ga)_4_Ni(GaF(NC*^t^* Bu)_2_)](BAr^F24^)_2_ (**1**) is obtained reproducibly in a total yield of 51%, usually as one large, several millimetre‐sized single crystal. In fact, we expect the yield to be nearly quantitative, although the isolated yield is lower, most probably due to incomplete crystallisation and mechanical losses during isolation. Depending on the mode of crystallisation, two different solvatomorphs are obtained for **1** (see Supporting Information, Chapter 3) with either four fluorobenzene (*P*‐1) or two fluorobenzene molecules (*P*2_1_/*n*) co‐crystallised. The ^19^F NMR spectrum of **1** in 1,2‐difluorobenzene with 10 vol% C_6_D_6_ (Figure 2c) shows a broad singlet at –79.9 ppm, which corresponds to the gallium fluoride moiety. For comparison, structurally related Ga(III) complexes with one fluoride ligand, like the dimeric diorganogallium fluorides [Mes_2_GaF]_2_ (Mes: 2,3,5‐trimethylphenyl) and [(PhCH_2_)_2_GaF]_2_, which show bridging fluoride ligands, give rise to peaks in a range between –150 and –170 ppm in the ^19^F NMR spectrum [39]. Complexes with terminal fluoride ligands, like [Mes_2_GaF(NH_2_ *^t^* Bu)] [40] or Ga(III) fluorides with macrocyclic ligands [36] also exhibit similar chemical shifts, as well as the Ga–F moieties of formal Ga(II) complexes in a comparable range of –160 to –200 ppm [37]. Therefore, the vastly differing chemical shift for the ^19^F NMR peak of the gallium monofluoride unit in **1** nicely supports the +I oxidation state for the Ga centre. The +I oxidation state of Ga can also be inferred from XPS (X‐ray photoelectron spectroscopy) data. The Ga 2p spectrum of **1** (Figure 2d) exhibits peaks at binding energies of 1119.1 eV for the Ga 2p_3/2_ and 1146.0 eV for the Ga 2p_1/2_ signal. Previous XPS studies on organometallic gallium complexes show that Ga(I) compounds with differing ligand environments comprise comparable Ga 2p_3/2_ binding energies between 1117.5 and 1119.3 eV [41]. This suggests a +I oxidation state for the gallium atoms in [(Cp*Ga)_4_Ni(GaF(NC*^t^* Bu)_2_)](BAr^F24^)_2_ (**1**). Comparing the XPS data of **1** with a molecular reference compound bearing a formal Ni(II)/Ga(I) couple in a nearly identical coordination environment [33] yielded binding energies that were largely similar within the error of measurement and energy correction. A more detailed discussion, including reference compounds, is available in the Supporting Information. How closely we approach the “free” gas‐phase species GaF may also be deduced from the infrared (IR) spectrum of **1** in the solid state. While the free, diatomic GaF exhibits characteristic bands at 609–607 cm^−1^ in a solid neon matrix and 592–590 cm^−1^ in a solid argon matrix, respectively [42], our isolated gallium monofluoride complex **1** shows its Ga–F stretching vibration at only a slightly lower wavenumber of 589 cm^−1^ (Figure S31). Expectedly, due to its gallium centres in (formally) low oxidation numbers, **1** is extremely sensitive to air and moisture and decomposes indiscriminately in chlorinated or coordinating solvents such as dichloromethane or acetonitrile. Furthermore, **1** efficiently polymerises tetrahydrofuran within minutes due to its strong Lewis acidity.

### 2.2 Bonding Analysis of (**1**)

The bonding within the dicationic complex of **1** has been investigated using DFT calculations at the PBE0‐D3/TZ2P level (see Supporting Information). The optimised geometry matches well its SC‐XRD counterpart (Tables S3 and S4). The computed ^19^F NMR chemical shift (–72 ppm) is also consistent with its experimental counterpart (–79.9 ppm), as well as the Ga–F stretching vibrational band (589 cm^−1^ vs. 610 cm^−1^, Figures S7 and S31) in the IR spectrum. As expected, the atomic charges calculated through both a natural atomic orbital (NAO) population analysis [43] and the quantum theory of atoms in molecules (QTAIM) approach [44, 45] (Tables S5 and S6) are consistent with fairly polar Ni^δ−^‐Ga^δ+^ bonds, with the Ni–Ga1 one being the most polar. We also compared our stabilised gallium monofluoride complex with free GaF, its uncoordinated gas‐phase counterpart. For [(Cp*Ga)_4_Ni(GaF(NC*^t^* Bu)_2_)]^2+^, the optimised Ga–F bond length is almost equal to that computed for free GaF (∼1.79 Å, Tables S5 and S7) and its bond critical point (bcp) descriptors are also close to those of free GaF.

To evaluate the energetics of the metal‐ligand interaction in [(Cp*Ga)_4_Ni(GaF(NC*^t^* Bu)_2_)]^2+^, an energy decomposition analysis (EDA) of the interaction between two frozen molecular fragments was performed according to the Morokuma‐Ziegler procedure (see Table 1) [46, 47, 48]. The two considered fragments are the GaF(NC*^t^* Bu)_2_ ligand and the [(Cp*Ga)_4_Ni]^2+^ unit. The computed *E*_elstat_ and *E*_orb_ values are characteristic of a polar‐covalent bond in which electrostatic interactions dominate [49]. Formally substituting the GaF(NC*^t^* Bu)_2_ ligand by GaCp* generates the homoleptic [(Cp*Ga)_5_Ni]^2+^ complex for which the EDA values (Table 1) indicate substantially weaker overall bonding.

**TABLE 1:** Morokuma‐Ziegler energy decomposition analysis in [(Cp*Ga)_4_NiL]^2+^ (L = GaF(NC*^t^* Bu)_2_, GaCp*, and GaF). The total bonding energy (TBE) is the sum of four components, *E*_Pauli_, *E*_elstat_, *E*_orb_, and *E*_disp_, which are associated with Pauli repulsion, electrostatic interaction, orbital interaction, and dispersion forces, respectively. All values in eV.

Complex	[(Cp*Ga)_4_Ni(GaF(NC*^t^* Bu)_2_)]^2+^	[(Cp*Ga)_5_Ni]^2+^	[(Cp*Ga)_4_Ni(GaF)]^2+^
Fragmentation	GaF(NC*^t^* Bu)_2 _+ [(Cp*Ga)_4_Ni]^2+^	GaCp* + [(Cp*Ga)_4_Ni]^2+^	GaF + [(Cp*Ga)_4_Ni]^2+^
*E*_Pauli_	9.41	6.40	7.28
*E*_elstat_	−8.64	−4.74	−5.28
*E*_orb_	−5.56	−3.96	−4.43
*E*_disp_	−0.88	−0.83	−0.33
**TBE**	**—5.68**	**—3.12**	**—2.75**

**FIGURE 3:**
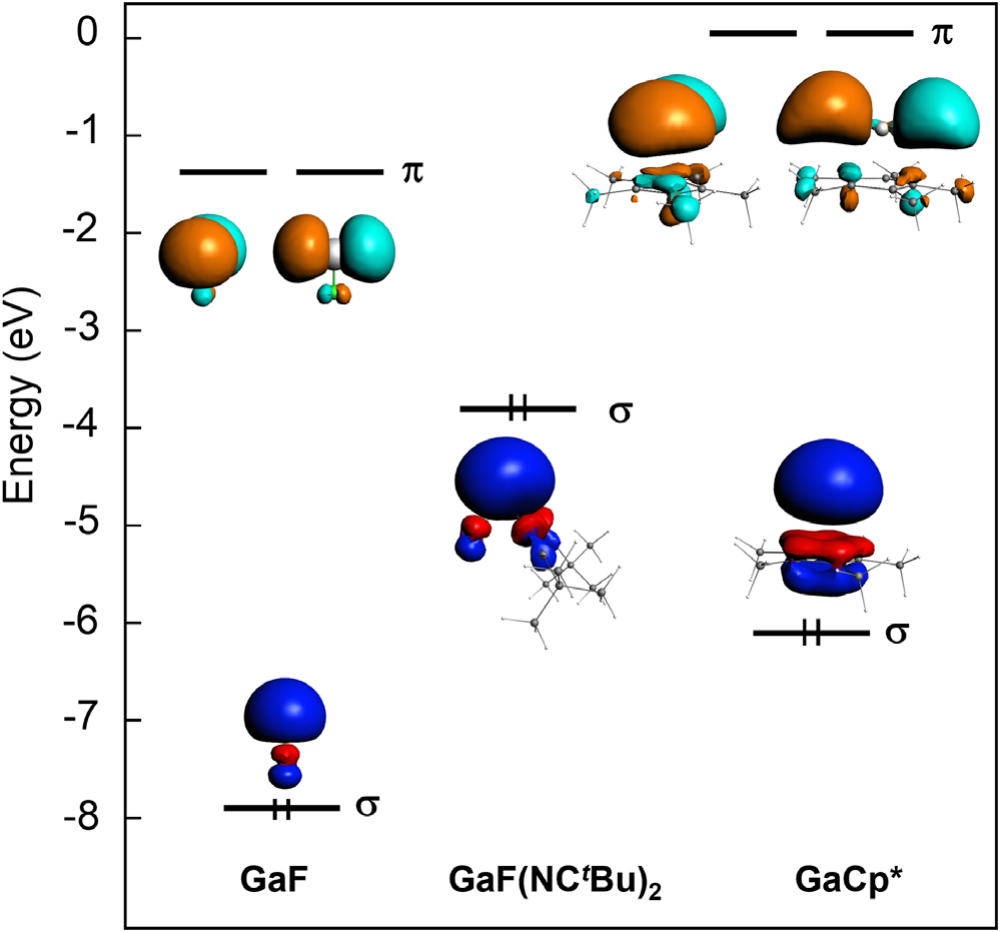
The frontier orbitals of the GaF, GaF(NC^t^Bu)_2,_ and GaCp* metalloligands. Contrary to GaF and GaCp*, GaF(NC*^t^* Bu)_2_ has no accepting π‐type orbitals, but it is a significantly better σ‐donor.

The Ga–N distances in [(Cp*Ga)_4_Ni(GaF(NC*^t^* Bu)_2_)]^2+^ (∼2.1 Å) are somewhat long, and the corresponding QTAIM descriptors (Table S3) suggest a not‐very‐strong bonding to Ga1. Once again, this shows how closely we approach the free gas‐phase species GaF in our complex. This is also confirmed by EDA calculations, which lead to a moderate value of TBE = –1.47 eV for the bond between one NC*^t^* Bu ligand and the rest of the molecule. Therefore, we have also investigated the “de‐nitrilated” hypothetical complex [(Cp*Ga)_4_Ni(GaF)]^2+^. Surprisingly, the Ga–F distance in this species is shorter than that in free GaF, whereas the fluorine charge barely changes (Tables S5 and S7). This is attributed to the GaF HOMO (the Ga lone pair) having a certain Ga–F antibonding character, which is reduced upon its depopulation when coordinated to the nickel centre. The various other computed Ga–F indicators are of the same order of magnitude as in [(Cp*Ga)_4_Ni(GaF(NC*^t^* Bu)_2_)]^2+^ (Table 1). In the other end, the EDA components associated with the bonding of GaF to [Ni(GaCp*)_4_]^2+^ appear not to be very different from those obtained for the GaCp* ligand (Table 1), indicating that GaF and GaCp* are equally strong ligands, though significantly weaker ligands than GaF(NC*^t^* Bu)_2_. The difference between the GaF and GaF(NC*^t^* Bu)_2_ ligand strengths originates in part from the considerably higher energy of the Ga lone pair in the case of GaF(NC*^t^* Bu)_2_ (Figure 3) and in part from the larger 4p character of this (4s‐dominated) σ‐type orbital, which allows for a better overlap with the metal accepting orbital (lone pair occupation after interaction: 1.36e and 0.99e for GaF and GaCp*, respectively). On the other hand, contrary to GaF(NC*^t^* Bu)_2_, the GaF ligand has accepting orbitals of 98% 4p_π_(Ga) composition (Figure 3), allowing metal‐to‐ligand back bonding (occupations 2 x 0.26e after interaction). Still, as for the GaCp* ligand (Figure 3), this effect is not sufficient for making the │*E_orb_* │ component of GaF larger than that of GaF(NC*^t^* Bu)_2_ (Table 1). Moreover, the electron‐attracting power of F on GaF tends to reduce │*E_elstat_* │ to a larger extent than the two *tert*‐butylnitrile and the fluorine substituents in the case of GaF(NC*^t^* Bu)_2_ [49]. The fact that the GaF(NC*^t^* Bu)_2_ ligand is the strongest in the coordination sphere of nickel is consistent with our experimental findings on removing the nitrile ligands from gallium. Although the nitrile ligands appear to be comparably weakly bound, “de‐nitrilation” of [(Cp*Ga)_4_Ni(GaF(NC*^t^* Bu)_2_)](BAr^F24^)_2_ (**1**) by either heating under vacuum or applying strong Lewis acids like B(C_6_F_5_)_3_ fails and produces only decomposition products, but no hypothetical, nitrile‐free complex.

### 2.3 Mechanistic Aspects for the Formation of (**1**)

**FIGURE 4:**
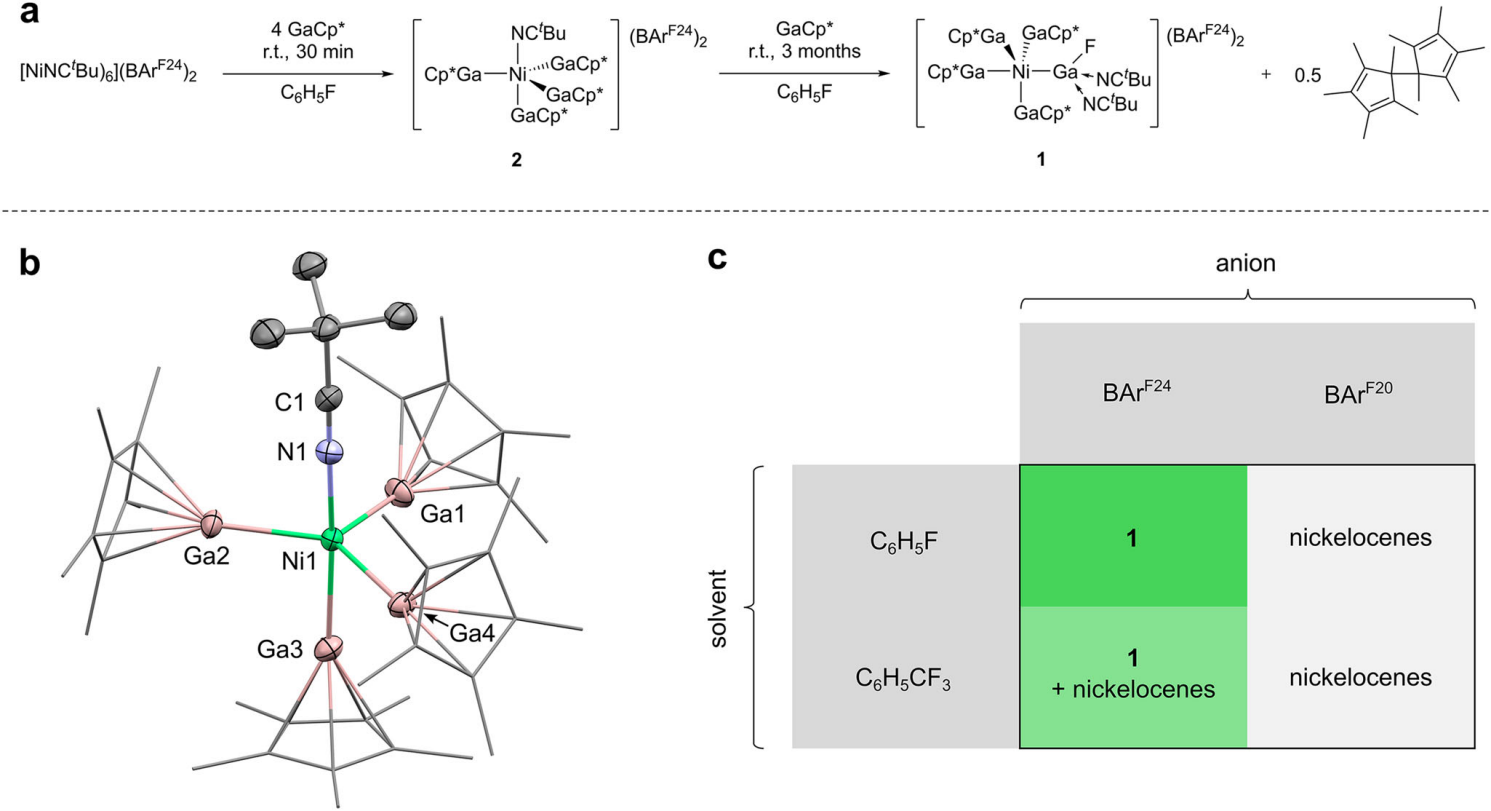
(a) Reaction sequence for the synthesis of the gallium monofluoride complex **1** with [(Cp*Ga)_4_Ni(NC*^t^* Bu)](BAr^F24^)_2_ (**2**) as the intermediate. (b) Molecular structure of the dicationic part of **2** as determined by SC‐XRD. Ellipsoids are drawn at 50% probability; Cp* ligands are drawn in wireframe representation. Protons, BAr^F24^ anions, as well as co‐crystallised solvent molecules, have been omitted for clarity. Selected bond lengths [Å] and angles [°]: Ni1–Ga1: 2.3344(10), Ni1–Ga2: 2.2417(11), Ni1–Ga3: 2.2500(11), Ni1–Ga4: 2.3353(11), Ni1–N1: 1.906(11); Ga1–Ni1–Ga2: 115.07(5), Ga4–Ni1–Ga1: 113.29(4), Ga4–Ni1–Ga2: 131.23(5). (c) Combinatorial approach for identifying the weakly coordinating anion BAr^F24^ as the fluorine source for the formation of **1**.

SC‐XRD experiments on the isolated material forming at the beginning of the reaction confirmed its identity as [(Cp*Ga)_4_Ni(NC*^t^* Bu)](BAr^F24^)_2_ (**2**) (Figure 4). The nickel atom in **2** adopts a distorted trigonal bipyramidal coordination geometry with Ga1, Ga2 and Ga3 as equatorial and Ga4 as well as the *tert*‐butylnitrile moiety as the axial ligand. With the weakly coordinated nitrile ligand, **2** may be interpreted as a synthon for a highly electrophilic and Lewis acidic 16‐electron species [Ni(GaCp*)_4_]^2+^. This is also reflected in its high acceptor number (AN) of 93–98, as calculated by the Gutmann‐Beckett method [50, 51] (Figure S19). For comparison, common strong Lewis acids like SbCl_5_ (AN = 100) or BF_3_•OEt_2_ (AN = 89) exhibit acceptor numbers in a similar range [50, 51]. Consistently, the fluoride ion affinity (FIA) of [(Cp*Ga)_4_Ni(NC*^t^* Bu)]^2+^ of 818 kJ mol^−1^, computed according to the approach proposed by Lehmann et al. [52] (details see Supplementary Information), approaches that found for the trimethylsilylium cation at the same computational level (920 kJ mol^−1^). A redox‐neutral C–F bond cleavage *via* fluoride abstraction is indeed a type of reactivity for such strong Lewis acids [53, 54]. We attribute this strong Lewis acidity to synergistic effects between nickel and gallium in the metal‐rich environment of **2** [33, 55].

In principle, both the solvent fluorobenzene (aromatic C–F) and the anion BAr^F24^ (aliphatic C–F), may serve as fluoride sources. To unambiguously disclose the substrate for C–F bond activation, we performed experiments using different solvents or anions. Notably, isotopic labelling is not possible for fluorine, as ^19^F is the only naturally abundant stable isotope. Therefore, we tested the closely related anion BAr^F20^ (tetrakis(pentafluorophenyl)borate), which exhibits exclusively C(sp^2^)–F bonds, as well as trifluorotoluene as the solvent, which contains only C(sp^3^)–F bonds. These experiments clearly demonstrate that the anion BAr^F24^ serves as the fluoride source for the formation of **1**: the reaction only occurs in the presence of BAr^F24^, regardless of whether fluorobenzene or trifluorotoluene is used as the solvent. For BAr^F20^, no formation of **1** was observed at all (Table S1) by SC‐XRD, but only nickelocene species were detected as undesirable side products. Strikingly, the reaction also proceeds with BAr^F24^ as the anion in chlorobenzene as the solvent, despite the non‐negligible formation of nickelocenes as observed by SC‐XRD. This clearly shows the anion as the fluorine source, while eliminating the solvent as a potential source. We propose that an intimate ion pair of intermediate **2** and its BAr^F24^ anions in fluorobenzene solution is a crucial step in forming **1**. This is supported by ^1^H DOSY (diffusion‐ordered spectroscopy) NMR spectra of the intermediate **2** in a mixture of fluorobenzene and 1,2‐difluorobenzene, which suggests an identical diffusion coefficient for both the BAr^F24^ anions and the dication of **2** (Figure S23). In situ UV–vis studies also indicate the appearance of the characteristic bands of **1** (Figures S25 and S26), suggesting that C(sp^3^)–F bond activation occurs in fluorobenzene solution. It should be noted that we were unable to detect partially defluorinated CF_3_ groups using SC‐XRD. Presumably, the missing fluorine atom is statistically distributed within the whole single crystal over the 48 fluorine atom positions that two BAr^F24^ anions exhibit. Instead, while monitoring the reaction using in situ ^1^H NMR spectroscopy (Figure S22), we observed the formation of (Cp*)_2_ (dihydrodecamethylfulvalene) as a characteristic product for radical coupling of two Cp* fragments. Therefore, a putative radical pathway for the formation of **1** cannot be entirely excluded. Nevertheless, the reaction is induced thermally and does not proceed *via* a photochemical pathway. Strict exclusion of light leads to [(Cp*Ga)_4_Ni(GaF(NC*^t^* Bu)_2_)](BAr^F24^)_2_ (**1**) as the sole product with identical spectroscopic data and yield.

### 2.4 Fluoride Transfer to Silicon and Carbon Electrophiles

**FIGURE 5:**
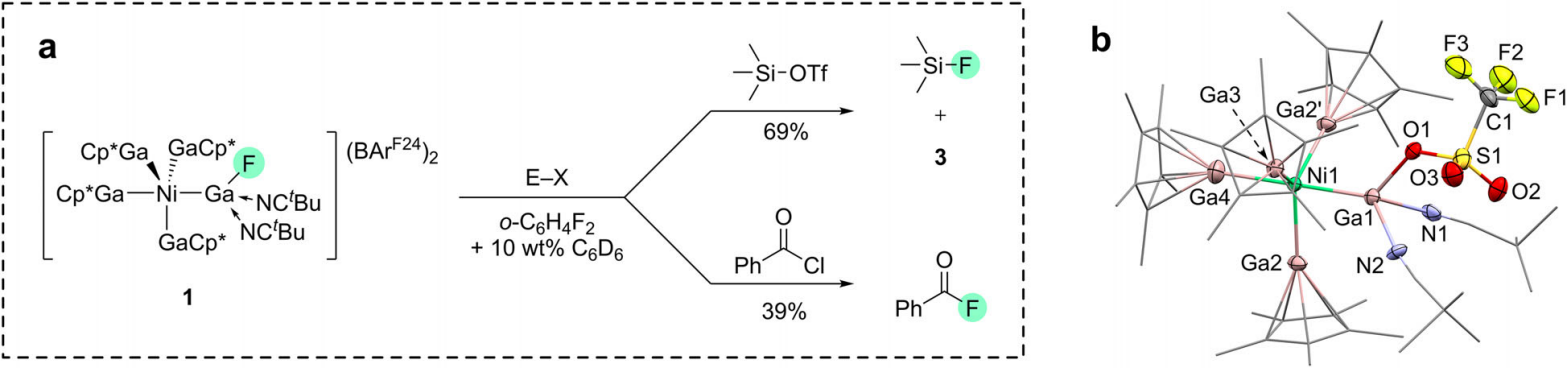
(a) Fluoride transfer reactions of **1** to trimethylsilyl triflate and benzoyl chloride to form TMSF and benzoyl fluoride (yields determined by ^19^F NMR spectroscopy). (b) Molecular structure of the dicationic fragment of [(Cp*Ga)_4_Ni(GaOTf(NC*^t^* Bu)_2_)](BAr^F24^)_2_ (**3**) as determined by SC‐XRD.

While the gallium monofluoride moiety of [(Cp*Ga)_4_Ni(GaF(NC*^t^* Bu)_2_)](BAr^F24^)_2_ (**1**) stems from C(sp^3^)–F bond activation, it may also serve as a fluorine donor itself. Thus, it can be defluorinated using, for example, silicon‐containing electrophiles such as trimethylsilyl triflate (TMSOTf) as a fluoride scavenger (Figure 5). Upon exposure of a 1,2‐difluorobenzene solution of **1** to TMSOTf at room temperature, a distinct colour change to dark red is observed immediately. *In situ* ^1^ ^9^F NMR spectra with excess TMSOTf prove the concomitant formation of fluorotrimethylsilane (TMSF, 69% yield based on NMR spectroscopy) as a characteristic side product (Figure S27). Layering the reaction solution with *n*‐hexane at –35° C produced a few single crystals, which were identified as the corresponding triflate complex [(Cp*Ga)_4_Ni(GaOTf(NC*^t^* Bu)_2_)](BAr^F24^)_2_ (**3**). The solid‐state structure of **3** shows that the complex remains intact, with only the fluorine atom of the GaF moiety cleanly replaced by a triflate anion. Similar to **1**, compound **3** shows a nearly perfect trigonal bipyramidal coordination sphere around the central nickel atom, as well as the gallium triflate moiety occupying one of the two axial positions. Notably, the Ga–O bond length of 1.88(4) Å in **3** is significantly shorter than for the parent GaOTf@18‐crown‐6 complex with 2.137(1) Å in the solid state [56]. In addition to silicon‐based electrophiles, the fluorine atom of the GaF moiety may be transferred to carbon atoms as well. Reacting **1** with excess benzoyl chloride at room temperature furnishes benzoyl fluoride as the sole detectable product in 39% yield after 15 min, as determined by ^19^F NMR spectroscopy.

## 3 Conclusion

To conclude, we have described a complex bearing an unprecedented gallium monofluoride moiety in the coordination sphere of a formal Ni(II) centre. The gallium fluoride unit stems from C(sp^3^)–F bond activation of BAr^F24^ as a weakly coordinating anion and acts as a potent σ‐donor ligand. The ligand strength of GaF(NC*^t^* Bu)_2_ exceeds the donor capabilities of GaCp* according to DFT calculations. Besides these properties as a metalloligand at nickel, the GaF moiety may be defluorinated using TMSOTf to form fluorotrimethylsilane and benzoyl fluoride, starting from benzoyl chloride, respectively. For the future, we anticipate testing the described bimetallic nickel‐gallium system for defluorination of highly inert per‐ and polyfluorinated alkyl substances (PFAS) *via* C–F bond activation.

## Author Contributions

**Johannes Stephan**: investigation, writing – original draft, data curation, formal analysis, visualisation, conceptualisation, project administration, methodology. **Samia Kahlal**: formal analysis, methodology, visualisation. **Jean‐Yves Saillard**: formal analysis, methodology, project administration, visualisation, conceptualisation, supervision, writing – original draft. **Marvin Mühlau**: investigation, formal analysis. **Lisa Gremmel**: investigation. **Christian Gemel**: conceptualisation, writing – review editing, supervision. **Roland A. Fischer**: conceptualissation, funding acquisition, writing – review editing, supervision, resources.

## Conflicts of Interest

The author declares no conflicts of interest.

## Supporting information



The authors have cited additional references within the Supporting Information [57, 58, 59, 60, 61, 62, 63, 64, 65, 66, 67, 68, 69, 70, 71, 72, 73, 74, 75, 76, 77, 78, 79, 80, 81, 82, 83, 84, 85, 86, 87].**Supporting File 1**: anie73191‐sup‐0001‐SuppMat.pdf.

**Supporting File 2**: anie73191‐sup‐0002‐SuppMat.xyz

## Data Availability

The data that support the findings of this study are available from the corresponding author upon reasonable request.

## References

[1] L. Mond , C. Langer , and F. Quincke , “L.—Action of Carbon Monoxide on Nickel,” Journal of the Chemical Society, Transactions 57 (1890): 749–753, 10.1039/CT8905700749.

[2] D. Singh , W. R. Buratto , J. F. Torres , and L. J. Murray , “Activation of Dinitrogen by Polynuclear Metal Complexes,” Chemical Reviews 120, no. 12 (2020): 5517–5581, 10.1021/acs.chemrev.0c00042. 32364373 PMC7730004

[3] T. P. Hanusa , “Cyanide Complexes of the Transition Metals,” in Encyclopedia of Inorganic Chemistry (2005), 10.1002/0470862106.ia059.

[4] M. Elian , M. M. L. Chen , D. M. P. Mingos , and R. Hoffmann , “Comparative Bonding Study of Conical Fragments,” Inorganic Chemistry 15, no. 5 (1976): 1148–1155, 10.1021/ic50159a034.

[5] R. Hoffmann , “Building Bridges Between Inorganic and Organic Chemistry (Nobel Lecture),” Angewandte Chemie International Edition 21, no. 10 (1982): 711–724, 10.1002/anie.198207113.

[6] F. K. Levin and J. G. Winans , “The Absorption Spectrum of GaCl,” Physical Review 84, no. 3 (1951): 431–440, 10.1103/PhysRev.84.431.

[7] H. Schnöckel and H. J. Gocke , “Matrixreaktion von GaF mit O‐Atomen: IR‐Spektrum von OGaF,” Journal of Molecular Structure 50, no. 2 (1978): 281–284, 10.1016/0022-2860(78)80086-6.

[8] K. P. R. Nair , H. U. Schütze‐Pahlmann , and J. Hoeft , “Millimeter‐Wave Rotational Spectrum and Molecular Constants of Diatomic Gallium Bromide,” Chemical Physics Letters 80, no. 1 (1981): 149–152, 10.1016/0009-2614(81)80078-4.

[9] W. B. Griffith , G. A. Bickel , H. D. McSwiney , and C. W. Mathews , “An Analysis of the Rotational Structure in the C^1^Π‐X^1^Σ^+^ System of GaF,” Journal of Molecular Spectroscopy 104, no. 2 (1984): 343–346, 10.1016/0022-2852(84)90127-9.

[10] R. Honerjäger and R. Tischer , “Magnetische Konstanten der Molekeln CuF und GaF /Magnetic Constants of the CuF and GaF Molecule,” Zeitschrift für Naturforschung Teil A, Physik, physikalische Chemie, Kosmophysik 29, no. 12 (1974): 1919–1921, 10.1515/zna-1974-1234.

[11] J. Hoeft , F. J. Lovas , E. Tiemann , and T. Törring , “On the Production and Stability of Some Group III a Monofluorides,” Zeitschrift für Naturforschung. B, Chemie, Biochemie, Biophysik, Biologie und verwandte Gebiete 25, no. 9 (1970): 901, 10.1515/znb-1970-0901.

[12] D. Welti and R. F. Barrow , “The Absorption Spectra of Gallium and Indium Monofluorides,” Proceedings of the Physical Society. Section A 65, no. 8 (1952): 629–639, 10.1088/0370-1298/65/8/306.

[13] N. N. Greenwood and A. Earnshaw , Chemistry of the Elements (Butterworth‐Heinemann, Oxford, 1995), pp. 261–271, 10.1016/C2009-0-30414-6.

[14] A. Haupt , Organic and Inorganic Fluorine Chemistry—Methods and Applications (Walter de Gruyter GmbH, 2021), pp.64–65, 10.1515/9783110659337.

[15] P. L. Timms , “Chemistry of Boron and Silicon Subhalides,” Accounts of Chemical Research 6, no. 4 (1973): 118–123, 10.1021/ar50064a002.

[16] M. Mocker , C. Robl , and H. Schnöckel , “Donor‐Stabilized Aluminum(I) Bromide,” Angewandte Chemie International Edition 33, no. 17 (1994): 1754–1755, 10.1002/anie.199417541.

[17] A. Ecker and H. Schnöckel , “Donorstabilisiertes Aluminium(I)‐Iodid,” Zeitschrift für anorganische und allgemeine Chemie 622, no. 1 (1996): 149–152, 10.1002/zaac.19966220120.

[18] T. Duan , P. Henke , G. Stöβer , Q.‐F. Zhang , and H. Schnöckel , “Ga_8_Br_8_·6NEt_3_: Formation and Structure of Donor‐Stabilized GaBr. A Nanoscaled Step on the Way to β‐Gallium?,” Journal of the American Chemical Society 132, no. 4 (2010): 1323–1327, 10.1021/ja907060q. 20063876

[19] C. U. Doriat , M. Friesen , E. Baum , A. Ecker , and H. Schnöckel , “Synthesis, Structure, and Oxidation of Donor‐Stabilized Gallium(I) Iodide: Ga_8_I_8_ · 6 PEt_3_ ,” Angewandte Chemie International Edition in English 36, no. 18 (1997): 1969–1971, 10.1002/anie.199719691.

[20] M. Tacke and H. Schnöckel , “Metastable Aluminum Chloride, AlCl, as a Solid and in Solution,” Inorganic Chemistry 28, no. 14 (1989): 2895–2896, 10.1021/ic00313a039.

[21] M. Tacke , H. Kreienkamp , L. Plaggenborg , and H. Schnöckel , “GaCl: Ein roter, Metastabiler Festkörper,” Zeitschrift für anorganische und allgemeine Chemie 604, no. 1 (1991): 35–38, 10.1002/zaac.19916040105.

[22] C. Dohmeier , C. Robl , M. Tacke , and H. Schnöckel , “The Tetrameric Aluminum(I) Compound [{Al(η^5^‐C_5_Me_5_)}_4_],” Angewandte Chemie International Edition 30, no. 5 (1991): 564–565, 10.1002/anie.199105641.

[23] D. Loos and H. Schnöckel , “(Cyclopentadienyl)‐Gallium(I)‐Verbindungen,” Journal of Organometallic Chemistry 463, no. 1 (1993): 37–40, 10.1016/0022-328X(93)83395-C.

[24] J. Vollet , J. R. Hartig , and H. Schnöckel , “Al_50_C_120_H_180_: A Pseudofullerene Shell of 60 Carbon Atoms and 60 Methyl Groups Protecting a Cluster Core of 50 Aluminum Atoms,” Angewandte Chemie International Edition 43, no. 24 (2004): 3186–3189, 10.1002/anie.200453754. 15199573

[25] M. Huber , A. Schnepf , C. E. Anson , and H. Schnöckel , “Si@Al_56_[N(2,6‐*i*Pr_2_C_6_H_3_)SiMe_3_]_12_: The Largest Neutral Metalloid Aluminum Cluster, a Molecular Model for a Silicon‐Poor Aluminum–Silicon Alloy?,” Angewandte Chemie International Edition 47, no. 43 (2008): 8201–8206, 10.1002/anie.200801585. 18814152

[26] A. Schnepf , G. Stößer , and H. Schnöckel , “[Ga_22_{N(SiMe_3_)_2_}_10_]^2−^: A Metalloid Cluster Compound With a Variation of the Ga_22_ Framework,” Angewandte Chemie International Edition 41 , no. 11 (2002): 1882, 10.1002/1521-3773(20020603)41:11<1882::AID-ANIE1882>3.0.CO;2-N. 19750621

[27] A. Schnepf and H. Schnöckel , “Metalloid Aluminum and Gallium Clusters: Element Modifications on the Molecular Scale?,” Angewandte Chemie International Edition 41, no. 19 (2002): 3532–3554, 10.1002/1521-3773(20021004)41:19<3532::AID-ANIE3532>3.0.CO;2-4. 12370894

[28] M. J. Drance , J. D. Sears , A. M. Mrse , et al., “Terminal Coordination of Diatomic Boron Monofluoride to Iron,” Science 363, no. 6432 (2019): 1203–1205, 10.1126/science.aaw6102. 30872521

[29] N. D. Coombs , W. Clegg , A. L. Thompson , D. J. Willock , and S. Aldridge , “A Group 13/Group 17 Analogue of CO and N_2_: Coordinative Trapping of the GaI Molecule,” Journal of the American Chemical Society 130, no. 16 (2008): 5449–5451, 10.1021/ja800876k. 18376829

[30] N. D. Coombs , D. Vidovic , J. K. Day , et al., “Cationic Terminal Gallylene Complexes by Halide Abstraction: Coordination Chemistry of a Valence Isoelectronic Analogue of CO and N_2_ ,” Journal of the American Chemical Society 130 (2008): 16111–16124, 10.1021/ja806655f. 18975947

[31] D. Vidovic and S. Aldridge , “Coordination and Activation of the BF Molecule,” Angewandte Chemie International Edition 48, no. 20 (2009): 3669–3672, 10.1002/anie.200901022. 19373822

[32] H. Braunschweig and M. Müller , “New Borylene Complexes of the Type [μ‐BX{μ^5^‐C_5_H_4_Me)Mn(CO)_2_}_2_]: Substitution Reactions at the Metal‐Coordinated Borylene Moiety,” Chemische Berichte 130, no. 9 (1997): 1295–1298, 10.1002/cber.19971300918.

[33] J. Stephan , R. Bühler , F. E. Napoli , C. Gemel , and R. A. Fischer , “Cooperative Reactivity Induced by All‐Gallium Coordination at Nickel,” Inorganic Chemistry 64, no. 30 (2025): 15707–15714, 10.1021/acs.inorgchem.5c02296. 40679271 PMC12326349

[34] R. E. Wasylishen , D. L. Bryce , C. J. Evans , and M. C. L. Gerry , “Hyperfine Structure in the Rotational Spectrum of GaF: A Comparison of Experimental and Calculated Spin–Rotation and Electric Field Gradient Tensors,” Journal of Molecular Spectroscopy 204, no. 2 (2000): 184–194, 10.1006/jmsp.2000.8211. 11148088

[35] D. E. Runacres , V. K. Greenacre , J. M. Dyke , et al., “Synthesis, Characterization, and Computational Studies on Gallium(III) and Iron(III) Complexes With a Pentadentate Macrocyclic *Bis*‐Phosphinate Chelator and Their Investigation as Molecular Scaffolds for ^18^F Binding,” Inorganic Chemistry 62, no. 50 (2023): 20844–20857, 10.1021/acs.inorgchem.3c03135. 38055373 PMC10731642

[36] H. Koay , M. B. Haskali , P. D. Roselt , J. M. White , and P. S. Donnelly , “Gallium Fluoride Complexes With Acyclic Bispicolinic Ligands as Potential New Fluorine‐18 Labelled Imaging Agents,” European Journal of Inorganic Chemistry 35, no. 2020 (2020): 3378–3386, 10.1002/ejic.202000547.

[37] O. Kysliak , H. Görls , and R. Kretschmer , “Cooperative Bond Activation by a Bimetallic Main‐Group Complex,” Journal of the American Chemical Society 143, no. 1 (2021): 142–148, 10.1021/jacs.0c12166. 33356229

[38] U. Radius , F. M. Bickelhaupt , A. W. Ehlers , N. Goldberg , and R. Hoffmann , “Is CO a Special Ligand in Organometallic Chemistry? Theoretical Investigation of AB, Fe(CO)_4_AB, and Fe(AB)_5_ (AB = N_2_, CO, BF, SiO),” Inorganic Chemistry 37, no. 5 (1998): 1080–1090, 10.1021/ic970897.

[39] B. Neumüller and F. Gahlmann , “Diorganogalliumfluoride. Die Kristallstruktur des Mischkristalls [B(CH_2_Ph)_3_]_0,92_[Ga(CH_2_Ph)_3_]_0,08_ · NCMe,” Zeitschrift für anorganische und allgemeine Chemie 612, no. 6 (1992): 123–129, 10.1002/zaac.19926120121.

[40] T. Kräuter and B. Neumüller , “Reaktionen von Diorganogallium(indium)fluoriden. Die Kristallstruktur von Mes_2_InF,” Zeitschrift für anorganische und allgemeine Chemie 621, no. 4 (1995): 597–606, 10.1002/zaac.19956210417.

[41] J. L. Bourque , M. C. Biesinger , and K. M. Baines , “Chemical State Determination of Molecular Gallium Compounds Using XPS,” Dalton Transactions 45, no. 18 (2016): 7678–7696, 10.1039/C6DT00771F. 27052931

[42] J. W. Hastie , R. H. Hauge , and J. L. Margarve , “Infrared Spectra of Matrix‐Isolated Species in the Gallium‐Fluorine System,” Journal of Fluorine Chemistry 3, no. 3 (1974): 285–291, 10.1016/S0022-1139(00)82628-7.

[43] J. Badenhoop , A. Reed , J. Carpenter , J. Bohmann , C. Morales , and F. Weinhold , NBO 6.0, University of Wisconsin, Madison (WI), 2001.

[44] J. I. Rodríguez , “An Efficient Method for Computing the QTAIM Topology of a Scalar Field: The Electron Density Case,” Journal of Computational Chemistry 34 (2013): 681–686, 10.1002/jcc.23180. 23175458

[45] J. I. Rodríguez , R. F. W. Bader , P. W. Ayers , C. Michel , A. W. Götz , and C. Bo , “A High Performance Grid‐Based Algorithm for Computing QTAIM Properties,” Chemical Physics Letters 472, no. 1 (2009): 149–152, 10.1016/j.cplett.2009.02.081.

[46] F. M. Bickelhaupt and E. J. Baerends , “Kohn‐Sham Density Functional Theory: Predicting and Understanding Chemistry,” Reviews in Computational Chemistry 15 (2000): 1–86.

[47] K. Morokuma , “Molecular Orbital Studies of Hydrogen Bonds. III. C=O···H–O Hydrogen Bond in H_2_CO···H_2_O and H_2_CO···2H_2_O,” Journal of Chemical Physics 55 (1971): 1236–1244, 10.1063/1.1676210.

[48] T. Ziegler and A. Rauk , “A Theoretical Study of the Ethylene‐Metal Bond in Complexes Between Copper(1+), Silver(1+), Gold(1+), Platinum(0) or Platinum(2+) and Ethylene, Based on the Hartree‐Fock‐Slater Transition‐State Method,” Inorganic Chemistry 18 (1979): 1558–1565, 10.1021/ic50196a034.

[49] J. Uddin , C. Boehme , and G. Frenking , “Nature of the Chemical Bond Between a Transition Metal and a Group‐13 Element: Structure and Bonding of Transition Metal Complexes With Terminal Group‐13 Diyl Ligands ER (E = B to Tl; R = Cp, N(SiH_3_)_2_, Ph, Me),” Organometallics 19, no. 4 (2000): 571–582, 10.1021/om990936k.

[50] V. Gutmann , “Solvent Effects on the Reactivities of Organometallic Compounds,” Coordination Chemistry Reviews 18, no. 2 (1976): 225–255, 10.1016/S0010-8545(00)82045-7.

[51] M. A. Beckett , G. C. Strickland , J. R. Holland , and K. Sukumar Varma , “A Convenient N.M.R. Method for the Measurement of Lewis Acidity at Boron Centres: Correlation of Reaction Rates of Lewis Acid Initiated Epoxide Polymerizations With Lewis Acidity,” Polymer 37, no. 20 (1996): 4629–4631, 10.1016/0032-3861(96)00323-0.

[52] M. Lehmann , S. N. Balogun , M. Reimann , and M. Kaupp , “The Fluoride Ion Affinity Revisited: Do We Need the Anchor‐Point Approach?,” Chemistry – A European Journal 31, no. 26 (2025): e202404662, 10.1002/chem.202404662. 40028678 PMC12063057

[53] M. Kira , T. Hino , and H. Sakurai , “An NMR Study of the Formation of Silyloxonium Ions by Using Tetrakis[3,5‐bis(trifluoromethyl)phenyl]borate as Counteranion,” Journal of the American Chemical Society 114 (1992): 6697–6700, 10.1021/ja00043a013.

[54] C. Douvris and O. V. Ozerov , “Hydrodefluorination of Perfluoroalkyl Groups Using Silylium‐Carborane Catalysts,” Science 321 (2008): 1188–1190, 10.1126/science.1159979. 18755971

[55] J. Campos , “Bimetallic Cooperation Across the Periodic Table,” Nature Reviews Chemistry 4, no. 12 (2020): 696–702, 10.1038/s41570-020-00226-5. 37127975

[56] J. T. Boronski , M. P. Stevens , B. van IJzendoorn , A. C. Whitwood , and J. M. Slattery , “Insights Into the Composition and Structural Chemistry of Gallium(I) Triflate,” Angewandte Chemie International Edition 60, no. 3 (2021): 1567–1572, 10.1002/anie.202010837. 33022877 PMC7839670

[57] Deposition numbers 2500330–2500337 and 2535108 contain the supplementary crystallographic data for this paper. These data are provided free of charge by the joint Cambridge Crystallographic Data Centre and Fachinformationszentrum Karlsruhe Access Structures service.

[58] W. L. F. Armarego and D. D. Perrin , Purification of Laboratory Chemicals (Elsevier Science & Technology Books, 1997).

[59] P. Jutzi and L. O. Schebaum , “A Novel Synthetic Route to Pentaalkylcyclopentadienylgallium(I) Compounds,” Journal of Organometallic Chemistry 654 (2002): 176–179, 10.1016/S0022-328X(02)01429-8.

[60] R. A. Heintz , J. A. Smith , P. S. Szalay , A. Weisgerber , and K. R. Dunbar , Inorganic Syntheses 33 (2002): 75–121.

[61] N. A. Yakelis and R. G. Bergman , “Safe Preparation and Purification of Sodium Tetrakis[(3,5‐trifluoromethyl)phenyl]borate (NaBArF_24_): Reliable and Sensitive Analysis of Water in Solutions of Fluorinated Tetraarylborates,” Organometallics 24 (2005): 3579–3581, 10.1021/om0501428. 19079785 PMC2600718

[62] C. R. Smith , A. Zhang , D. J. Mans , and V. T. RajanBabu , “(R)‐3‐Methyl‐3‐phenyl‐1‐pentene *via* Catalytic Asymmetric Hydrovinylation,” Organic Syntheses 85 (2008): 248–266, 10.1002/0471264229. PMC2723857 19672483

[63] M. Brookhart , B. Grant , and A. F. Volpe Jr. , “[(3,5‐(CF_3_)_2_C_6_H_3_)_4_B]‐[H(OEt_2_)_2_]^+^: A Convenient Reagent for Generation and Stabilization of Cationic, Highly Electrophilic Organometallic Complexes,” Organometallics 11 (1992): 3920–3922, 10.1021/om00059a071.

[64] L. Omann and M. Oestreich , “Catalytic Access to Indole‐Fused Benzosiloles by 2‐Fold Electrophilic C–H Silylation With Dihydrosilanes,” Organometallics 36 (2017): 767–776, 10.1021/acs.organomet.6b00801.

[65] D. Roth , J. Stirn , D. W. Stephan , and L. Greb , “Lewis Superacidic Catecholato Phosphonium Ions: Phosphorus–Ligand Cooperative C–H Bond Activation,” Journal of the American Chemical Society 143 (2021): 15845–15851, 10.1021/jacs.1c07905. 34521202

[66] M. Olschewski , R. Gustus , M. Marschewski , O. Höfft , and F. Endres , “Spectroscopic Characterization of the Interaction of Lithium With Thin Films of the Ionic Liquid 1‐octyl‐3‐methylimidazolium Bis(trifluoromethylsulfonyl)Amide,” Physical Chemistry Chemical Physics 16 (2014): 25969–25977, 10.1039/C4CP03091E. 25356533

[67] S. Massicot , T. Sasaki , M. Lexow , et al., “Adsorption, Wetting, Growth, and Thermal Stability of the Protic Ionic Liquid Diethylmethylammonium Trifluoromethanesulfonate on Ag(111) and Au(111),” Langmuir 37 (2021): 11552–11560, 10.1021/acs.langmuir.1c01823. 34569794 PMC8495895

[68] G. te Velde , F. M. Bickelhaupt , E. J. Baerends , et al., “Chemistry with ADF,” Journal of Computational Chemistry 22 (2001): 931–967.

[69] E. van Lenthe , E. J. Baerends , and J. G. Snijders , “Relativistic Regular Two‐component Hamiltonians,” Journal of Chemical Physics 99 (1993): 4597–4610, 10.1063/1.466059.

[70] E. van Lenthe , E. J. Baerends , and J. G. Snijders , “Relativistic Total Energy Using Regular Approximations,” Journal of Chemical Physics 101 (1994): 9783–9792, 10.1063/1.467943.

[71] E. van Lenthe and E. J. Baerends , “Optimized Slater‐type Basis Sets For The Elements 1–118,” Journal of Computational Chemistry 24 (2003): 1142–1156, 10.1002/jcc.10255. 12759913

[72] C. Adamo and V. Barone , “Toward Reliable Density Functional Methods Without Adjustable Parameters: The PBE0 Model,” Journal of Chemical Physics 110 (1999): 6158–6170, 10.1063/1.478522.

[73] J. P. Perdew , K. Burke , and M. Ernzerhof , “Generalized Gradient Approximation Made Simple,” Physical Review Letter 77 (1996): 3865–3868, 10.1103/PhysRevLett.77.3865. 10062328

[74] J. P. Perdew , K. Burke , and M. Ernzerhof , “Generalized Gradient Approximation Made Simple,” Physical Review Letter 78 (1997): 1396, 10.1103/PhysRevLett.78.1396. 10062328

[75] S. Grimme , “Semiempirical GGA‐Type Density Functional Constructed With a Long‐Range Dispersion Correction,” Journal of Computational Chemistry 27 (2006): 1787–1799, 10.1002/jcc.20495. 16955487

[76] R. F. W. Bader , Atoms in Molecules: A Quantum Theory (Oxford University Press, 1990), 10.1093/oso/9780198551683.001.0001.

[77] J. I. Rodríguez , “An Efficient Method for Computing the QTAIM Topology of a Scalar Field: The Electron Density Case,” Journal of Computational Chemistry 34 (2013): 681–686, 10.1002/jcc.23180. 23175458

[78] S. K. Wolff , T. Ziegler , E. van Lenthe , and E. J. Baerends , “Density Functional Calculations of Nuclear Magnetic Shieldings Using the Zeroth‐Order Regular Approximation (ZORA) for Relativistic Effects: ZORA Nuclear Magnetic Resonance,” Journal of Chemical Physics 110 (1999): 7689–7698, 10.1063/1.478680.

[79] K. Momma and F. Izumi , “VESTA 3 for Three‐Dimensional Visualization of Crystal, Volumetric and Morphology Data,” Journal of Applied Crystallography 44 (2011): 1272–1276, 10.1107/S0021889811038970.

[80] M. Lehmann , S. N. Balogun , M. Reimann , and M. Kaupp , “The Fluoride Ion Affinity Revisited: Do We Need the Anchor‐Point Approach?,” Chemistry – A European Journal 31 (2025): e202404662, 10.1002/chem.202404662. 40028678 PMC12063057

[81] T. Yanai , D. P. TEW , and N. C. Handy , “A New Hybrid Exchange–Correlation Functional Using the Coulomb‐Attenuating Method (CAM‐B3LYP),” Chemical Physics Letters 393 (2004): 51–57, 10.1016/j.cplett.2004.06.011.

[82] A. K. Hijazi , A. Al Hmaideen , S. Syukri , et al., “Synthesis and Characterization of Acetonitrile‐Ligated Transition‐Metal Complexes With Tetrakis(pentafluorophenyl)borate as Counteranions,” European Journal of Inorganic Chemistry 2008 (2008): 2892–2898, 10.1002/ejic.200800201.

[83] A. M. Ajlouni , A. K. Hijazi , Z. A. Taha , W. Al‐Momani , A. Okour , and F. E. Kühn , “Synthesis, Characterization and Biological and Catalytic Activities of Propionitril: Ligated Transition Metal Complexes With [B(C_6_F_5_)_4_] as Counter Anion,” Catalysis Letters 149 (2019): 2317–2324, 10.1007/s10562-019-02804-9.

[84] T. Yoshida and K. Yamasaki , “The Core‐Level Binding Energies and the Structures of Nickel Complexes,” Bulletin of the Chemical Society of Japan 54 (1981): 935–936, 10.1246/bcsj.54.935.

[85] M. C. Biesinger , L. W. M. Lau , A. R. Gerson , and R. S. C. Smart , “The Role of the Auger Parameter in XPS Studies of Nickel Metal, Halides and Oxides,” Physical Chemistry Chemical Physics 14 (2012): 2434, 10.1039/c2cp22419d. 22249653

[86] M. C. Biesinger , B. P. Payne , L. W. M. Lau , A. Gerson , and R. S. C. Smart , “X‐Ray Photoelectron Spectroscopic Chemical State Quantification of Mixed Nickel Metal, Oxide and Hydroxide Systems,” Surface and Interface Analysis 41 (2009): 324–332, 10.1002/sia.3026.

[87] I. Antsiburov , R. Bühler , J. Stephan , et al., “Homoleptic Seven‐Coordinate Ti(0) and Zr(0) Through a New Stabilization Mode,” Chemical Science 17 (2026): 8989–8997, 10.1039/D6SC00226A. 41868159 PMC13001666

