# Supplementary material for: Formation of Gallium Monofluoride in the Coordination Sphere of Nickel
Source: Angew Chem Int Ed Engl. 2026 Jun 18;65(34):e1189853. doi: 10.1002/anie.1189853 (PMC13480833; doi:10.1002/anie.1189853)
Supplement: Supplementary file 1 [file ANIE-65-e1189853-s001.pdf]

Supporting Information for

Formation of Gallium Monofluoride in the Coordination Sphere of Nickel

Johannes Stephan,[a,b] *Samia Kahlal*,[c] *Jean-Yves Saillard*,*[c] *Marvin Mühlau*,[d] *Lisa Gremmel*,[a,b] *Christian Gemel*[a,b] & *Roland A. Fischer**[a,b]

[a]TUM School of Natural Sciences, Department of Chemistry, Chair of Inorganic and Metalorganic Chemistry, Technical University of Munich, Lichtenbergstraße 4, D-85748 Garching, Germany

[b]Catalysis Research Center, Technical University of Munich, Ernst-Otto-Fischer-Straße 1, D-85748 Garching, Germany

[c]Univ Rennes CNRS, ISCR-UMR 6226, F-35000 Rennes, France

[d]TUM School of Natural Sciences, Department of Chemistry, TUMint.Energy Research GmbH, Technical University of Munich, Lichtenbergstraße 4, D-85748 Garching, Germany

Correspondence to: jean-yves.saillard@univ-rennes.fr, roland.fischer@tum.de

TABLE OF CONTENTS

1. Materials and Methods 1
2. Experimental Procedures 5
[(Cp*Ga)$_4$Ni(GaF(NC^{t}Bu)$_2$)](BAr^{F24})$_2$ (**1**) 5
[(Cp*Ga)$_4$Ni(NC^{t}Bu)](BAr^{F24})$_2$ (**2**) 6
[Ni(NC^{t}Bu)$_6$](BAr^{F24})$_2$ 7
[Ni(NC^{t}Bu)$_6$](BAr^{F20})$_2$ 7
Mechanistic Considerations 8
Exchange Experiments 8
Reaction Optimisation for (**1**) 9
Fluoride Transfer Reactions 9
Gutmann-Beckett Method for Estimation of Lewis Acidity 10
3. Crystallographic Data 11
[(Cp*Ga)$_4$Ni(GaF(NC^{t}Bu)$_2$)](BAr^{F24})$_2$ (**1**) + 4 PhF 11
[(Cp*Ga)$_4$Ni(GaF(NC^{t}Bu)$_2$)](BAr^{F24})$_2$ (**1**) + 2 PhF 12
[(Cp*Ga)$_4$Ni(GaF(NC^{t}Bu)$_2$)](BAr^{F24})$_2$ (**1**) + *n*-hexane 13
[(Cp*Ga)$_4$Ni(NC^{t}Bu)](BAr^{F24})$_2$ (**2**) 14
[(Cp*Ga)$_4$Ni(GaOTf(NC^{t}Bu)$_2$)](BAr^{F24})$_2$ (**3**) 15
[(η^5-C$_5$Me$_5$)Ni(η^4-C$_5$Me$_5$H)]BAr^{F20} 17
[(Cp*)Ni(GaCp*)$_2$]BAr^{F20} 18
[(η^5-C$_5$Me$_5$)Ni(η^4-C$_5$Me$_5$H)]BAr^{F24} 19
[Ni(NC^{t}Bu)$_6$](BAr^{F20})$_2$ 20
4. X-Ray Photoelectron Spectroscopy 21
5. Computational Part 25
6. NMR & Additional Spectra 30
Compound (**1**) 30
Compound (**2**) 34
[Ni(NC^{t}Bu)$_6$](BAr^{F24})$_2$ & [Ni(NC^{t}Bu)$_6$](BAr^{F20})$_2$ 35
Mechanistic Aspects 36
Fluoride Transfer Experiments 39
7. IR Spectra 41
8. References 43

1. Materials and Methods

General

All experiments were carried out using standard Schlenk and glovebox techniques under a purified argon atmosphere. Glassware was heated with hexamethyldisilazane to yield passivated surfaces. Solvents were dried using an MBraun Solvent Purification System (SPS) and stored over activated 3 Å molecular sieves. Fluorobenzene (Sigma-Aldrich, 99%) and 1,2-difluorobenzene (ABCR Chemicals Germany, 98%) were distilled fractionally, dried by passing through a column of activated neutral alumina and stored over activated 3 Å molecular sieves in J-Young ampoules. Trimethylacetonitrile (ABCR Chemicals Germany, 98%) was distilled fractionally using a short Vigreux column, dried by passing through activated basic alumina and stored over activated 3 Å molecular sieves. Trimethylsilyl triflate (Sigma Aldrich, 98%) and benzoyl chloride were stored in argon-filled glassware and used as received. The final water content of all solvents was checked by Karl-Fischer titration and did not exceed 3 ppm. All solvents were degassed before use. Deuterated solvents were degassed and dried using activated 3 Å molecular sieves before use and stored in an argon-filled glovebox.

Chemicals were purchased from commercial suppliers, including Sigma-Aldrich and ABCR Chemicals Germany. $Ni(acac)_2$ was dried by refluxing the hydrated complex (ABCR Chemicals, Germany) in dry toluene using a *Dean-Stark* apparatus, followed by drying the resulting green residue overnight *in vacuo* at 110 °C after draining the solvent.[1] The following compounds were synthesised according to literature-known procedures:

GaCp*[2-3]

$Na(BAr^{F24})$[4-6]

$H(Et_2O)_2(BAr^{F24})$[7]

$Na(BAr^{F20})$[8-9]

$Ag(BAr^{F20})$[4]

NMR Spectroscopy

NMR spectra were recorded on either a Bruker AV 400US or AVHD 500cryo spectrometer. Spectra were referenced to the residual solvent peak as an internal standard. Deuterated solvents were stored over activated 3 Å molecular sieves and degassed before use. Spectra in non-deuterated fluorobenzene or 1,2-difluorobenzene were recorded by adding 10 vol% C_6D_6 to the solution. Chemical shifts are reported in parts per million, including the relative integral, multiplicity (s = singlet, d = doublet, q = quartet and m = multiplet, bs = broad singlet) and assignment.

Vibrational Spectroscopy

FT-IR spectra were measured on a *Bruker* Alpha FT-IR spectrometer with ATR geometry, using a diamond ATR unit in an argon-filled glovebox. The spectra were processed using the OPUS software package (version 7.5, *Bruker Optik GmbH,* 2014).

UV-Vis Spectroscopy

UV-Vis spectra were measured either on an *Agilent Technologies* Cary 60 UV-Vis spectrometer in a quartz cuvette (d = 1 cm) under argon atmosphere or in glass vials inside an argon-filled glovebox using an *Ocean Insight* FLAME-T-XR1-ES miniature spectrometer, equipped with a DH-2000-BAL UV-Vis-NIR light source and a submersible sensor with a gap width of 1 mm. Samples were prepared by diluting the respective compound in 1,2-difluorobenzene or fluorobenzene and passing the solution through a syringe filter.

Elemental Analysis

Elemental analyses were conducted at the microanalytical laboratory at the Catalysis Research Center of the Technical University of Munich, using an *Eurovector* EA3000 CHNS combustion analyser. 1-2 mg of sample was weighed in a tin boat (in a glovebox for air-sensitive samples and sealed airtight) and then dynamically spontaneously combusted at 1800 °C. Combustion gases were separated by gas chromatography, and detected with a thermal conductivity detector. The presented values are single or double determinations.

Crystallography

Data were collected on a Bruker D8 VENTURE three angle single crystal x-ray diffractometer equipped either with a Bruker PHOTON III detector, a TXS rotating anode with MoK_{α} radiation (λ = 0.71073 Å) and a Helios optic monochromator or equipped with a Bruker Photon III) detector, an IMS microsource with MoK_{α} radiation (λ = 0.71073 Å) and a Helios optic monochromator, using the APEX4 software package.[C1] Measurements were performed on single crystals coated with perfluorinated ether. The crystals were mounted on a Kapton micro sampler and frozen under a stream of cold nitrogen. A matrix scan was used to determine the initial lattice parameters. Reflections were corrected for Lorentz and polarisation effects, scan speed, and background using SAINT V8.40B.[C2] Absorption correction, including odd and even ordered spherical harmonics, was performed using SADABS.[C3] Space group assignments were based on systematic absences, E statistics, and successful structure refinement. The structures were solved using SHELXT with the aid of successive difference Fourier maps, and were refined against all data using SHELXL-2019/1 in conjunction with SHELXLE.[C4,C5,C6] Hydrogen atoms were calculated in ideal positions as follows: Methyl hydrogen atoms were refined as part of rigid rotating groups, with a C–H distance of 0.98 Å and $U_{iso(H)} = 1.5 \cdot U_{eq(C)}$. Other H atoms were placed in calculated positions and refined using a riding model, with methylene and aromatic C–H distances of 0.99 Å and

0.95 Å, respectively, and other C–H distances of 1.00 Å, all with $U_{iso(H)} = 1.2 \cdot U_{eq(C)}$. Non-hydrogen atoms were refined with anisotropic displacement parameters. Full-matrix least-squares refinements were carried out by minimizing $\Sigma\ w(F_o^2 - F_c^2)^2$ with the SHELXL weighting scheme.[C5] Neutral atom scattering factors for all atoms and anomalous dispersion corrections for the non-hydrogen atoms were taken from *International Tables for Crystallography*.[C7] A split layer refinement was used for disordered groups and additional restraints on distances, angles and anisotropic displacement parameters were employed to ensure convergence within chemically reasonable limits, if necessary. Whole-molecule disorder and rotational disorder, e.g. of CF_3 moieties, were modelled using the *DSR* tool plugin within SHELXLE.[C8] Heavily disordered solvent molecules were treated as a diffuse contribution to the overall scattering without specific atom positions using the PLATON/SQUEEZE procedure.[C9] Images of the crystal structures were created with Mercury (main article).[C10] CIF files and crystallographic tables were generated using FinalCif.[C11]

X-Ray Photoelectron Spectroscopy

The X-ray photoelectron spectroscopy spectra were measured on an Axis Supra (Kratos Analytical, UK) X-ray photoelectron spectrometer. The reference substances, as well as the target compound $[(Cp^*Ga)_4Ni(GaF(NC^tBu)_2)](BAr^{F24})_2$ (**1**) were dried *in vacuo* overnight and afterwards mounted on a stainless steel stub inside an argon-filled glove box. The sample holder was attached inside the inert transfer device, which was then flanged to the XPS antechamber (flexi lock). The samples were kept in the antechamber until a pressure of $\sim 1 \times 10^{-7}$ Torr was reached, and then transferred to the sample analysis chamber, where the pressure was kept below $\sim 2 \times 10^{-8}$ Torr throughout the measurement period. The excitation energy was provided by a monochromatic Al K_α source (1486.6 eV), with an emission current of 15 mA and a spot size of approximately 700 µm × 300 µm. The measurement procedure consisted of recording several different scans (the respective beam energy is given in binding energy): 1) 3 scans of the Ni 2p region from 895-840 eV and the Ga 2p region from 1170-1100 eV, with a 0.2 eV step size and a 100 ms dwell time. 2) 1 wide scan from 1200 to −5 eV, with a 1.0 eV step size and a 100 ms dwell time. 3) 5 scans of the following regions Ni 2p (from 895-800 eV, with a 0.1 eV step size and 300 ms dwell time), Ga 2p (from 1170-1100 eV, with a 0.1 eV step size and 300 ms dwell time), F 1s (from 700-670 eV, with a 0.1 eV step size and 300 ms dwell time), O 1s (from 535-520 eV, with a 0.1 eV step size and 300 ms dwell time), N 1s (from 420-380 eV, with a 0.1 eV step size and 300 ms dwell time), C 1s (from 305-272 eV, with a 0.1 eV step size and 300 ms dwell time), B 1s (from 210-170 eV, with a 0.1 eV step size and 300 ms dwell time), Ni 3p (from 90-45 eV, with a 0.1 eV step size and 300 ms dwell time), Ga 3d (from 30-10 eV, with a 0.1 eV step size and 300 ms dwell time) and 4) 3 scans analogously to step 1). Thereafter, the X-ray source was switched to an Ag K_α source (2984.3 eV), with an emission current of 15 mA and a spot size of roughly 700 µm × 300 µm. The measurement procedure consisted of recording several different scans (the respective beam energy is given in binding energy): 1) 3 scans of the Ni 2p region from 895-840 eV and the Ga 2p region from 1170-1100 eV, with a 0.5 eV step size and a 500 ms dwell time. 2) 1 wide scan from 2500- −5 eV, with a 1.0 eV step size and a 200 ms dwell time. 3) 10 scans of the following regions Ni 2p (from 885-835 eV, with a 0.2 eV step size and 1000 ms dwell time), Ga 2p (from 1160-1100 eV, with a 0.2 eV step size and 1000 ms

dwell time), F 1s (from 695-670 eV, with a 0.2 eV step size and 1000 ms dwell time), O 1s (from 535-520 eV, with a 0.2 eV step size and 1000 ms dwell time), N 1s (from 420-380 eV, with a 0.2 eV step size and 1000 ms dwell time), C 1s (from 300-272 eV, with a 0.2 eV step size and 1000 ms dwell time), B 1s (from 210-170 eV, with a 0.2 eV step size and 1000 ms dwell time), Ni 3p (from 90-45 eV, with a 0.2 eV step size and 1000 ms dwell time), Ga 3d (from 25-10 eV, with a 0.2 eV step size and 1000 ms dwell time) and 4) 3 scans analogously to step 1). For the binding energy correction, the F 1s of the samples was referenced to the CF_3-groups arising from the two BAr^{F24} anions (688.5 eV).[10-11] For the detail-scan spectra, a Shirley background was subtracted. Following, a min-max normalisation was performed. Then the spectra were fitted with a superposition of a Gaussian (70%) and Lorentzian (30%) shape function (GL30). For the Ni 2p spectra and Ga 2p spectra, the Ni $2p_{3/2}$ and Ni $2p_{1/2}$ (and Ga $2p_{3/2}$ and Ga $2p_{1/2}$, respectively) were fitted in a constrained manner with a difference in binding energy of 17.3 eV for Ni and 26.9 eV for Ga, and the $2p_{1/2}$ having half the peak area of the $2p_{3/2}$.

Computational Details

Density Functional Theory (DFT) calculations were performed using the Amsterdam Density Functional code (ADF2020.101)[12] with the ZORA Hamiltonian[13-14] for the incorporation of scalar relativistic corrections. The triple-ξ Slater basis set plus two polarisation functions (STO-TZ2P)[15] and the PBE0 hybrid functional[16-18] were used for all calculations, with the addition of Grimme's D3 empirical corrections[19] to account for dispersion effects. Frequency calculations were performed for all the optimised geometries to confirm that these are true energy minima on the potential energy surface. Natural atomic orbital (NAO) populations and charges, as well as Wiberg bond indices, were computed using the natural bond orbital NBO6.0 program[20] implemented in the ADF2020.101 package. Quantum theory of atoms in molecules (QTAIM)[21-23] analysis was performed as implemented in the ADF2020.101 package. Energy decomposition analysis (EDA) calculations were performed in accordance with the Morokuma-Ziegler formalism.[24-26] The NMR chemical shifts were computed according to the gauge-independent atomic orbitals (GIAO) method.[27] Depictions of optimised structures were generated using VESTA3.[28]

Calculations of the fluoride ion affinity (FIA) of $[(Cp^*Ga)_4Ni(NC^tBu)]^{2+}$ were performed according to the direct method recently proposed by Kaupp and coworkers,[29] as the negative of the enthalpy of the reaction of a Lewis acid with a fluoride ion in the gas phase. In order to save computational efforts, the geometries of $[(Cp^*Ga)_4Ni(NC^tBu)]^{2+}$ and its fluoride adduct were fully optimized at the same TZ2P/PBE0-D3 level as described above. Then, single-point calculations were performed on these structures (and F^-) with the large basis set ET/ET-QZ3P-1DIFFUSE[15] and the CAM-B3LYP functional,[30] to which the Grimme's D3 empirical corrections were added.[19] The choice of the CAM-B3LYP functional was justified for it provides reasonably good FIA values at a relatively cheap computational cost.[29] The FIA was then calculated approximating total energies with enthalpies. The accuracy of our approach was tested by comparison with $[SiMe_3]^+$, whose FIA, computed at the same level, was found to be 920 kJ/mol, the latter to be compared to the reported high-accuracy benchmark value of 954 kJ/mol reported by Kaupp and coworkers.[29]

2. Experimental Procedures

$[(Cp^*Ga)_4Ni(GaF(NC^tBu)_2)](BAr^{F24})_2$ (1)

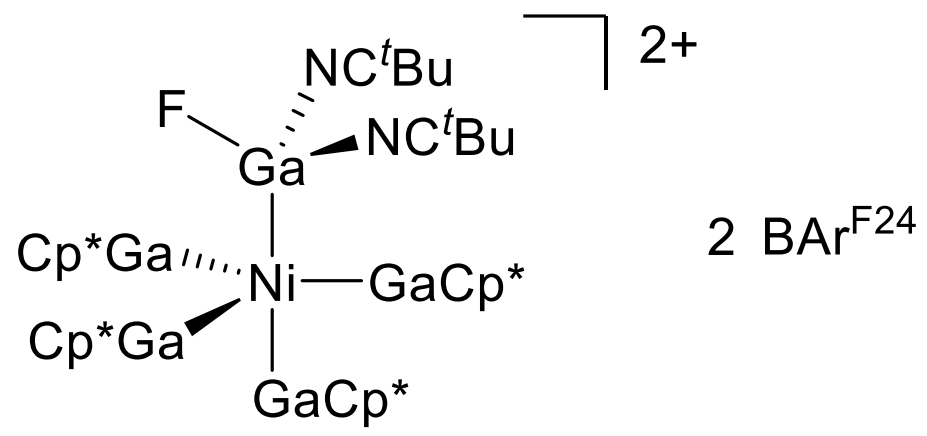

Optimised procedure (elevated temperature)

Inside a glovebox, a small Schlenk tube with a Teflon-coated stir bar is charged with $[Ni(NC^tBu)_6](BAr^{F24})_2$ (0.0402 g, 10 µmol, 1.00 eq.). Dry fluorobenzene (1 mL) and GaCp* (0.0182 g, 90 µmol, 5.22 eq.) as a solution in fluorobenzene (1 mL) are added. Instantaneously, a dark violet to blackish precipitate forms. Heating the suspension to 35 °C for four days while stirring results in a homogeneous, bright orange solution, to which *n*-hexane (6 mL) is added to form a dark-orange oil. Storing at –32 °C overnight results in solidification of the oily residue, and the formation of small single crystals of $[(Cp^*Ga)_4Ni(GaF(NC^tBu)_2)](BAr^{F24})_2$ (**1**) (0.00261 g, 9 µmol, 53%). **1** may exist as two solvatomorphs depending on the mode of crystallisation (*P*-1, four fluorobenzene molecules; $P2_1/n$, two fluorobenzene molecules). Note that heating the reaction mixture to 45 °C or higher does not yield **1**, but nickelocenes as the main product.

^{1}H NMR (500 MHz, 300 K, 1,2-difluorobenzene/10% $[D_6]$benzene, ppm): δ = 8.17 – 8.14 (m, 16H, o-H_{Ar}), 7.50 (s, 8H, p-H_{Ar}), 1.86 (s, 60H, C_5Me_5), 1.50 (s, 18H, $NCCMe_3$).

^{11}B NMR (128 MHz, 298 K, 1,2-difluorobenzene/10% $[D_6]$benzene, ppm): δ = –6.15 ($B(Ar^F)_4$).

^{13}C NMR (126 MHz, 300 K, 1,2-difluorobenzene/10% $[D_6]$benzene, ppm): δ = 163.4 (d, $J_{C\text{-}F}$ = 245.0 Hz, PhF), 162.6 (q, $J_{B\text{-}C}$ = 49.7 Hz, B-*C*), 151.0 (d, $J_{C\text{-}F}$ = 51.2 Hz, N*C*CMe$_3$), 135.4 (Ar^F), 130.2 (d, $J_{C\text{-}F}$ = 8.0 Hz, PhF), 130.0 (qdd, $J_{C\text{-}F}$ = 31.6 5.8 2.8 Hz, Ar^F), 125.2 (q, $J_{C\text{-}F}$ = 272.0 Hz, Ar^F), 124.3 (d, $J_{C\text{-}F}$ = 3.0 Hz, PhF), 117.9 (p, $J_{C\text{-}F}$ = 4.3 Hz, Ar^F), 116.3 (C_5Me_5), 115.4 (d, $J_{C\text{-}F}$ = 21.1 Hz, PhF), 29.4 ($NCCMe_3$), 26.8 ($NCCMe_3$), 9.6 (C_5Me_5).

^{19}F NMR (471 MHz, 300 K, 1,2-difluorobenzene/10% $[D_6]$benzene, ppm): δ = –62.99 (s, 48F, CF_3), –79.91 (bs, 1F, Ga*F*), –113.84 (s, 2F, PhF, co-crystallized).

^{71}Ga NMR (122 MHz, 295 K, 1,2-difluorobenzene/10% $[D_6]$benzene, ppm): *not detected*.

IR (ATR, cm^{-1}): $\tilde{\nu}$ = 2991 (w), 2926 (w), 2872 (w), 2265 (w, CN), 1610 (w), 1596 (w), 1354 (s), 1275 (s), 1114 (s), 888 (m), 806 (m), 757 (w), 745 (m), 715 (m), 682 (m), 669 (m), 609 (w, Ga–F), 589 (w), 519 (w), 500 (w), 447 (w).

Elemental analysis calcd (%) for $NiGa_5C_{114}H_{102}B_2F_{49}N_2$ + 2 C_6H_5F: C: 49.58, H: 3.70, N: 0.92, B: 0.71, F: 31.75, Ni: 1.92, Ga: 11.42; found: C: 48.96, H: 3.79, N: 1.03, F: 32.10.

UV-Vis (1,2-difluorobenzene): λ (nm) = 411, 482.

Initial procedure (room temperature)

Inside a glovebox, $[Ni(NC^{t}Bu)_6](BAr^{F24})_2$ (0.0402 g, 10 µmol, 1.00 eq.) is suspended in dry fluorobenzene (1 mL) inside a small Schlenk tube, and GaCp* (0.0205 g, 100 µmol, 5.71 eq.) is added as a solution in fluorobenzene (1 mL). Instantaneously, a dark violet to blackish precipitate forms. After storing at room temperature for three months without agitation, one large block-shaped, red single crystal of $[(Cp^*Ga)_4Ni(GaF(NC^{t}Bu)_2)](BAr^{F24})_2$ (0.0203 g, 7 µmol, 41%) is formed. Either cooling the supernatant orange solution to –32 °C for three days or layering with *n*-hexane (6 mL) at room temperature yields another crop of smaller-sized single crystals of **1** (0.0051 g, 2 µmol, 10%). Altogether, **1** is obtained reproducibly in a total yield of 0.0254 g (9 µmol, 51%). Note that crystalline $[(Cp^*Ga)_4Ni(GaF(NC^{t}Bu)_2)](BAr^{F24})_2$ (**1**) may exist as two solvatomorphs (*P*-1, four fluorobenzene molecules; $P2_1/n$, two fluorobenzene molecules). The spectral properties were the same as for **1** obtained at slightly higher temperatures of 35 °C. **1** is also obtained when the recation is conducted in dry chlorobenzene as the solvent, but with traces of protonated decamethylnickelocene as the side product.

$[(Cp^*Ga)_4Ni(NC^{t}Bu)](BAr^{F24})_2$ (2)

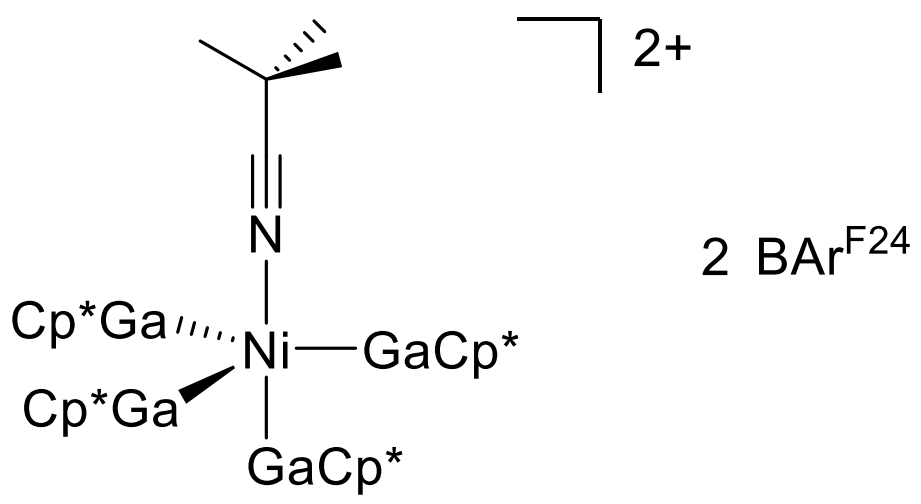

Inside a glovebox, $[Ni(NC^{t}Bu)_6](BAr^{F24})_2$ (0.1008 g, 44 µmol, 1.00 eq.) is suspended in dry fluorobenzene (2 mL), and GaCp* (0.0415 g, 220 µmol, 4.99 eq.) is added as a solution in fluorobenzene (1 mL). Instantaneously, a dark violet to blackish precipitate forms. The suspension is stirred at room temperature for 30 minutes after which the solids are allowed to settle and *n*-hexane (8 mL) is added to ensure complete precipitation. The product is isolated *via* cannula filtration, washed with *n*-hexane (2 mL) and dried *in vacuo*. $[(Cp^*Ga)_4Ni(NC^{t}Bu)](BAr^{F24})_2$ (**2**) is obtained as a dark purple microcrystalline solid (0.0756 g, 28 µmol, 64%). **2** is stable as a dry solid under an inert atmosphere but decomposes rapidly in solution.

^{1}H NMR (400 MHz, 298 K, CD_2Cl_2, ppm): δ = 7.72 (s, 16H, *o*-H_{Ar}), 7.56 (s, 8H, *p*-H_{Ar}), 2.04 (s, 60H, C_5Me_5), 1.45 (bs, 18H, $NCCMe_3$).

IR (ATR, cm^{-1}): $\tilde{\nu}$ = 2991 (w), 2283 (w, CN), 1610 (w), 1354 (s), 1276 (s), 1161 (s), 1114 (s), 1091 (s), 886 (m), 753 (w), 744 (w), 715 (m), 682 (m), 669 (m), 586 (w), 447 (w).

Elemental analysis calcd (%) for $NiGa_4C_{109}H_{90}B_2F_{48}N$ + C_6H_5F: C: 49.67, H: 3.44, N: 0.50, B: 0.78, F: 33.47, Ni: 2.11, Ga: 10.03; found: C: 449.30, H: 3.53, N: 1.43.

[Ni(NC^{t}Bu)$_6$](BAr^{F24})$_2$

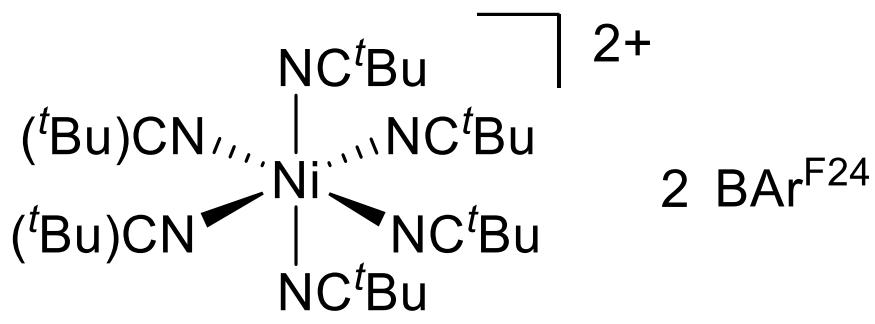

The synthesis procedure for [Ni(NC^{t}Bu)$_6$](BAr^{F24})$_2$ is based on a literature-known protocol for preparing [Ni(MeCN)$_6$](BAr^{F24})$_2$, [Ni(MeCN)$_6$](BF$_4$)$_2$ and [Ni(MeCN)$_6$](SO$_3$CF$_3$)$_2$.[17] Anhydrous bis(acetylacetonato)-nickel(II) (0.1901 g, 0.740 mmol, 1.00 eq.) and H(Et$_2$O)$_2$BAr^{F24} (1.500 g, 1.48 mmol, 2.00 eq.) are weighed into a Schlenk tube and dry trimethylacetonitrile (4 mL) is added. The resulting blue solution is stirred at room temperature overnight. The solution is reduced *in vacuo* to approximately 3 mL and dry *n*-hexane (10 mL) is added to precipitate the product. After cannula filtration, washing with *n*-hexane (2 mL) and drying *in vacuo,* [Ni(NC^{t}Bu)$_6$](BAr^{F24})$_2$ (1.360 g, 0.60 mol, 80%) is obtained as a pale blue powder.

^{1}H NMR (400 MHz, 298 K, 1,2-difluorobenzene/10% [D$_6$]benzene, ppm): δ = 8.15 (m, 16H, *ortho*-H), 7.51 (s, 8H, *para*-H), 1.31 (bs, NC(C*H*$_3$)$_3$).

IR (ATR, cm^{-1}): $\tilde{\nu}$ = 2987 (w), 2282 (w, CN), 1610 (w), 1354 (s), 1277 (s), 1118 (s), 886 (m), 838 (m), 715 (m), 683 (m), 669 (m).

Elemental analysis calcd (%) for NiC$_{94}$H$_{78}$B$_2$F$_{48}$N$_6$: C: 49.43, H: 3.44, N: 3.68, B: 0.96, F: 39.93, Ni: 2.57; found: C: 49.81, H: 3.54, N: 3.60.

[Ni(NC^{t}Bu)$_6$](BAr^{F20})$_2$

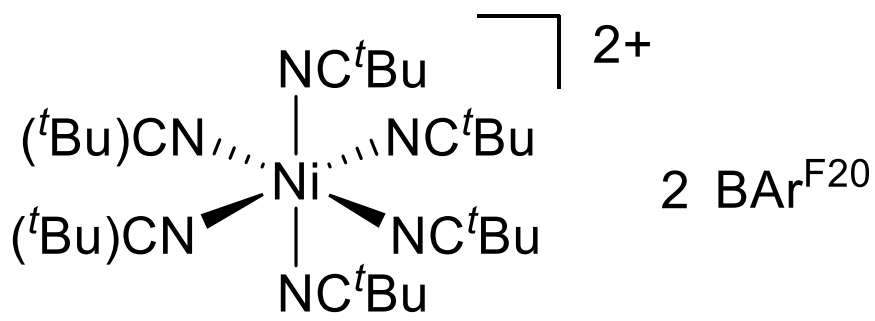

The synthesis procedure for [Ni(NC^{t}Bu)$_6$](BAr^{F20})$_2$ is based on a literature-known protocol for preparing [Ni(MeCN)$_6$](BAr^{F20})$_2$[4, 31] and [Ni(EtCN)$_6$](BAr^{F20})$_2$.[32] Anhydrous nickel(II) bromide (0.1001 g, 389 µmol, 1.00 eq.) and Ag(BAr^{F20}) (0.7892 g, 778 µmol, 2.00 eq.) are weighed into a Schlenk tube, and acetonitrile (8 mL) is added. The resulting blue solution is stirred at room temperature overnight. After reducing the solution *in vacuo* to approximately 3 mL and storing it at –32 °C overnight, violet crystals of [Ni(NC^{t}Bu)$_6$](BAr^{F20})$_2$ (0.6985 g, 344 µmol, 88%) are obtained after cannula filtration and drying *in vacuo*.

^{1}H NMR (400 MHz, 298 K, 1,2-difluorobenzene/10% [D$_6$]benzene, ppm): δ = 1.29 (bs, NC(C*H*$_3$)$_3$).

IR (ATR, cm^{-1}): $\tilde{\nu}$ = 2964 (w), 2284 (w, CN), 1643 (w), 1512 (m), 1459 (s), 1374 (m), 1274 (m), 1239 (m), 1209 (m), 1084 (s), 975 (s), 773 (m), 756 (m), 699 (m), 684 (m), 585 (w).

Elemental analysis calcd (%) for $NiC_{70}H_{54}B_2F_{40}N_6$: C: 48.91, H: 2.84, N: 4.39, B: 1.13, F: 39.67, Ni: 3.06; found: C: 48.47, H: 2.62, N: 4.20.

Mechanistic Considerations

In an attempt to speed up the reaction by dissolving **2** completely in the much more polar solvent 1,2-difluorobenzene instead of suspending in fluorobenzene, we could only isolate the protonated decamethylnickelocene species [(η^5-C_5Me_5)Ni(η^4-C_5Me_5H)]BAr^{F24} (see Figure S1) as a product of Cp* transmetalation. This indicates that the formation of a tight ion pair between the cation in [(Cp*Ga)$_4$Ni(NC^{t}Bu)](BAr^{F24})$_2$ (**2**) and the anion BAr^{F24} might play a crucial role for C–F bond activation, as the reaction does not occur in a completely homogeneous and hence ion-separated solution.

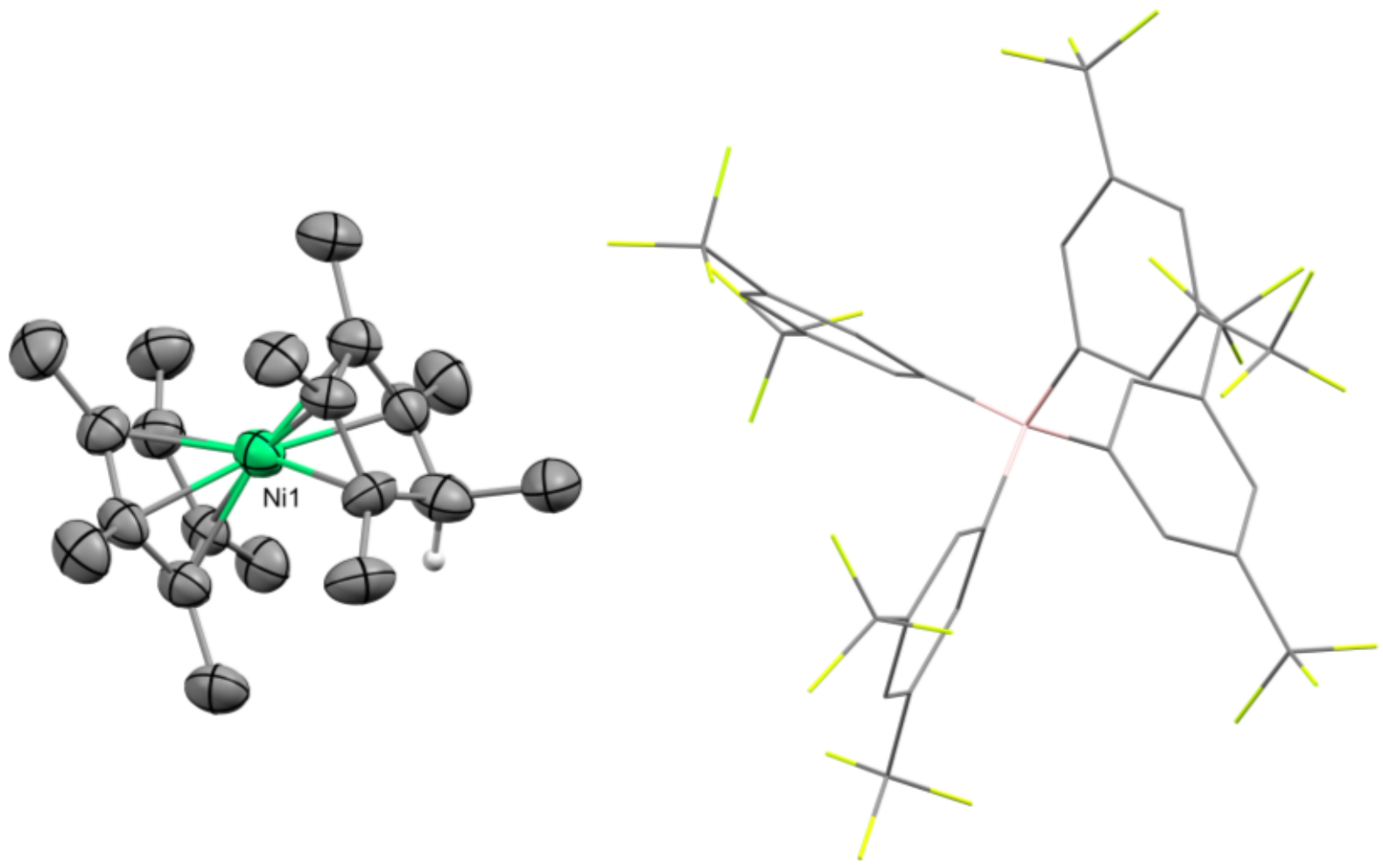

Figure S1. Molecular structure of [(η^5-C_5Me_5)Ni(η^4-C_5Me_5H)]BAr^{F24} as determined by SC-XRD. Ellipsoids are drawn at 30% probability. For clarity, only one of the two crystallographically independent nickelocene moieties is shown, and the anion is drawn in wireframe representation.

Exchange Experiments

To determine the fluorine source for the formation of **1**, exchange experiments involving both the fluorinated weakly coordinating anion and the arene solvent were performed. For that, the exclusively C(sp^3)–F bond containing BAr^{F24} anion and the C(sp^2)–F bonded BAr^{F20} anion were used in combination with both fluorobenzene (C(sp^2)–F) and trifluorotoluene (C(sp^3)–F) as solvents. Altogether, this results in four combinations, including one “all-C(sp^2)–F” and one “all-C(sp^3)–F” case, as well as two mixed hybridisation cases (see Table S1). 40 mg of the corresponding nickel trimethylacetonitrile complex with either BAr^{F24} or BAr^{F20} as the weakly coordinating anion (10 µmol, 1.00 eq.) are suspended in dry fluorobenzene or trifluorotoluene (1 mL) in a Schlenk tube. GaCp* (0.0205 g, 100 µmol, 5.71 eq.) is added as a solution in dry fluorobenzene or trifluorotoluene (1 mL), which instantaneously produces a dark violet to blackish precipitate with an orange supernatant solution. The resulting four suspensions are stored at room temperature without stirring for three months, after which orange homogeneous solutions form. The products of each reaction were analysed by SC-XRD after crystallisation by layering each solution with dry *n*-hexane (4 mL) at room temperature. **1** forms exclusively with BAr^{F24} as the

anion regardless of the solvent. When BAr^{F20} is used as an anion, no formation of **1** is observed in both fluorobenzene and trifluorotoluene. The only products that could be isolated are protonated decamethylnickelocene and $[Cp^*Ni(GaCp^*)_2]BAr^{F24}$. **1** also forms with dry chlorobenzene as the solvent when BAr^{F24} is used as the anion under the conditions described above. This strongly supports the weakly coordinating BAr^{F24} anion as the fluorine source, since no other fluorine-containing reagent than the BAr^{F24} anion is present under these conditions.

Table S1. Studies on the formation of **1,** depending on the anion and the reaction solvent. The identity of the products was confirmed by SC-XRD.

	BAr^{F24}	BAr^{F20}
PhF	**1**	$[Cp^*Ni(Cp^*H)]^+$
$PhCF_3$	**1** + $[Cp^*Ni(Cp^*H)]^+$	$[Cp^*Ni(Cp^*H)]^+$ + $[Cp^*Ni(GaCp^*)_2]^+$

Reaction Optimisation for (1)

To accelerate the formation of 1 from 3 months at room temperature, we investigated the effects of reaction temperature and solvent. For probing solvent effects, $[Ni(NC^tBu)_6](BAr^{F24})_2$ (0.0404 g, 10 µmol, 1.00 eq.) was suspended in dry chlorobenzene or toluene (1 mL each) in a Schlenk tube. GaCp* (0.0198 g, 94 µmol, 5.58 eq.) was added as a solution in either chlorobenzene or toluene (1 mL). For chlorobenzene, the dark violet to blackish precipitate of **2** was observed after half an hour, whereas for toluene, a violet hue was visible after one week. Reaction with chlorobenzene was completed at room temperature after three months of storage without stirring, after which an orange, homogeneous solution formed. **1**, accompanied by traces of protonated decamethylnickelocene, was identified by SC-XRD after crystallisation by layering with dry *n*-hexane (4 mL) at room temperature. For toluene, virtually no conversion was detected after 6 months, as indicated by a violet precipitate in a pale-yellow supernatant solution of GaCp*. Therefore, fluorobenzene was deemed the most suitable reaction medium.

In the next step, we investigated the effect of temperature on the formation of **1** in fluorobenzene as the solvent. The same scale was used as described in the paragraph above. Heating the suspension of **2** in fluorobenzene to 50 °C produces a light green homogeneous solution, which was layered with dry *n*-hexane (4 mL) at room temperature. Green crystals formed, which were identified as $[Cp^*Ni(Cp^*H)](BAr^{F24})$ by SC-XRD. Increasing the reaction temperature by only 5 °C, however, reduced the reaction time from approximately 3 months to 6 weeks with clean formation of **1**. Heating to 35 °C results in complete conversion within 4 days. At 45 °C, however, no signs of **1** could be detected, although the reaction mixture turns bright orange after 24 hours. Therefore, 35 °C has proven to be the optimum regarding reaction temperature.

Fluoride Transfer Reactions

10 mg of **1** (3.5 µmol, 1.0 eq.) are dissolved in a J-Young NMR tube in dry 1,2-difluorobenzene (0.5 mL) containing 10 wt% C_6D_6. Excess trimethylsilyl triflate (5 µL, 6 mg, 28 µmol, 7.8 eq.) or benzoyl chloride

(5 µL, 5 mg, 17 µmol, 5.0 eq.) is added to the solution using a microliter syringe. Yields were determined after 15 minutes at room temperature by ^{19}F NMR spectroscopy, using the CF_3 groups of the BAr^{F24} anions as an internal standard.

Gutmann-Beckett Method for Estimation of Lewis Acidity

The Lewis acidity of $[(Cp^*Ga)_4Ni(NC^tBu)](BAr^{F24})_2$ (**2**) was determined according to the Gutmann-Beckett method using triethylphosphine oxide (TEPO).[33-34] A stock solution of substoichiometric amounts of TEPO (5 mg/mL) in 1,2-difluorobenzene (120 µL, 0.6 mg, 0.79 eq.) was added to a solution of **2** (15 mg, 3 µmol, 1.00 eq.) in 1,2-difluorobenzene. Due to thermal decomposition of **2**, the ^{31}P NMR (see Figure S19) shows multiple peaks, and the acceptor number was calculated as a range with the peak at 85.37 ppm as the most shifted and 81.52 ppm as the least shifted peak according to the following equation: $AN = 2.21 \times (\delta(ppm) - 41.00)$.

3. Crystallographic Data

[(Cp*Ga)$_4$Ni(GaF(NC^{t}Bu)$_2$)](BAr^{F24})$_2$ (1) + 4 PhF

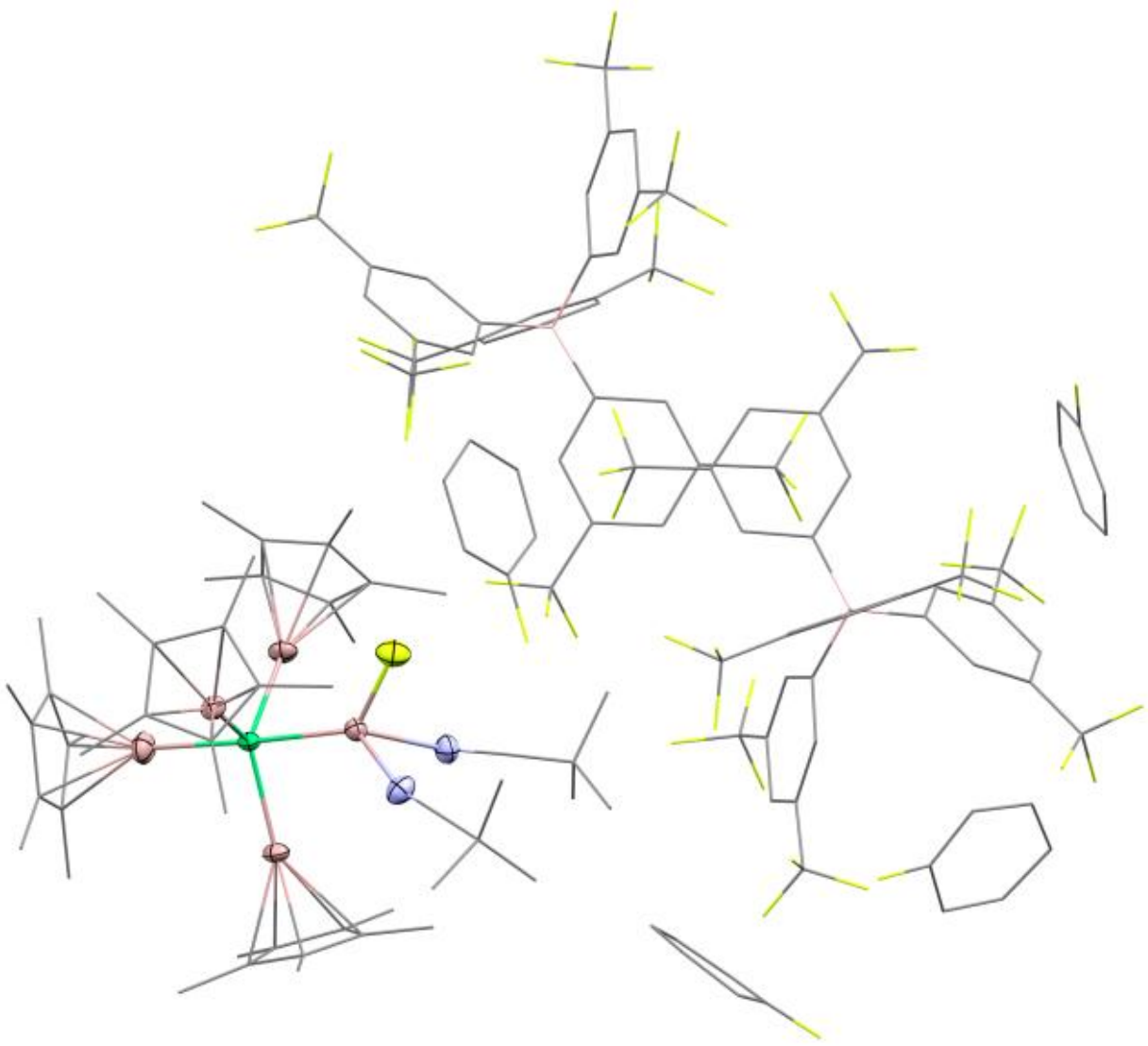

CCDC number	2500330
Empirical formula	$C_{135}H_{119.50}B_2F_{52.50}Ga_5N_2Ni$
Formula weight	3196.25
Temperature [K]	100(2)
Crystal system	triclinic
Space group (number)	$P\overline{1}$ (2)
a [Å]	19.3453(11)
b [Å]	19.6912(11)
c [Å]	20.8444(12)
α [°]	84.867(2)
β [°]	83.158(2)
γ [°]	61.496(2)
Volume [Å^3]	6923.3(7)
Z	2
ρ_{calc} [gcm^{-3}]	1.533
μ [mm^{-1}]	1.212
F(000)	3218
Crystal size [mm^3]	0.248×0.429×0.430
Crystal colour	orange
Crystal shape	block
Radiation	MoK_α (λ=0.71073 Å)
2θ range [°]	4.24 to 50.05 (0.84 Å)
Index ranges	−23 ≤ h ≤ 23<br>−23 ≤ k ≤ 23<br>−24 ≤ l ≤ 24
Reflections collected	133284
Independent reflections	24384<br>R_{int} = 0.0463<br>R_{sigma} = 0.0391
Completeness to θ = 25.026°	99.7
Data / Restraints / Parameters	24384 / 3045 / 2349
Goodness-of-fit on F^2	1.038
Final *R* indexes [I≥2σ(I)]	R_1 = 0.0344<br>wR_2 = 0.0877
Final *R* indexes [all data]	R_1 = 0.0405<br>wR_2 = 0.0918
Largest peak/hole [eÅ^{-3}]	0.94/−0.65

[(Cp*Ga)$_4$Ni(GaF(NC^{t}Bu)$_2$)](BAr^{F24})$_2$ (1) + 2 PhF

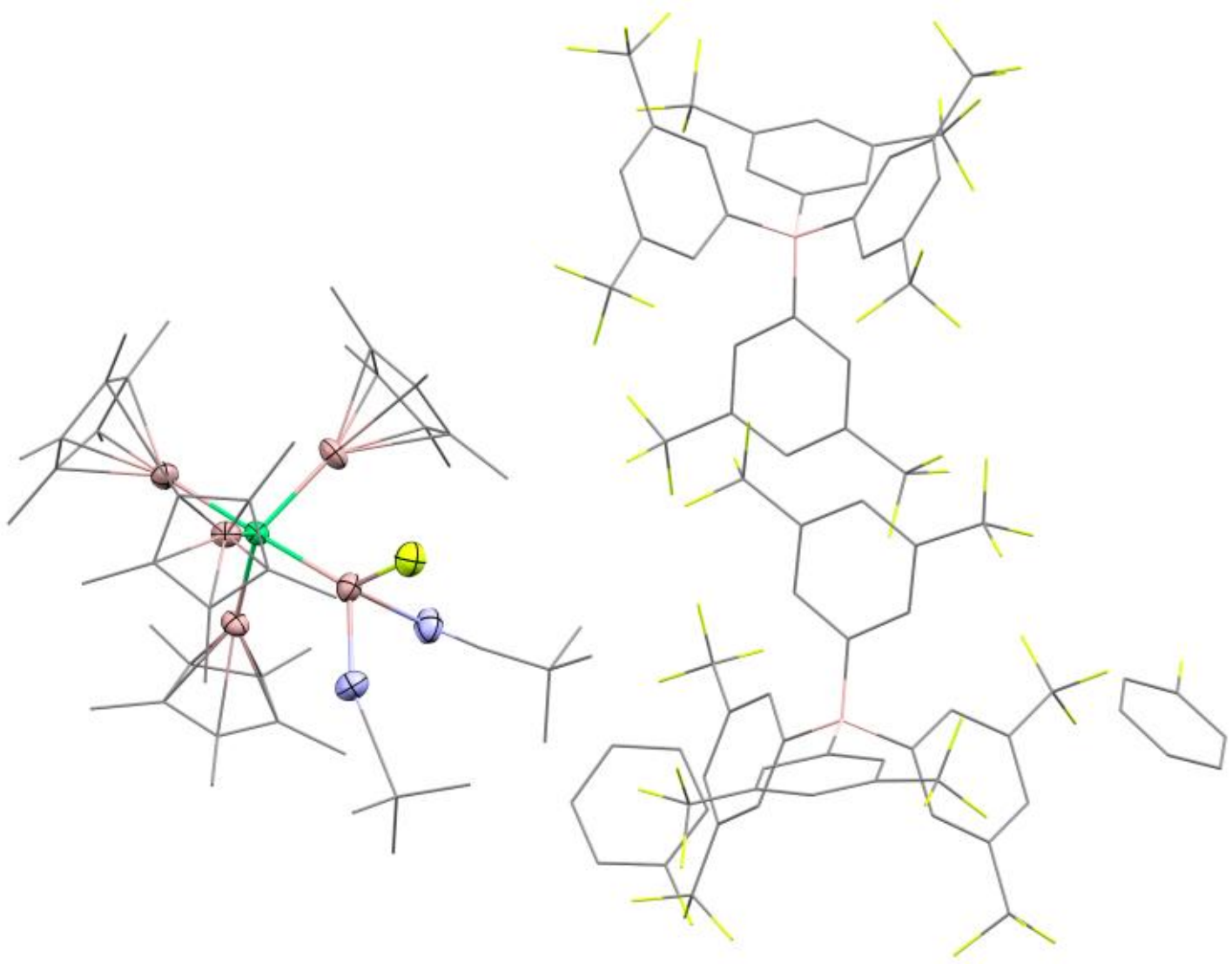

CCDC number	2500331
Empirical formula	$C_{126}H_{112}B_2F_{51}Ga_5N_2Ni$
Formula weight	3052.10
Temperature [K]	100(2)
Crystal system	monoclinic
Space group (number)	$P2_1/n$ (14)
a [Å]	19.0295(14)
b [Å]	27.5864(19)
c [Å]	25.0055(18)
α [°]	90
β [°]	95.796(3)
γ [°]	90
Volume [Å^3]	13059.7(16)
Z	4
ρ_{calc} [gcm^{-3}]	1.552
μ [mm^{-1}]	1.279
F(000)	6136
Crystal size [mm^3]	0.094×0.124×0.241
Crystal colour	orange
Crystal shape	fragment
Radiation	MoK_α (λ=0.71073 Å)
2θ range [°]	3.65 to 50.05 (0.84 Å)
Index ranges	$-22 \le h \le 22$<br>$-32 \le k \le 29$<br>$-29 \le l \le 29$
Reflections collected	324032
Independent reflections	23043<br>R_{int} = 0.0633<br>R_{sigma} = 0.0292
Completeness to θ = 25.027°	99.9
Data / Restraints / Parameters	23043 / 2514 / 2109
Goodness-of-fit on F^2	1.068
Final *R* indexes [$I \ge 2\sigma(I)$]	R_1 = 0.0471<br>wR_2 = 0.1120
Final *R* indexes [all data]	R_1 = 0.0575<br>wR_2 = 0.1183
Largest peak/hole [eÅ^{-3}]	1.24/−0.74

[(Cp*Ga)$_4$Ni(GaF(NC^{t}Bu)$_2$)](BAr^{F24})$_2$ (1) + *n*-hexane

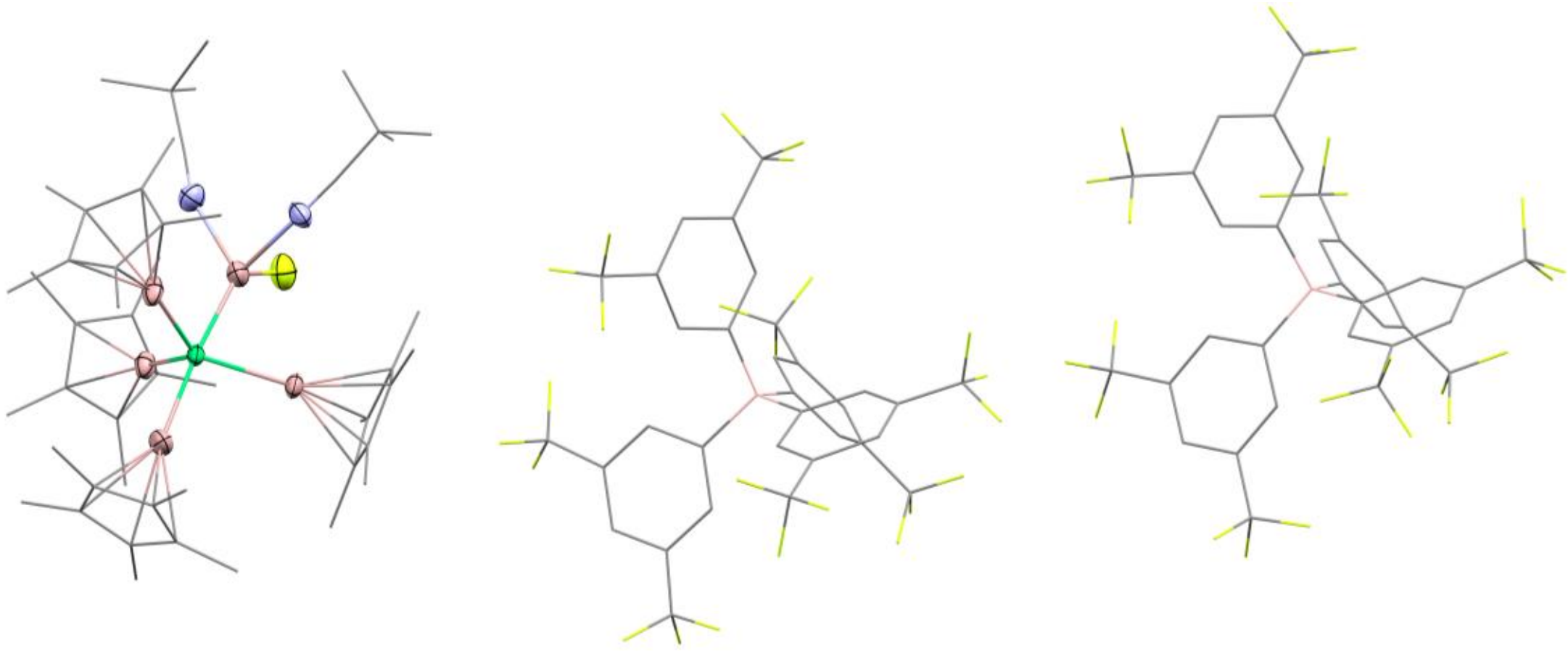

CCDC number	2500332
Empirical formula	$C_{114}H_{102}B_2F_{49}Ga_5N_2Ni$
Formula weight	2859.90
Temperature [K]	100(2)
Crystal system	triclinic
Space group (number)	$P\overline{1}$ (2)
a [Å]	15.8461(12)
b [Å]	20.1097(16)
c [Å]	20.8289(18)
α [°]	93.129(3)
β [°]	101.048(3)
γ [°]	107.348(3)
Volume [Å^3]	6173.6(9)
Z	2
ρ_{calc} [gcm^{-3}]	1.538
μ [mm^{-1}]	1.345
F(000)	2868
Crystal size [mm^3]	0.073×0.096×0.157
Crystal colour	orange
Crystal shape	fragment
Radiation	MoK_α (λ=0.71073 Å)
2θ range [°]	3.73 to 50.05 (0.84 Å)
Index ranges	−18 ≤ h ≤ 18<br>−23 ≤ k ≤ 23<br>−24 ≤ l ≤ 24
Reflections collected	263800
Independent reflections	21780<br>R_{int} = 0.0994<br>R_{sigma} = 0.0509
Completeness to θ = 25.027°	99.9
Data / Restraints / Parameters	21780 / 5632 / 2585
Goodness-of-fit on F^2	1.035
Final *R* indexes [$I \geq 2\sigma(I)$]	R_1 = 0.0873<br>wR_2 = 0.2497
Final *R* indexes [all data]	R_1 = 0.1177<br>wR_2 = 0.2864
Largest peak/hole [eÅ^{-3}]	2.98/−1.89

[(Cp*Ga)$_4$Ni(NC^{t}Bu)](BAr^{F24})$_2$ (2)

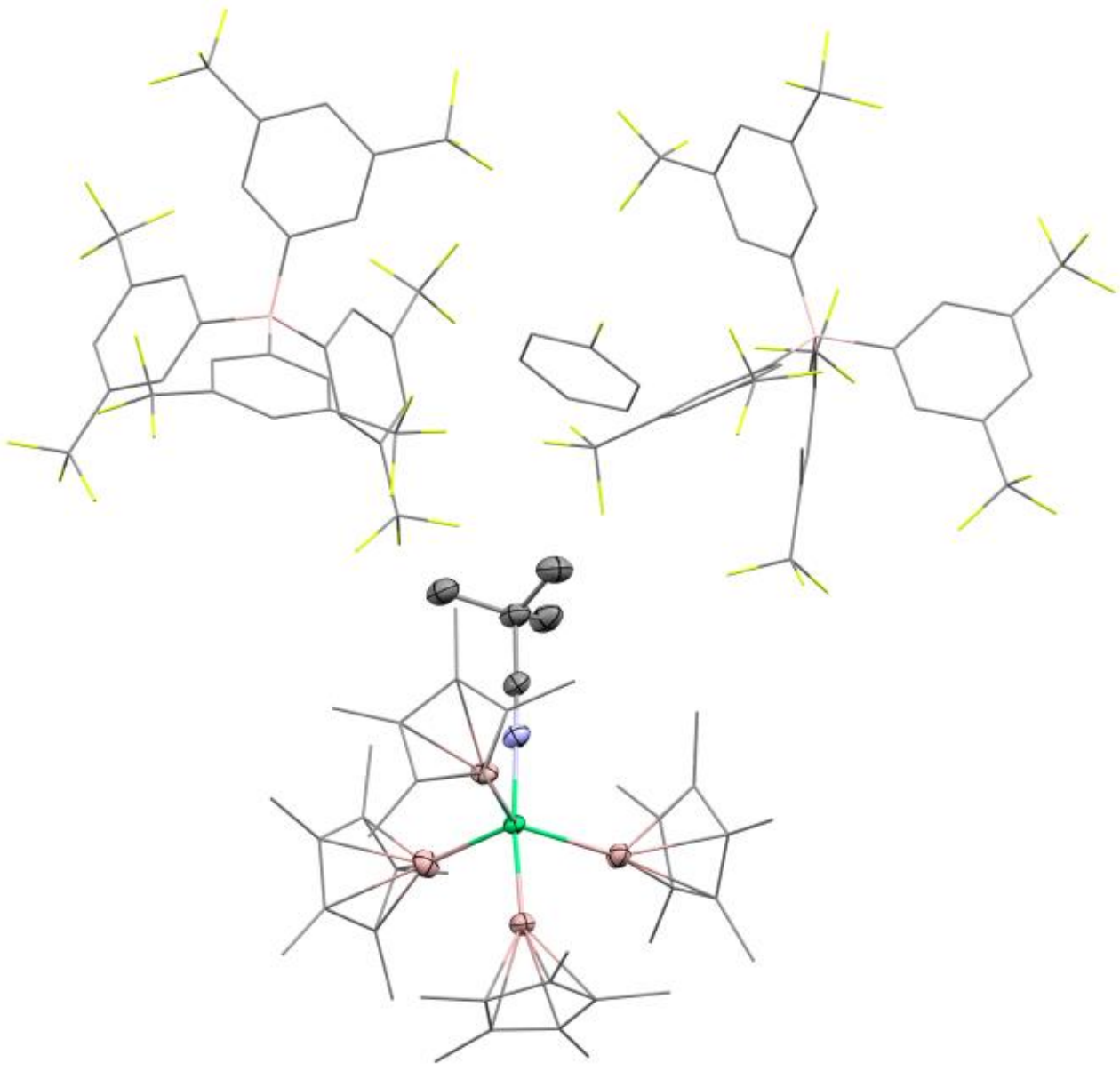

CCDC number	2500333
Empirical formula	$C_{115}H_{98}B_2F_{49}Ga_4NNi$
Formula weight	2784.15
Temperature [K]	100(2)
Crystal system	monoclinic
Space group (number)	C2/m (12)
a [Å]	24.790(3)
b [Å]	18.8529(18)
c [Å]	25.125(2)
α [°]	90
β [°]	91.456(3)
γ [°]	90
Volume [Å^3]	11739(2)
Z	4
ρ_{calc} [gcm^{-3}]	1.575
μ [mm^{-1}]	1.190
F(000)	5592
Crystal size [mm^3]	0.075×0.134×0.164
Crystal colour	violet
Crystal shape	block
Radiation	MoK_α (λ=0.71073 Å)
2θ range [°]	3.70 to 50.05 (0.84 Å)
Index ranges	−29 ≤ h ≤ 29<br>−22 ≤ k ≤ 22<br>−29 ≤ l ≤ 29
Reflections collected	375363
Independent reflections	10714<br>R_{int} = 0.0750<br>R_{sigma} = 0.0237
Completeness to θ = 25.027°	99.9 %
Data / Restraints / Parameters	10714 / 2348 / 1658
Goodness-of-fit on F^2	1.104
Final *R* indexes [I≥2σ(I)]	R_1 = 0.0581<br>wR_2 = 0.1485
Final *R* indexes [all data]	R_1 = 0.0664<br>wR_2 = 0.1549
Largest peak/hole [eÅ^{-3}]	2.10/−0.73

[(Cp*Ga)$_4$Ni(GaOTf(NC^{t}Bu)$_2$)](BAr^{F24})$_2$ (3)

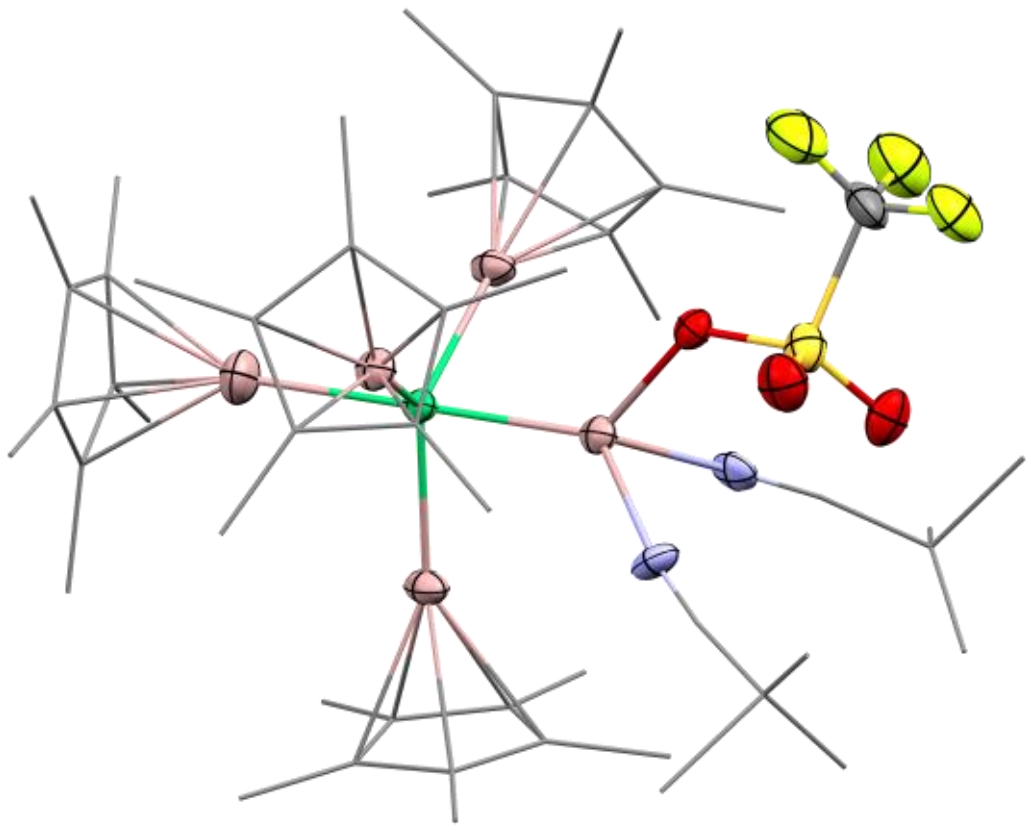

CCDC number	2535108
Empirical formula	$C_{127}H_{110}B_2F_{55}Ga_5N_2NiO_3S$
Formula weight	3218.15
Temperature [K]	100(2)
Crystal system	monoclinic
Space group (number)	*Cm* (8)
a [Å]	19.331(4)
b [Å]	34.755(7)
c [Å]	22.904(4)
α [°]	90
β [°]	109.818(6)
γ [°]	90
Volume [Å^3]	14477(5)
Z	4
ρ_{calc} [gcm^{-3}]	1.477
μ [mm^{-1}]	1.177
F(000)	6456
Crystal size [mm^3]	0.111×0.150×0.294
Crystal colour	orange
Crystal shape	block
Radiation	MoK_α (λ=0.71073 Å)
2θ range [°]	3.78 to 50.05 (0.84 Å)
Index ranges	−23 ≤ h ≤ 23<br>−41 ≤ k ≤ 41<br>−27 ≤ l ≤ 27
Reflections collected	26025
Independent reflections	26025<br>R_{int} = 0.1098<br>R_{sigma} = 0.0850
Completeness to θ = 25.026°	100.0
Data / Restraints / Parameters	26025 / 5525 / 2605
Goodness-of-fit on F^2	1.050
Final *R* indexes [I≥2σ(I)]	R_1 = 0.1083<br>wR_2 = 0.2860
Final *R* indexes [all data]	R_1 = 0.1303<br>wR_2 = 0.3116
Largest peak/hole [eÅ^{-3}]	1.26/−3.03
Flack X parameter	0.009(9)

Refined as a two-component inversion twin.

Comments on the refinement of **3**:

3 was refined as an inversion twin in the non-centrosymmetric space group *Cm*. The BASF parameter refined to a final value of 0.46079, which comes quite close to “perfect” racemic twinning. The structure of **3** comprises two complex units with one located right at the (0,0,0) mirror plane and another one close to the (0,0.5,0) mirror plane of the monoclinic unit cell. The two congeners exhibit different levels of quality, as found during refinement of the crystal structure, with the first complex unit (with Ni1 as the central atom) right at the (0,0,0) mirror plane having the higher quality. The second unit (with Ni2 as the central atom) is disordered around the mirror plane, and due to poor crystal quality, parts of the ligand sphere as well as of the BAr^{F24} anions were refined isotropically. Therefore, the discussion of the crystal structure in the main text refers only to the first congener with Ni1 as the central metal. Nevertheless, data quality was high enough to allow for an unambiguous assignment of the absolute structure *via* anomalous dispersion, as deduced from the low Flack X parameter of 0.009(9).

[(η⁵-C_5Me_5)Ni(η⁴-C_5Me_5H)]BAr^{F20}

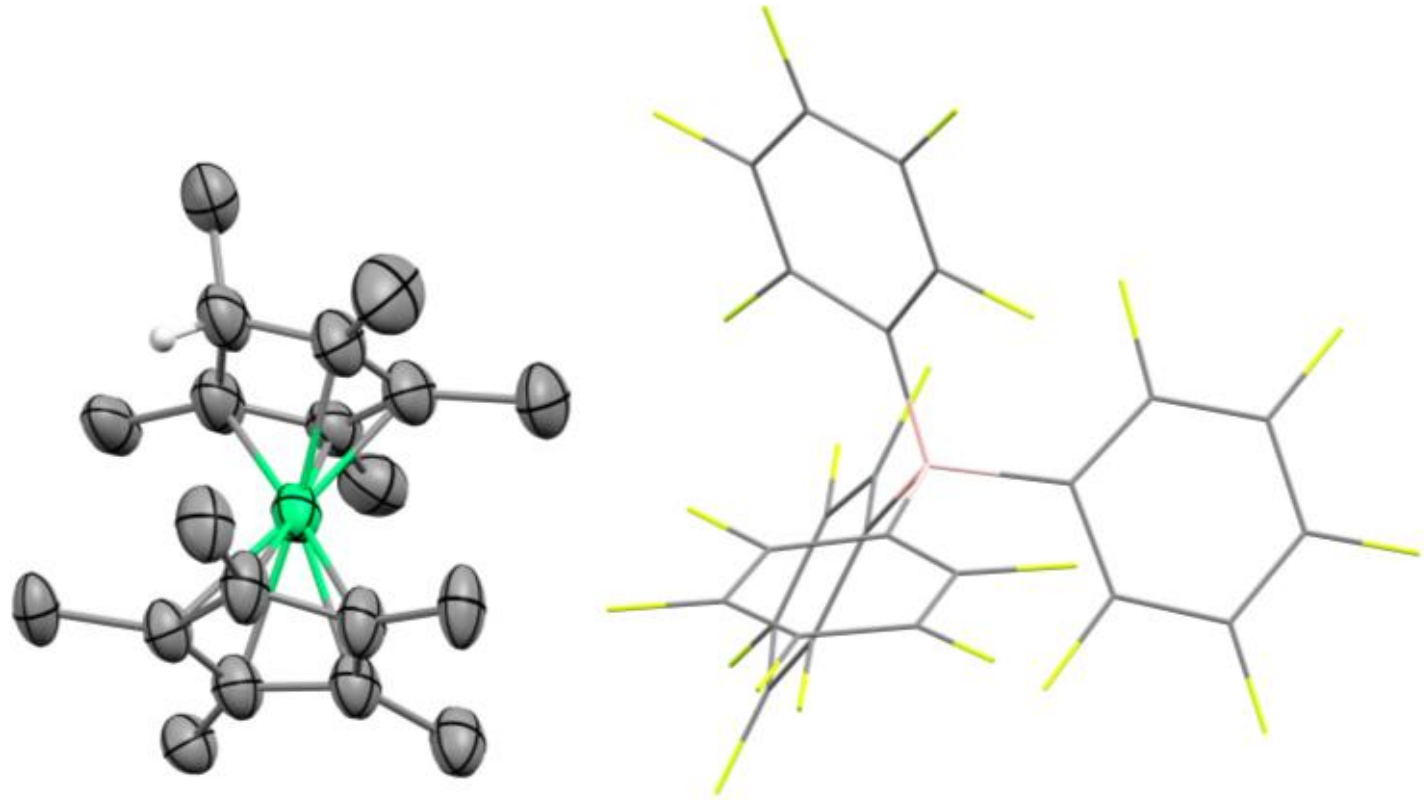

CCDC number	2500335
Empirical formula	$C_{44}H_{31}BF_{20}Ni$
Formula weight	1009.21
Temperature [K]	100(2)
Crystal system	triclinic
Space group (number)	$P\bar{1}$ (2)
a [Å]	10.847(3)
b [Å]	13.030(3)
c [Å]	14.609(4)
α [°]	80.712(8)
β [°]	87.048(8)
γ [°]	80.173(7)
Volume [Å³]	2007.2(8)
Z	2
ρ_{calc} [gcm^{-3}]	1.670
μ [mm^{-1}]	0.611
F(000)	1016
Crystal size [mm³]	0.099×0.210×0.318
Crystal colour	green
Crystal shape	block
Radiation	MoK_α (λ=0.71073 Å)
2θ range [°]	3.93 to 50.05 (0.84 Å)
Index ranges	−12 ≤ h ≤ 12<br>−15 ≤ k ≤ 15<br>−17 ≤ l ≤ 17
Reflections collected	52440
Independent reflections	7075<br>R_{int} = 0.0605<br>R_{sigma} = 0.0398
Completeness to θ = 25.027°	99.7
Data / Restraints / Parameters	7075 / 1569 / 899
Goodness-of-fit on F^2	1.066
Final *R* indexes [I≥2σ(I)]	R_1 = 0.1234<br>wR_2 = 0.2512
Final *R* indexes [all data]	R_1 = 0.1819<br>wR_2 = 0.2879
Largest peak/hole [eÅ^{-3}]	0.65/−0.70
Extinction coefficient	0.0135(12)

[(Cp*)Ni(GaCp*)$_2$]BAr^{F20}

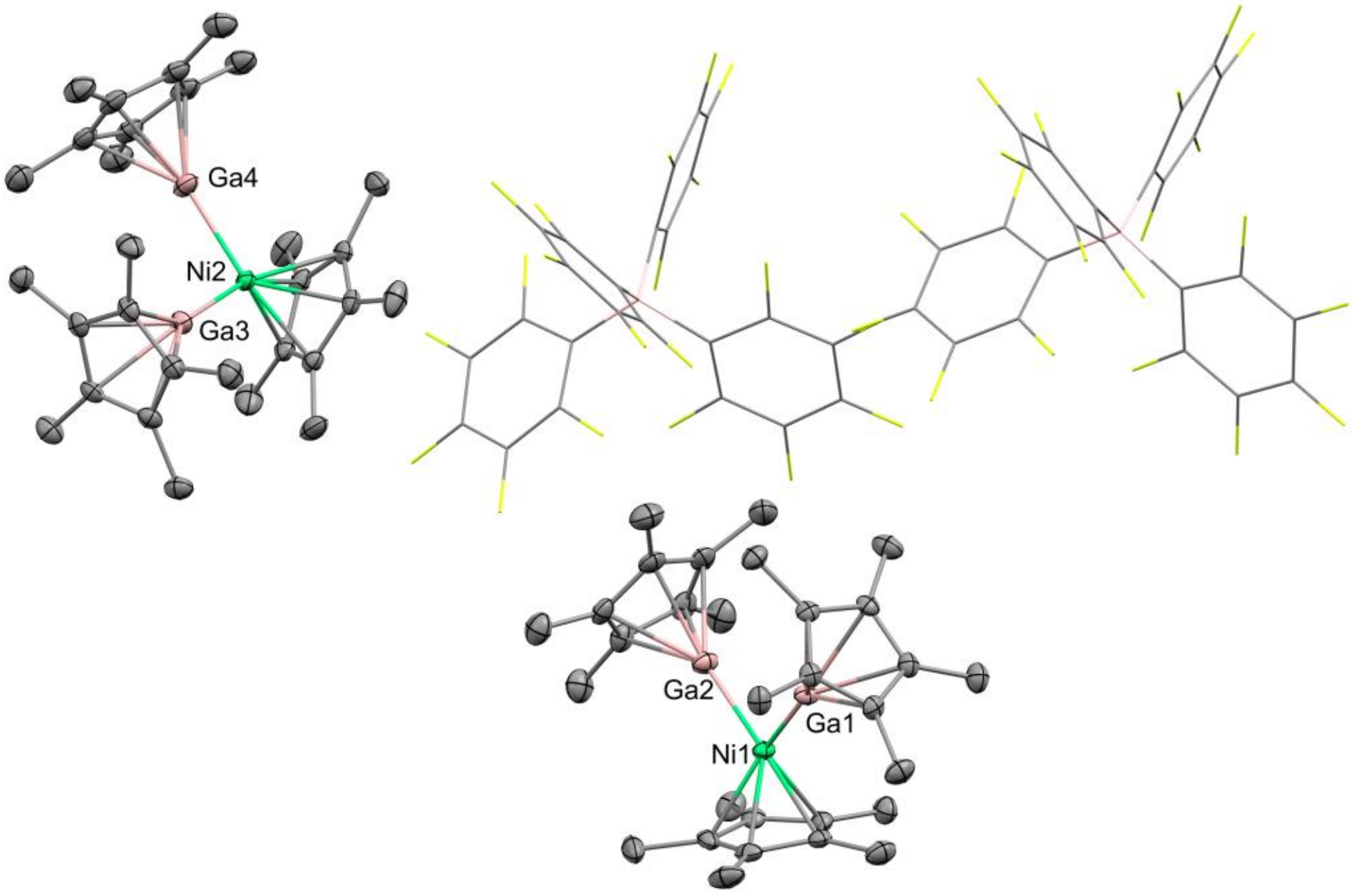

CCDC number	2500336
Empirical formula	$C_{54}H_{45}BF_{20}Ga_2Ni$
Formula weight	1282.86
Temperature [K]	100(2)
Crystal system	triclinic
Space group (number)	$P\overline{1}$ (2)
a [Å]	11.1250(4)
b [Å]	20.8412(9)
c [Å]	23.4926(11)
α [°]	96.545(2)
β [°]	99.5440(10)
γ [°]	102.5730(10)
Volume [Å^3]	5178.6(4)
Z	4
ρ_{calc} [gcm^{-3}]	1.645
μ [mm^{-1}]	1.503
F(000)	2576
Crystal size [mm^3]	0.089×0.131×0.198
Crystal colour	orange
Crystal shape	block
Radiation	MoK_α (λ=0.71073 Å)
2θ range [°]	3.81 to 52.90 (0.80 Å)
Index ranges	$-13 \le h \le 13$<br>$-26 \le k \le 26$<br>$-29 \le l \le 29$
Reflections collected	357331
Independent reflections	21258<br>R_{int} = 0.0684<br>R_{sigma} = 0.0234
Completeness to θ = 25.242°	100.0
Data / Restraints / Parameters	21258 / 0 / 1435
Goodness-of-fit on F^2	1.193
Final *R* indexes [$I \geq 2\sigma(I)$]	R_1 = 0.0326<br>wR_2 = 0.0702
Final *R* indexes [all data]	R_1 = 0.0580<br>wR_2 = 0.0877
Largest peak/hole [eÅ^{-3}]	0.48/−0.75

[(η⁵-C_5Me_5)Ni(η⁴-C_5Me_5H)]BAr^{F24}

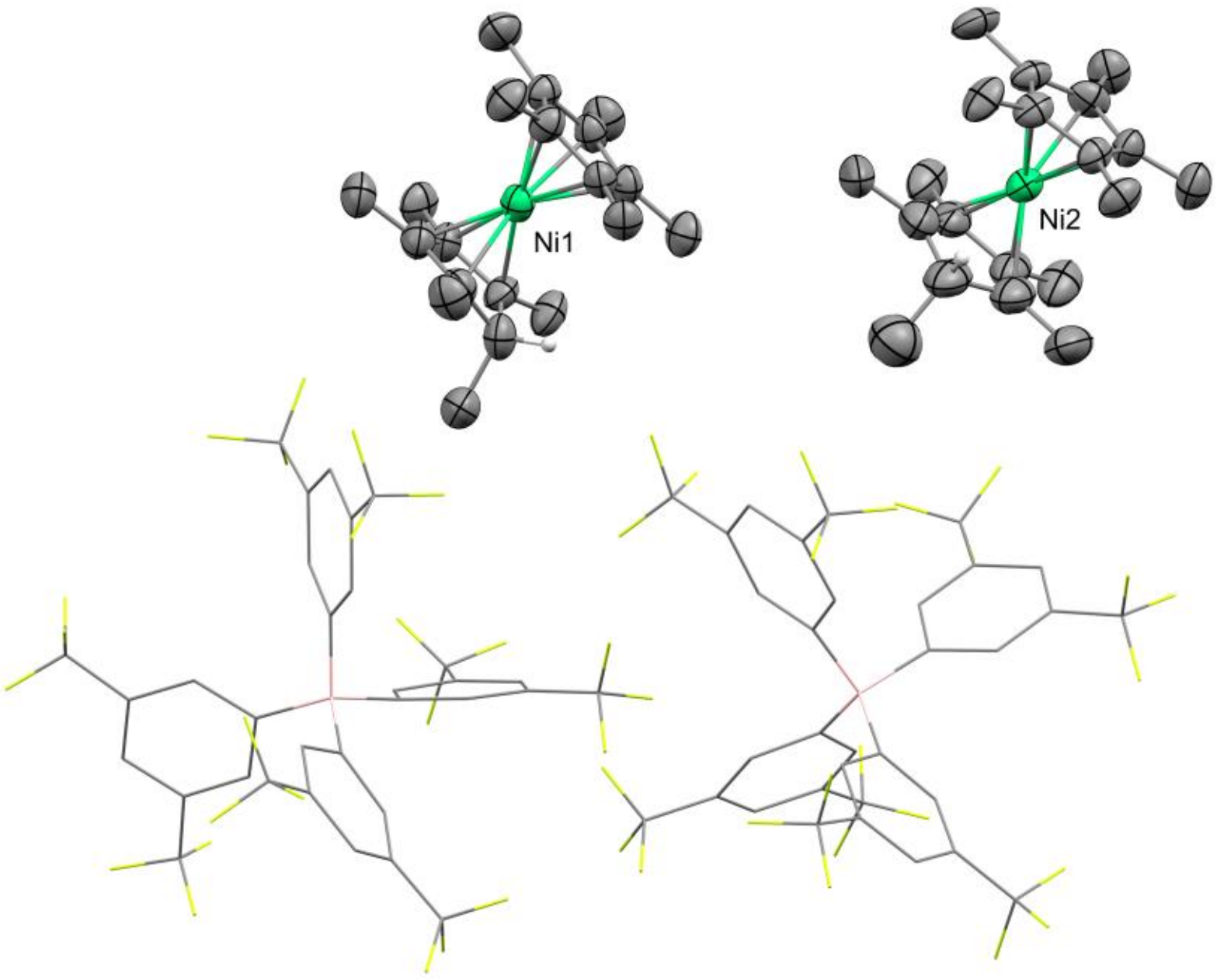

CCDC number	2500334
Empirical formula	$C_{104}H_{86}B_2F_{48}Ni_2$
Formula weight	2386.76
Temperature [K]	100(2)
Crystal system	monoclinic
Space group (number)	$P2_1$ (4)
a [Å]	11.5371(8)
b [Å]	34.774(3)
c [Å]	12.8914(10)
α [°]	90
β [°]	97.986(2)
γ [°]	90
Volume [Å^3]	5121.7(7)
Z	2
ρ_{calc} [gcm^{-3}]	1.548
μ [mm^{-1}]	0.503
F(000)	2416
Crystal size [mm^3]	0.196×0.237×0.439
Crystal colour	green
Crystal shape	block
Radiation	MoK_α (λ=0.71073 Å)
2θ range [°]	3.75 to 53.07 (0.80 Å)
Index ranges	−14 ≤ h ≤ 14<br>−43 ≤ k ≤ 43<br>−16 ≤ l ≤ 16
Reflections collected	201017
Independent reflections	21174<br>R_{int} = 0.0487<br>R_{sigma} = 0.0263
Completeness to θ = 25.242°	100.0
Data / Restraints / Parameters	21174 / 3037 / 1883
Goodness-of-fit on F^2	1.180
Final *R* indexes [I≥2σ(I)]	R_1 = 0.0875<br>wR_2 = 0.2331
Final *R* indexes [all data]	R_1 = 0.1249<br>wR_2 = 0.2942
Largest peak/hole [eÅ^{-3}]	1.35/−0.91
Extinction coefficient	0.0111(14)

[Ni(NC^{t}Bu)$_6$](BAr^{F20})$_2$

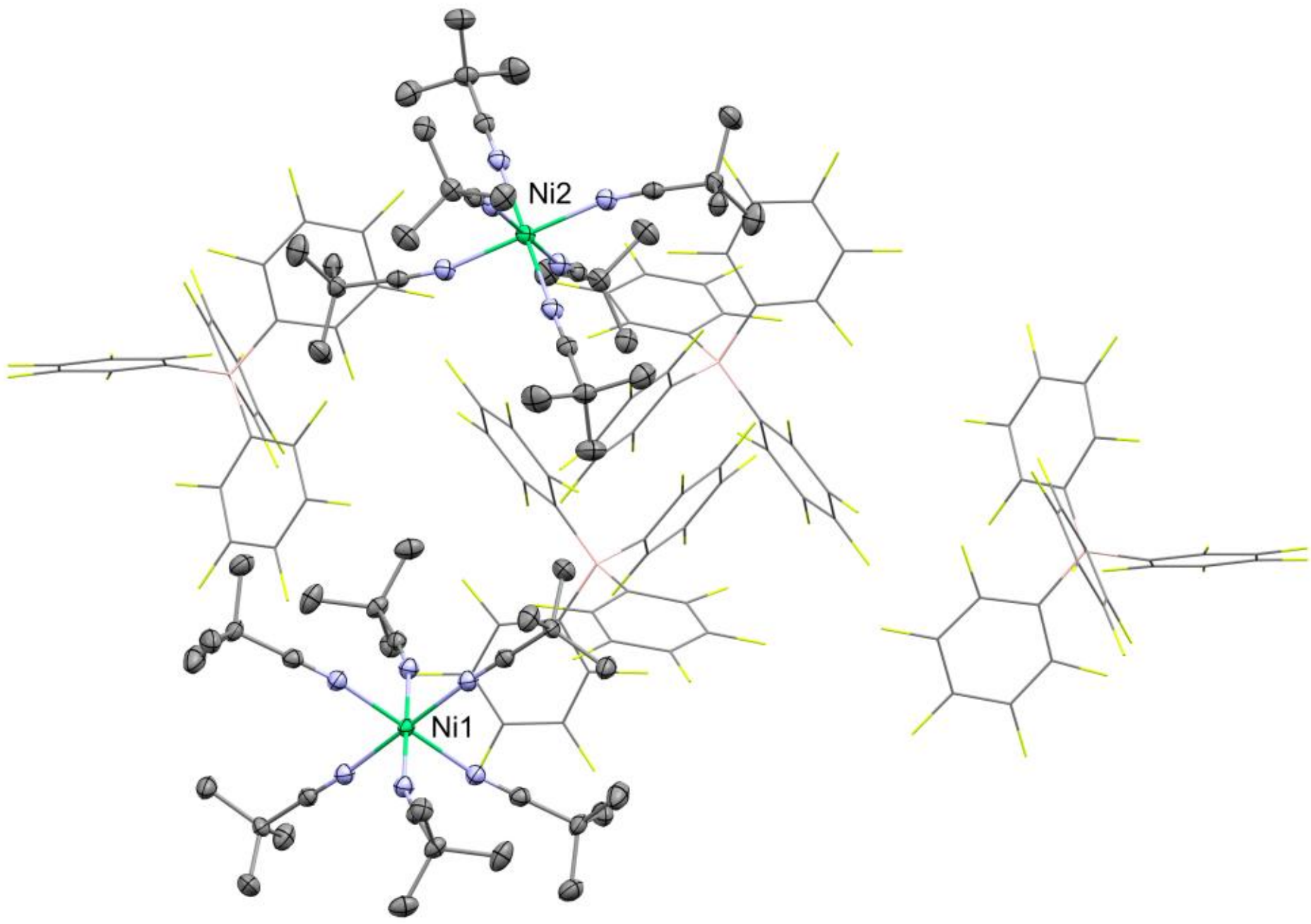

CCDC number	2500337
Empirical formula	$C_{78}H_{54}B_2F_{40}N_6Ni$
Formula weight	1915.60
Temperature [K]	100(2)
Crystal system	triclinic
Space group (number)	$P\bar{1}$ (2)
a [Å]	14.3435(6)
b [Å]	15.5451(7)
c [Å]	19.6857(10)
α [°]	95.728(2)
β [°]	107.041(2)
γ [°]	94.822(2)
Volume [Å^3]	4145.9(3)
Z	2
ρ_{calc} [gcm^{-3}]	1.534
μ [mm^{-1}]	0.371
F(000)	1924
Crystal size [mm^3]	0.050×0.109×0.115
Crystal colour	colourless
Crystal shape	plate
Radiation	MoK_α (λ=0.71073 Å)
2θ range [°]	4.05 to 50.05 (0.84 Å)
Index ranges	$-17 \le h \le 17$<br>$-18 \le k \le 18$<br>$-23 \le l \le 23$
Reflections collected	107684
Independent reflections	14628<br>R_{int} = 0.0971<br>R_{sigma} = 0.0467
Completeness to θ = 25.027°	99.8
Data / Restraints / Parameters	14628 / 0 / 1165
Goodness-of-fit on F^2	1.029
Final *R* indexes [$I \ge 2\sigma(I)$]	R_1 = 0.0318<br>wR_2 = 0.0754
Final *R* indexes [all data]	R_1 = 0.0471<br>wR_2 = 0.0799
Largest peak/hole [eÅ^{-3}]	0.27/−0.42

4. X-Ray Photoelectron Spectroscopy

General comments

To gain further insights into the electronic structure of [$(Cp^*Ga)_4Ni(GaF(NC^tBu)_2)](BAr^{F24})_2$ (**1**) and elucidate the oxidation state of the GaF moiety spectroscopically, we performed XPS measurements under strictly air- and moisture-free conditions. We performed Ga 2p and 3d XPS measurements for GaDDP (DDP: 2-(2,6-diisopropylphenyl)amino-4-(2,6-diisopropylphenyl)imino-2-pentene) as a non-volatile, monomeric Ga(**I**) complex for internal reference. These data were used for cross-validation and were virtually identical to previous reports on GaDDP by *Baines* and coworkers[35] after appropriate background subtraction and referencing.

Referencing & comparison

To ensure reasonable comparability, we chose [$Ni(GaCp^*)_4(GaN_2C_{14}H_{21})](BAr^{F24})_2$ (from now on denominated as $NiGa_5N_2$), a product of acetonitrile activation previously reported by our group,[3] as a suitable reference compound. Similar to **1**, $NiGa_5N_2$ contains formally one Ni(**II**) and only Ga(**I**) centres, four of which are GaCp* units and one as a Ga(I) β-diketiminate with gallium in a slightly differing coordination environment (see Figure S2).

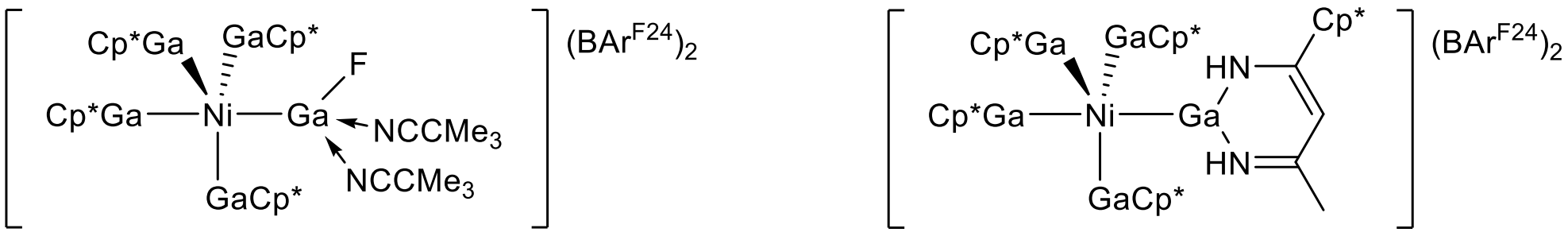

1 **$NiGa_5N_2$**

Figure S2. Chemical structures of **1** and $NiGa_5N_2$, which exhibit a formal Ni(II) centre and five Ga(I) centres, four of which are GaCp* each.

The binding energies were corrected for the F 1s peak at 688.5 eV for the CF_3 groups of the BAr^{F24} anions, which are all present in both compounds **1** and $NiGa_5N_2$. In contrast to carbon, which occurs as both sp^2 and sp^3 carbon atoms in all measured samples, the F 1s signal provides a more reasonable signal for binding energy correction, since fluorine is present in CF_3 groups and therefore contained in a single chemical environment in all compounds. For both substances, only one peak was detected for both the Ni 2p and the Ga 2p signal. The Ga 2p features appear slightly broadened, most probably as a consequence of slightly differing chemical gallium environments with Cp* and a nacnac or fluoride ligand. Table S2 compares selected Ni 2p and Ga 2p binding energies for both **1** and $NiGa_5N_2$, which are almost identical. As stated in the main text, the Ga 2p spectrum of **1**, acquired using Al K_α radiation, exhibits peaks at binding energies of 1119.1 eV (Ga $2p_{3/2}$) and 1146.0 eV (Ga $2p_{1/2}$). Previous XPS studies on organometallic gallium complexes show that three tested Ga(**I**) compounds with differing ligand environments comprise comparable $2p_{3/2}$ binding energies between 1117.5 and 1119.3 eV.[35] This is in good agreement with a formal oxidation state of +I for all Ga atoms in **1**. In addition to Al K_α radiation, we also used Ag K_α radiation for XPS measurements, specifically for the Ni 2p signal. For Al, there are several overlaps between the Ni 2p peak and a shake-up feature in the F KLL Auger, as well

as complex satellite structures and the chemical shift of the Ni 2p peak, all of which are difficult to distinguish. Using Ag $K_α$ radiation, we detected a single species at 855.2 eV (Ni $2p_{3/2}$) and 872.5 (Ni $2p_{1/2}$), which points towards a Ni(**II**) centre in **1**. Comparison with literature data suggests that **1** contains an oxidised [presumably Ni(**II**)] nickel centre, as it fits well to the binding energies of several Ni(**II**) complexes ($2p_{3/2}$: 856.9-854.3 eV; $2p_{1/2}$: 871.4-874.3 eV)[36] as well as Ni(**II**) halides and oxides or Ni(**III**) oxides and hydroxides (853.7-858.1 eV for $2p_{3/2}$)[37-38] than to those of elemental nickel (852.6 eV for $2p_{3/2}$).[37] Therefore, we assigned the (formal) oxidation states of +**II** for Ni and +**I** for all Ga atoms in **1**. The individual spectra are depicted in Figures S3-S6.

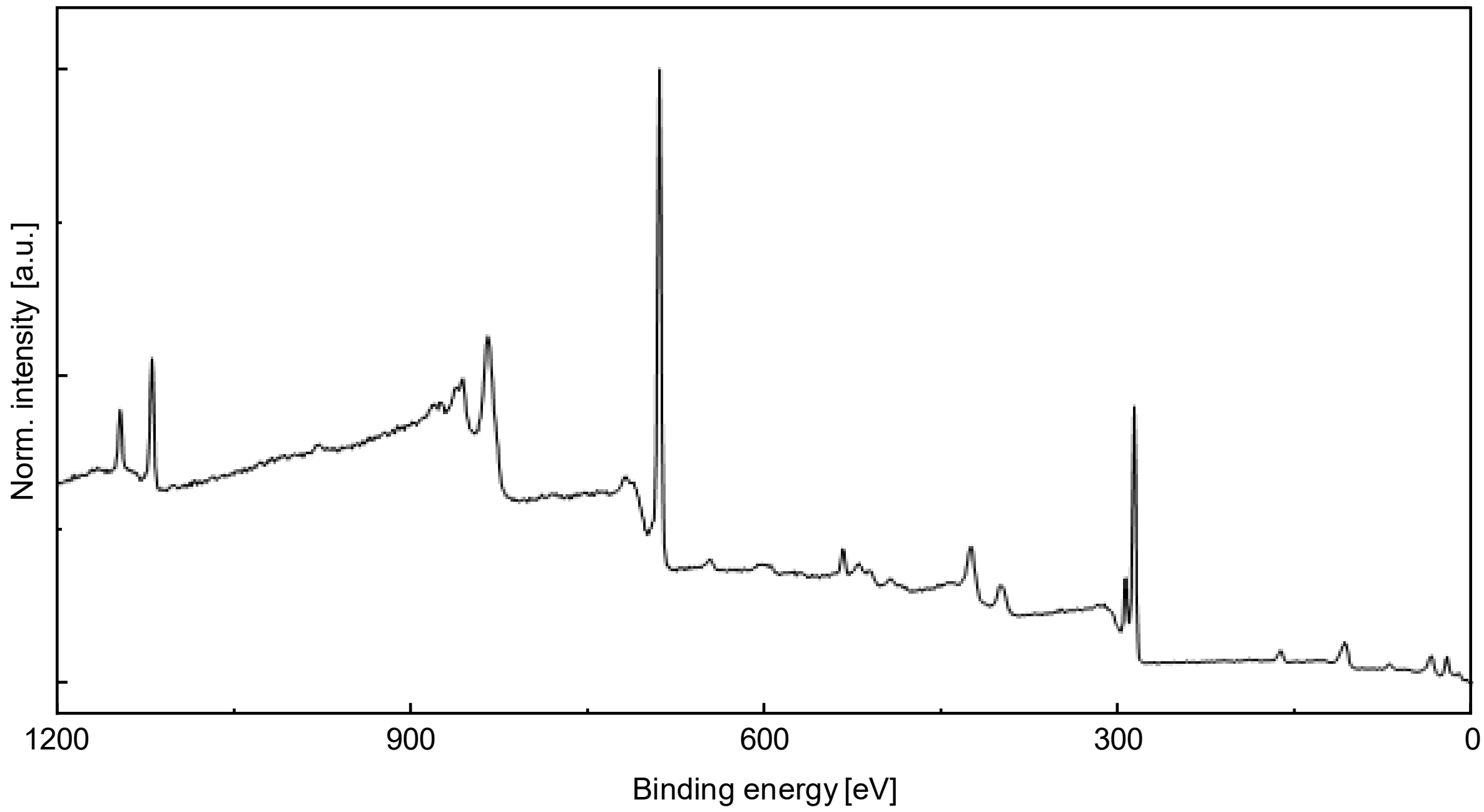

Figure S3. XPS wide scan of **1** with binding energies between 1200 and 0 eV, using Al $K_α$ radiation.

Table S2. Comparison of selected binding energies of Ni 2p and Ga 2p signals for **1** and $NiGa_5N_2$.

	**Binding energy [eV]**	
**Peak**	**1**	**$NiGa_5N_2$**
Ga $2p_{3/2}$	1119.1	1118.7
Ga $2p_{1/2}$	1146.0	1145.6
Ni $2p_{3/2}$ (Al $K_α$)	855.5	855.1
Ni $2p_{1/2}$ (Al $K_α$)	872.8	872.4
Ni 2p satellites (Al $K_α$)	862.2; 879.5	862.1; 879.4
Ni $2p_{3/2}$ (Ag $K_α$)	855.2	854.8
Ni $2p_{1/2}$ (Ag $K_α$)	872.5	872.1

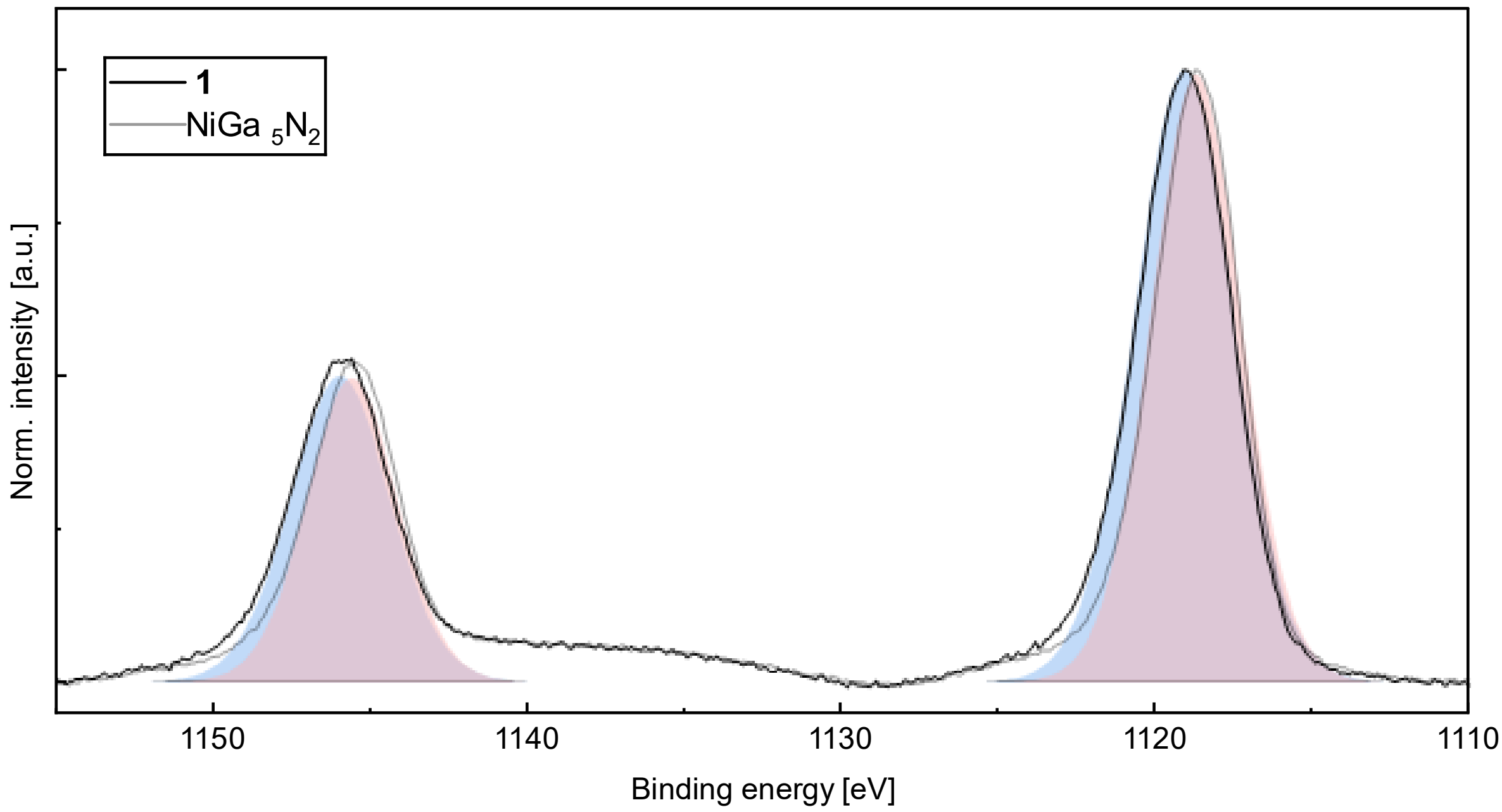

Figure S4. Comparison of the detailed spectra of the Ga 2p peak of **1** and $NiGa_5N_2$, using Al K_α radiation, including the peak fit model. Within the error of measurement and energy correction, the slight shift of 0.4 eV suggests a nearly identical electronic structure for both compounds, which strongly suggests the presence of only Ni(II) and Ga(I) for **1**.

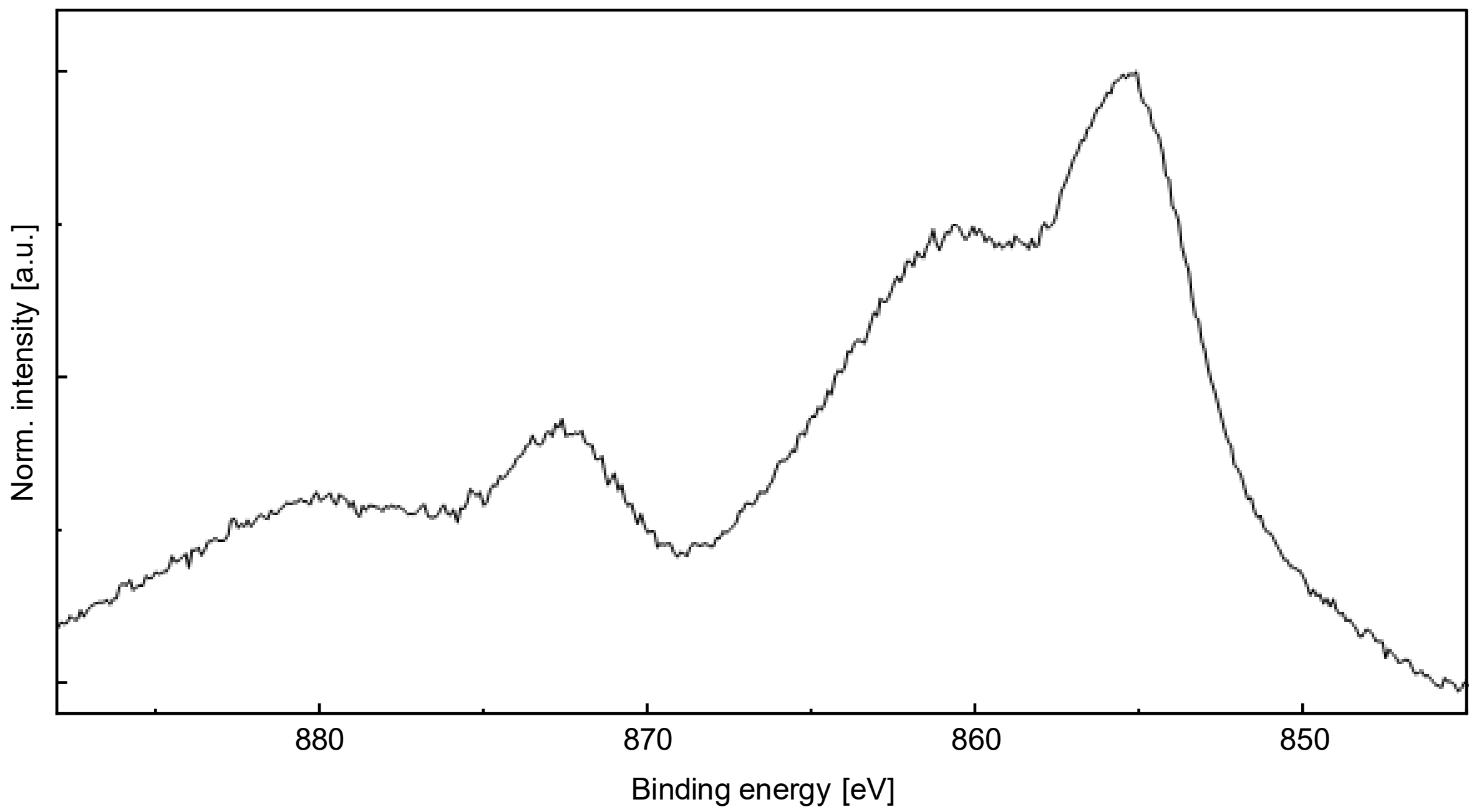

Figure S5. Detailed spectrum of the Ni 2p peak of **1**, acquired using Al K_α radiation. The peak allows estimation of the Ni 2p binding energies, but it is largely obscured by a shake-up feature from the neighbouring F KLL Auger. The complex satellite structures and the chemical shift of the Ni 2p peak prevent a clear distinction during peak fitting.

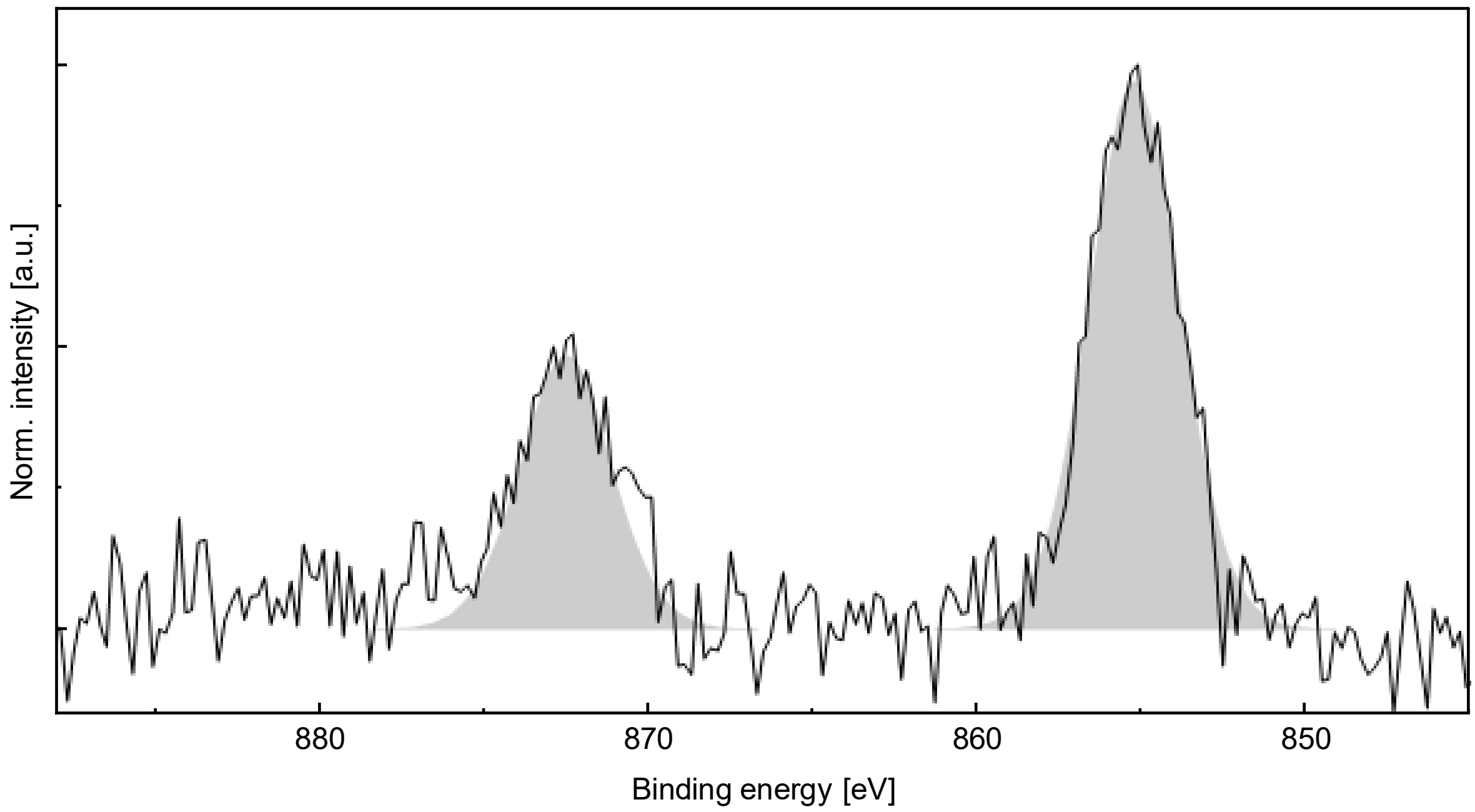

Figure S6. Detailed spectrum of the Ni 2p peak of **1**, acquired using Ag K_{α} radiation, including the utilised peak fit. The higher energy of Ag radiation allows clear identification of the Ni 2p peak without obscuring other signals.

5. Computational Part

Table S3. Comparison of selected bond lengths of the DFT optimised and the experimentally determined structure of the dication $[(Cp^*Ga)_4Ni(GaF(NC^tBu)_2)]^{2+}$.

Bond	Calculated [Å]	Experimental (SC-XRD) [Å]
Ga1–F1	1.790	1.8097(15)
Ga1–N1	2.110	2.106(2)
Ga1–N2	2.104	2.101(2)
Ni1–Ga1	2.239	2.2490(4)
Ni1–Ga2	2.273	2.2624(4)
Ni1–Ga3	2.273	2.2810(4)
Ni1–Ga4	2.258	2.2560(4)
Ni1–Ga5	2.255	2.2643(4)
Ga1…Ga2[a)]	2.991	2.9568(4)
Ga1…Ga3[a)]	3.047	3.0656(4)
Ga1…Ga4[a)]	3.100	3.0915(4)

a) Stabilising $Ga^I\cdots Ga^I$ interactions are not uncommon in transition-metal complexes containing several GaR ligands. This feature has been recently analysed in detail in a different contribution (see Antsiburov, Bühler, Stephan *et. al. Chem. Sci.* ***2026****, 17*, 8989-8997).[39]

Table S4. Comparison of selected angles within the DFT optimised and the experimentally determined structure of the dication $[(Cp^*Ga)_4Ni(GaF(NC^tBu)_2)]^{2+}$.

Angle	Calculated [°]	Experimental (SC-XRD) [°]
Ga1–Ni1–Ga5	176.70	175.914(17)
Ga1–Ni1–Ga2	83.05	81.898(13)
Ga1–Ni1–Ga3	84.94	85.173(13)
Ga1–Ni1–Ga4	87.16	86.667(14)
Ga2–Ni1–Ga3	113.78	112.091(14)
Ga3–Ni1–Ga4	117.75	119.433(15)
Ga4–Ni1–Ga2	126.26	125.835(15)

Table S5. Computed NAO and QTAIM atomic charges in $[(Cp^*Ga)_4Ni(GaF(NC^tBu)_2)]^{2+}$, $[(Cp^*Ga)_4Ni(GaF)]^{2+}$ and free GaF.

	$[(Cp^*Ga)_4Ni(GaF(NC^tBu)_2)]^{2+}$		$[(Cp^*Ga)_4Ni(GaF)]^{2+}$		GaF (free ligand)	
	NAO	QTAIM	NAO	QTAIM	NAO	QTAIM
Ni	–0.33	–0.46	–0.35	–0.48	-	-
Ga1	+1.13	+1.29	+1.26	+0.98	+0.78	+0.79
Ga5	+0.78	+0.75	+0.80	+0.81	-	-
Ga_{eq} (av.)	+0.84	+0.73	+0.72	+0.74	-	-
F	–0.76	–0.77	–0.77	–0.72	–0.78	–0.79
N (av.)	–0.51	–1.33	-	-	-	-

Table S6. Selected topological bcp descriptors in $[(Cp^*Ga)_4Ni(GaF(NC^tBu)_2)]^{2+}$ (first value) and $[(Cp^*Ga)_4Ni(GaF)]^{2+}$ (second value). ρ, $\nabla^2\rho$, H, V and G are the electron density, Laplacian of ρ, energy density, potential energy density and kinetic energy density values at the bcp, respectively. δ is the delocalisation index. All values in a.u.

bcp descriptors	Ni–Ga1	Ni–Ga5	Ni–Ga_{eq} (av.)	Ga1–N (av.)
ρ	0.078/0.087	0.072/0.079	0.069/0.070	0.066
$\nabla^2\rho$	0.069/0.143	0.084/0.085	0.102/0.097	0.229
H	–0.035/–0.037	–0.029/–0.035	–0.025/–0.026	–0.012
V	–0.088/–0.110	–0.078/–0.090	–0.075/–0.077	–0.081
$\lvert V \rvert$ / G	1.673/1.510	1.576/1.620	1.496/1.520	1.172
δ	0.804/0.934	0.840/0.873	0.826/0.803	0.322

Table S7. Various computed indicators relative to the Ga–F bond in $[(Cp^*Ga)_4Ni(GaF(NC^tBu)_2)]^{2+}$, $[(Cp^*Ga)_4Ni(GaF)]^{2+}$ and free GaF. WBI is the Wiberg bond index; δ is the QTAIM delocalisation index; ρ, $\nabla^2\rho$, H, V and G are the electron density, Laplacian of ρ, energy density, potential energy density and kinetic energy density values at the bcp, respectively. All values in a.u.

	$[(Cp^*Ga)_4Ni(GaF(NC^tBu)_2)]^{2+}$	$[(Cp^*Ga)_4Ni(GaF)]^{2+}$	GaF (free ligand)
Distance (Å)	1.790	1.733	1.788
WBI	0.329	0.311	0.330
δ	0.537	0.684	0.795
	QTAIM bcp descriptors		
ρ	0.125	0.145	0.124
$\nabla^2\rho$	0.714	0.864	0.737
H	–0.030	–0.043	–0.028
V	–0.238	–0.302	–0.240
$\lvert V \rvert / G$	1.142	1.166	1.130

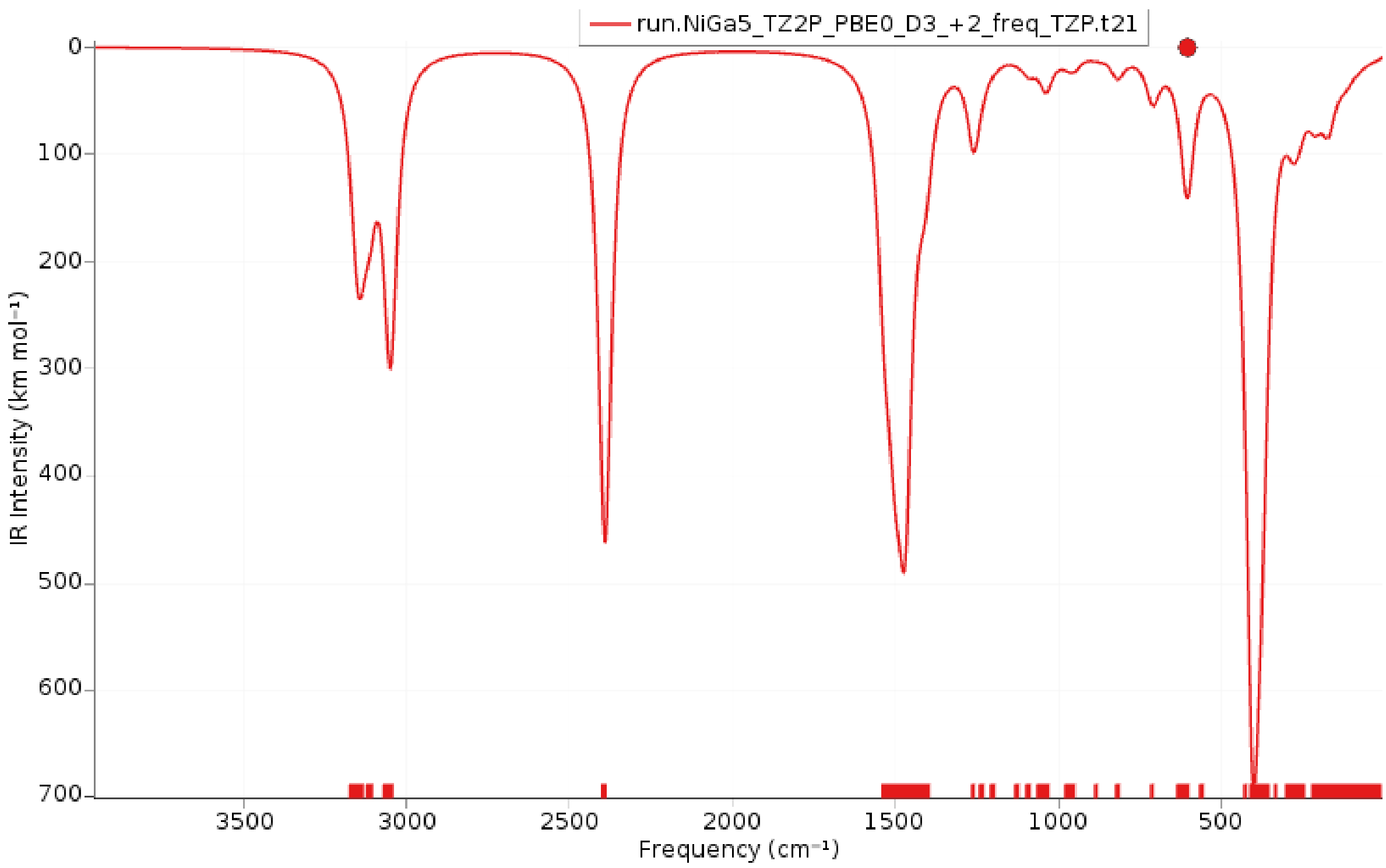

Figure S7. Simulated IR spectrum of the $[(Cp^*Ga)_4Ni(GaF(NC^tBu)_2)]^{2+}$ dication. The strong band at 2393 cm^{-1} corresponds to the nitrile C-N stretching vibration, and the weak band at 609 cm^{-1} (highlighted with a red dot) to the Ga–F stretching vibration.

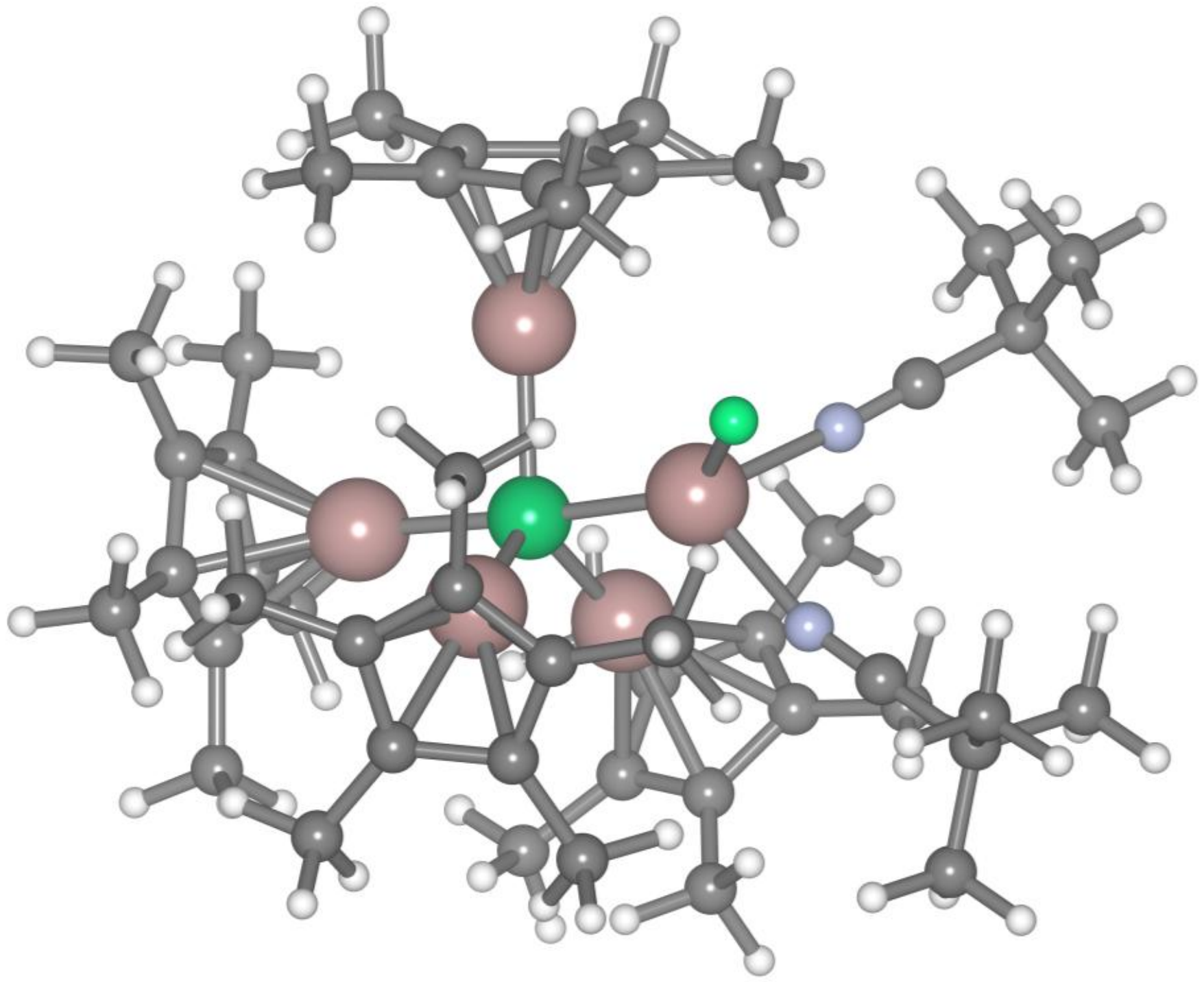

Figure S8. Optimised structure of the $[(Cp^*Ga)_4Ni(GaF(NC^tBu)_2)]^{2+}$ dication.

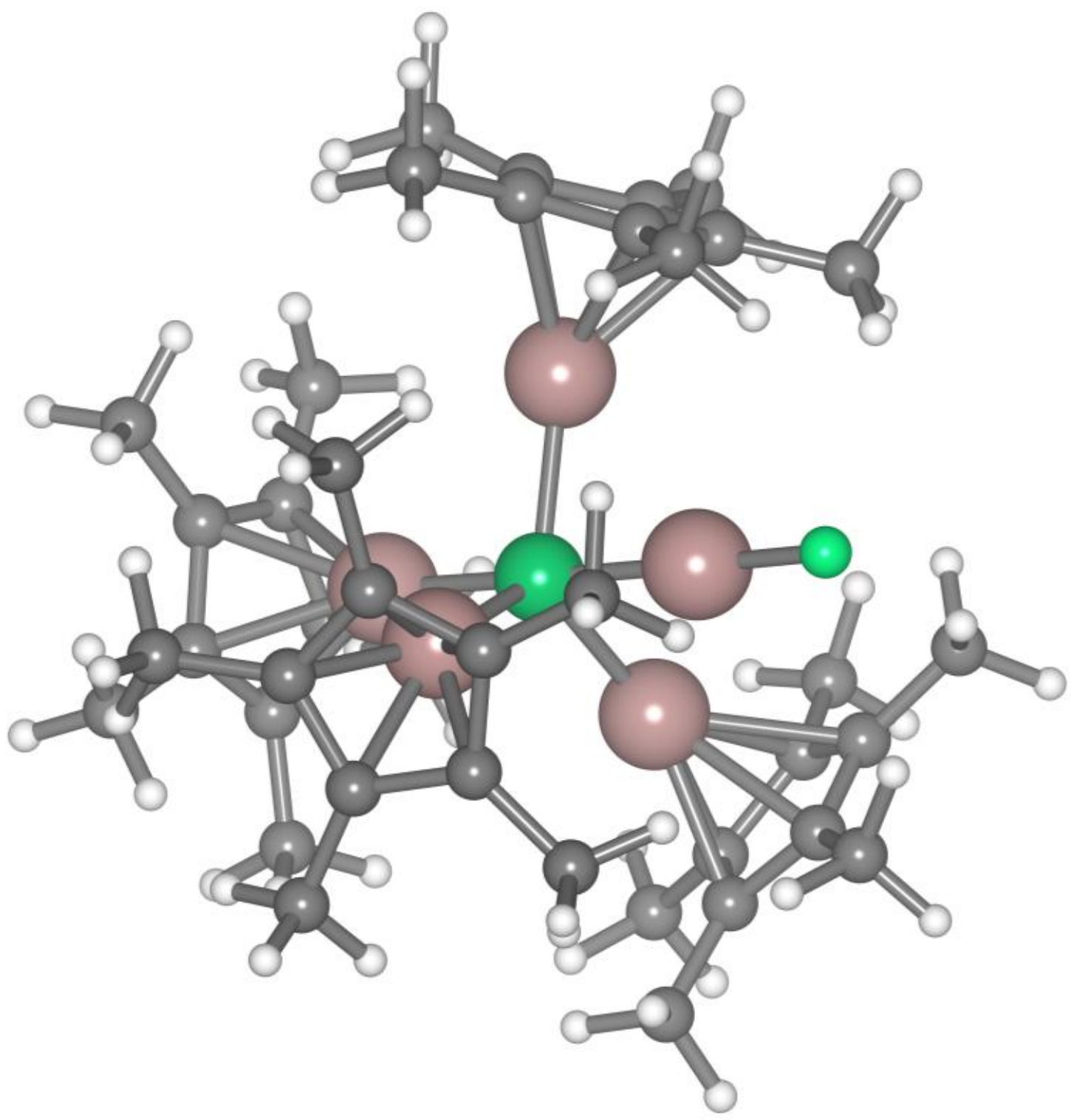

Figure S9. Optimised structure of the $[(Cp^*Ga)_4Ni(GaF)]^{2+}$ dication.

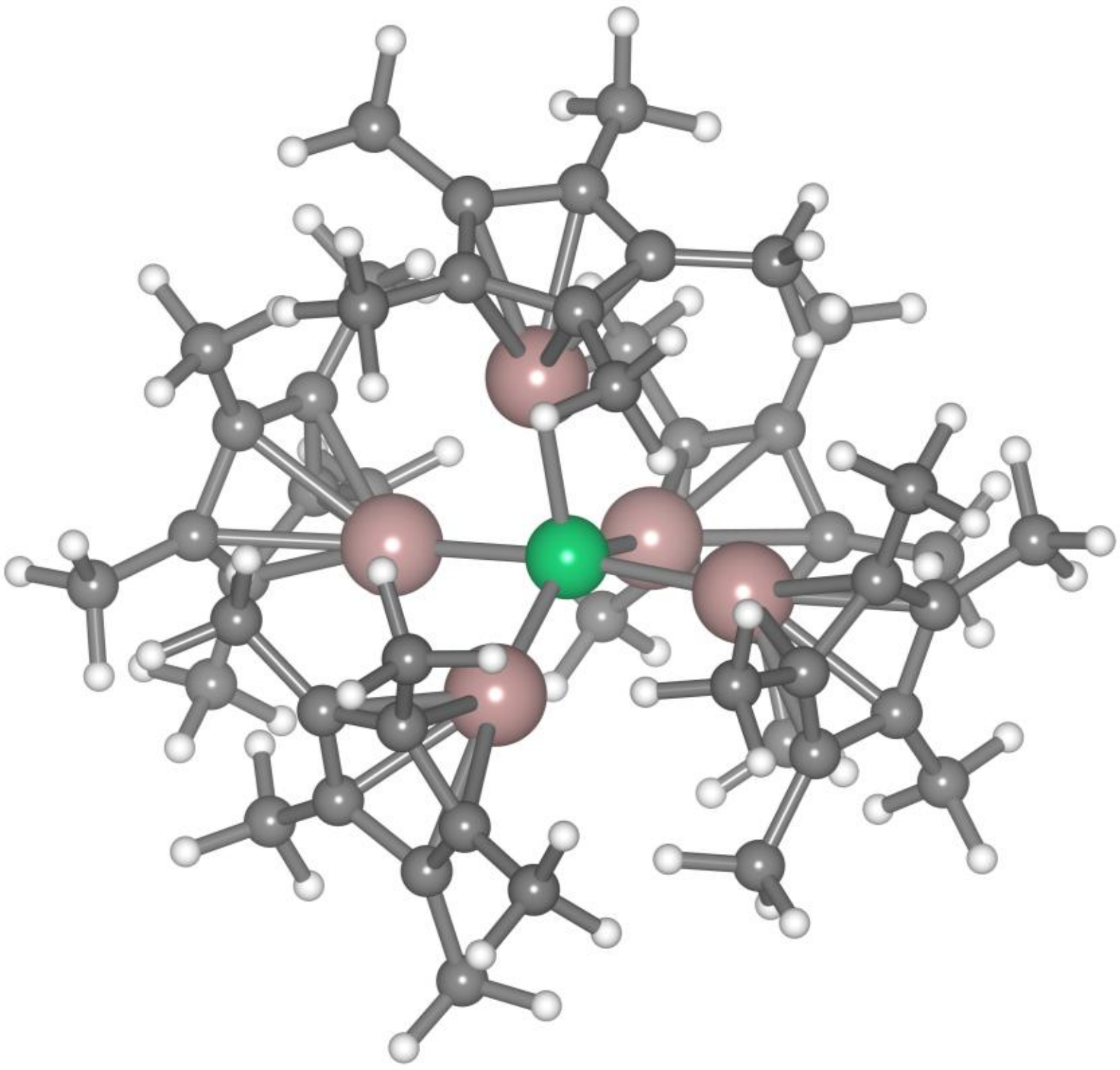

Figure S10. Optimised structure of the $[(Cp^*Ga)_4Ni]^{2+}$ dication.

6. NMR & Additional Spectra

Compound (1)

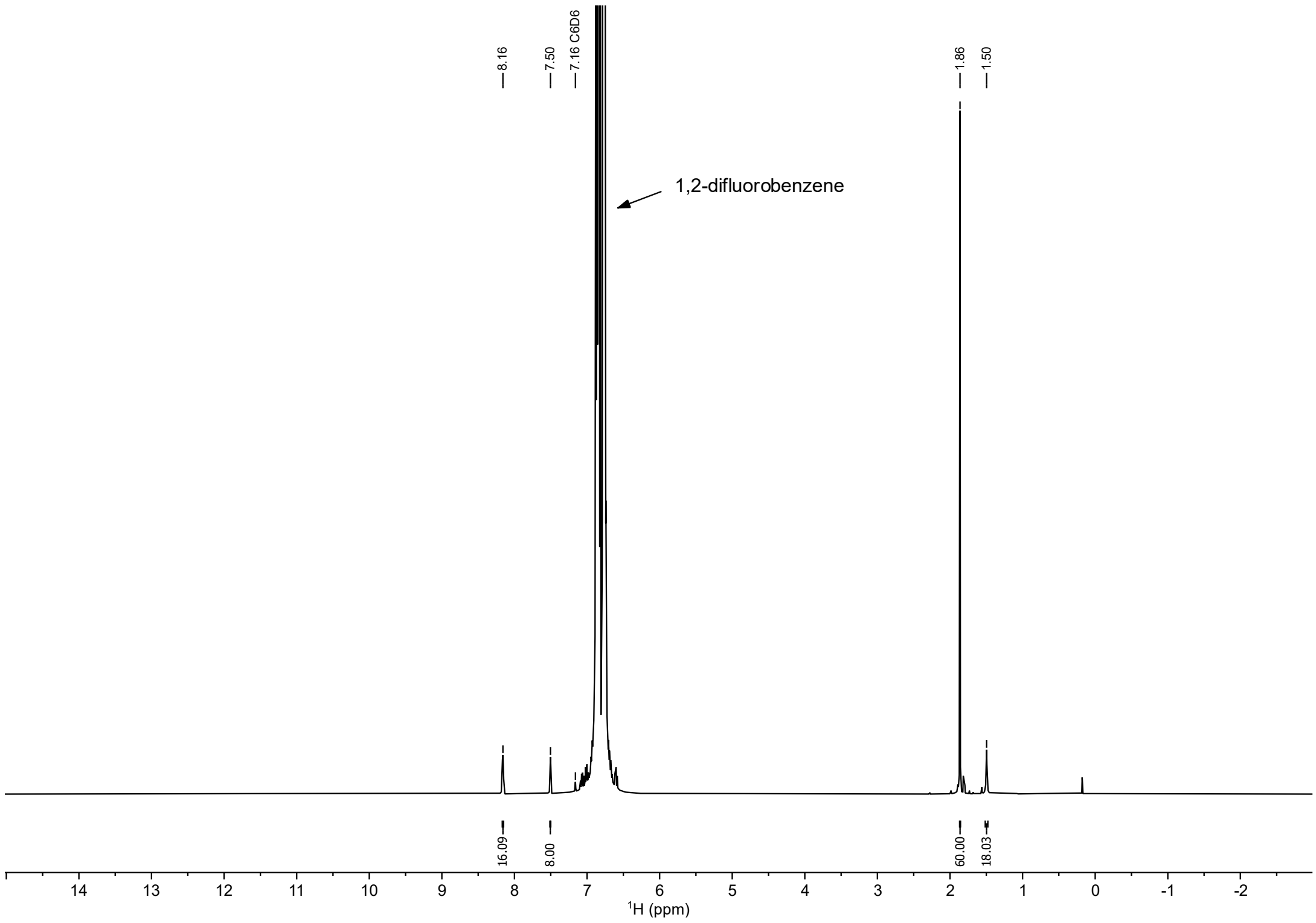

Figure S11. ^{1}H NMR spectrum of **1** in 1,2-difluorobenzene/10% C_6D_6, showing the signals of the complex at 1.86 (Cp*) and 1.50 ppm (NC^{t}Bu) as well as the two BAr^F anions at 8.16 and 7.50 ppm.

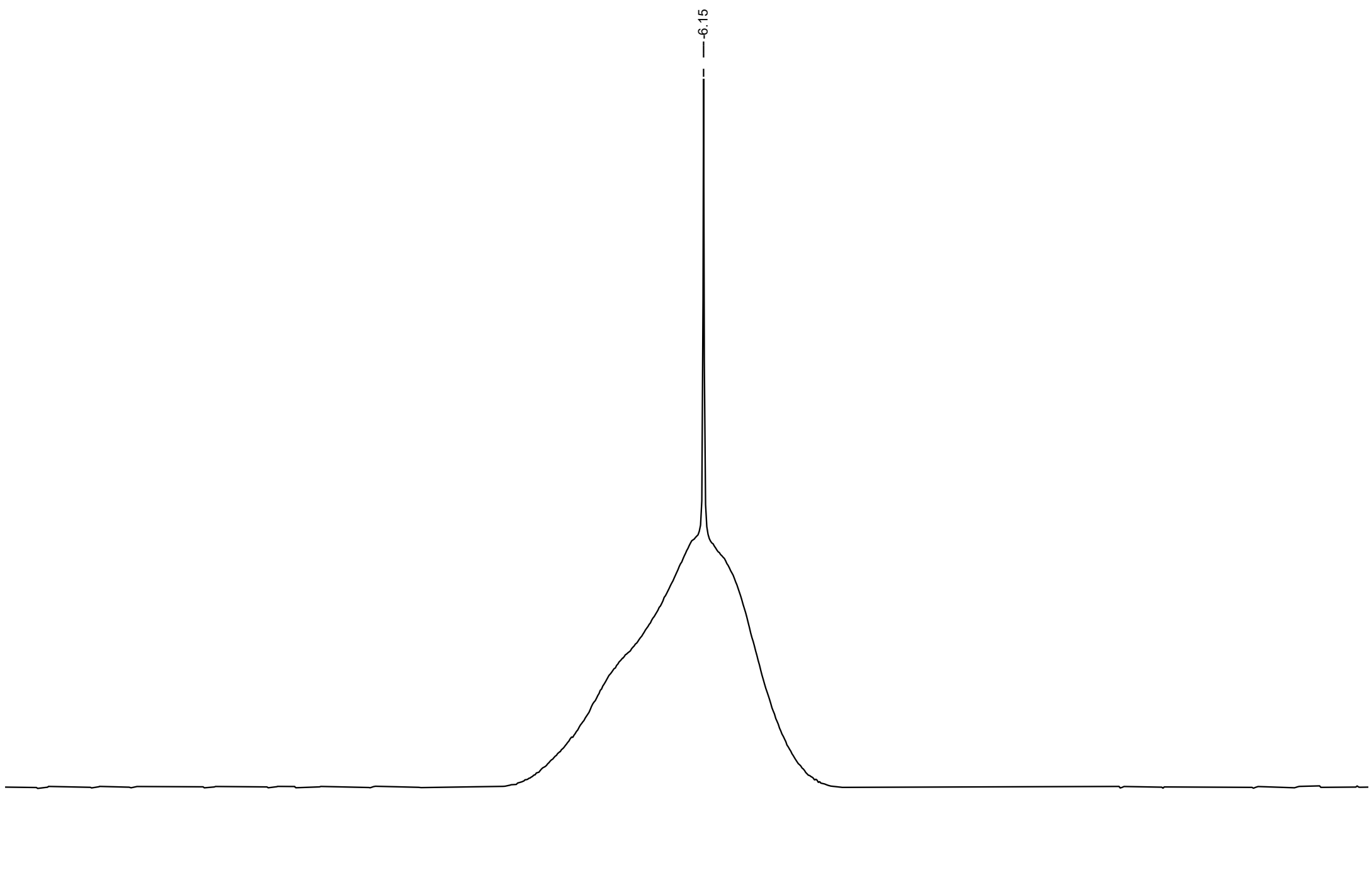

Figure S12. ^{11}B NMR spectrum of **1** in 1,2-difluorobenzene/10% C_6D_6. The peak at –6.15 ppm can be attributed to the tetraarylborate anion BAr^{F24}.

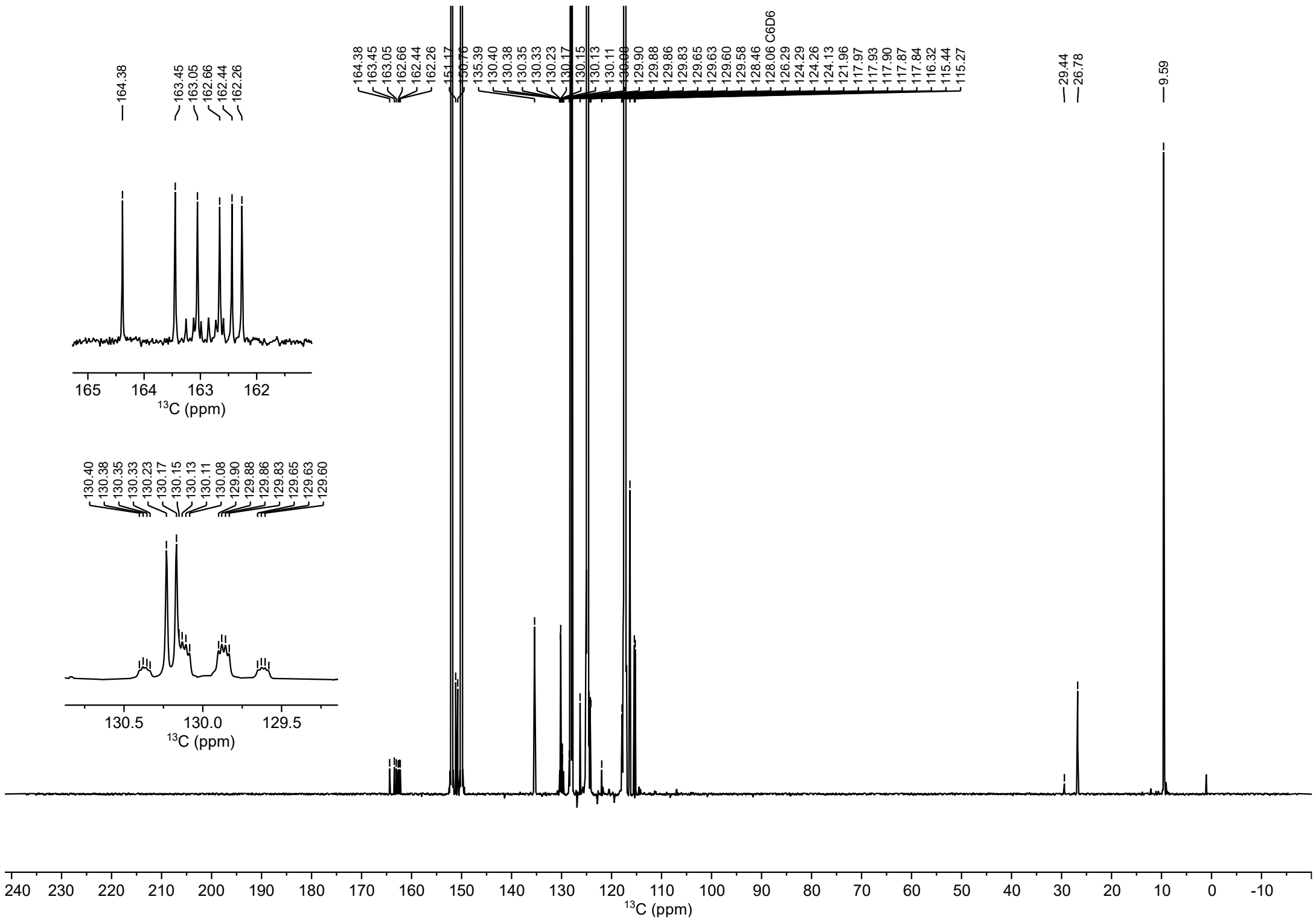

Figure S13. ^{13}C NMR spectrum of **1** in 1,2-difluorobenzene/10% C_6D_6. Note that the spectrum shows the ^{13}C NMR peaks of 1,2-difluorobenzene as the peaks of highest intensity.

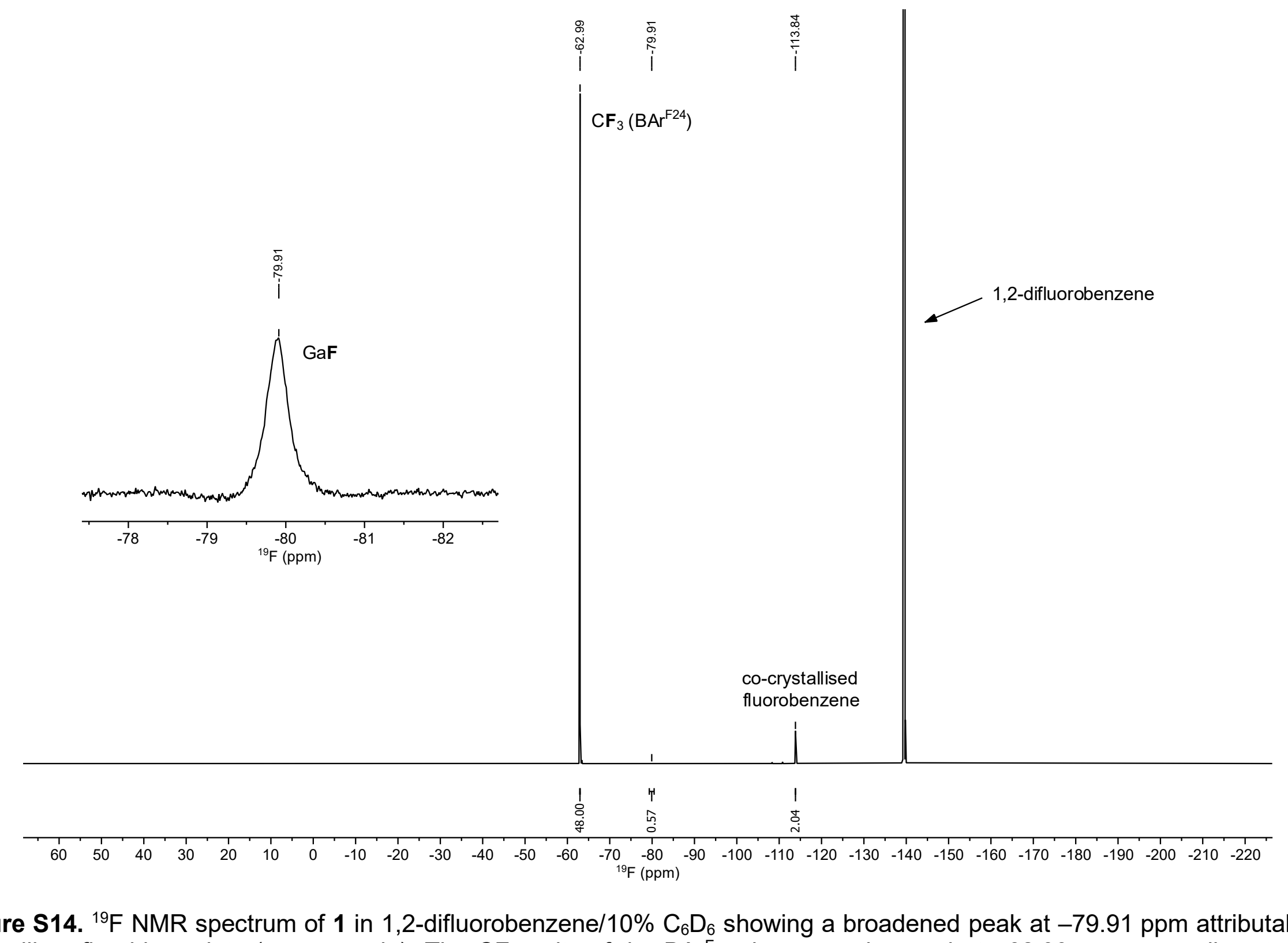

Figure S14. ^{19}F NMR spectrum of **1** in 1,2-difluorobenzene/10% C_6D_6 showing a broadened peak at –79.91 ppm attributable to the gallium fluoride moiety (see zoom-in). The CF_3 units of the BAr^F anions are observed at –62.99 ppm, as well as two co-crystallised fluorobenzene molecules at –113.84 ppm.

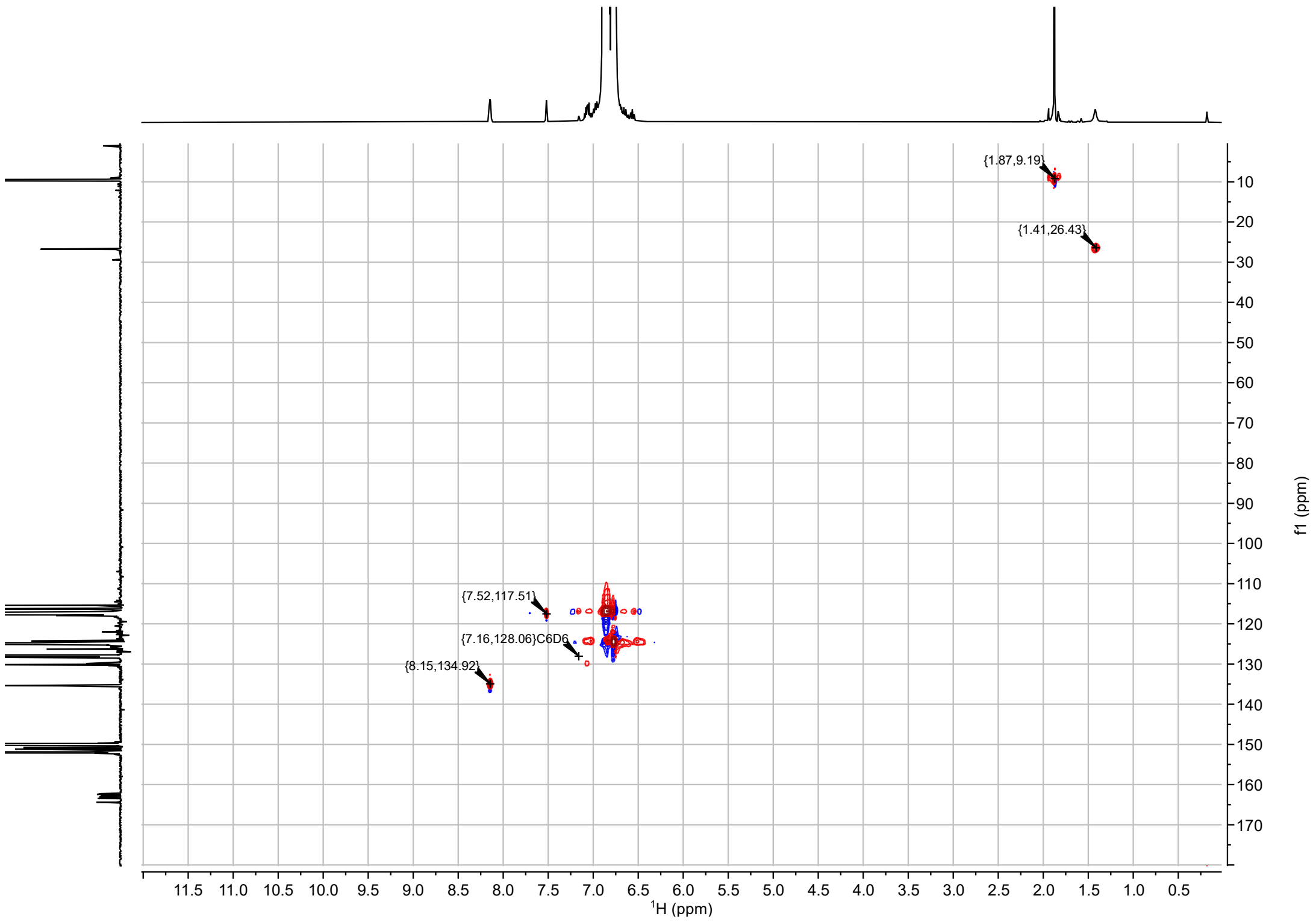

Figure S15. HSQC NMR spectrum of **1** in 1,2-difluorobenzene/10% C_6D_6. The spectrum mainly shows correlation of the BAr^{F24} anion in the aromatics region as well as Cp* and trimethylacetonitrile.

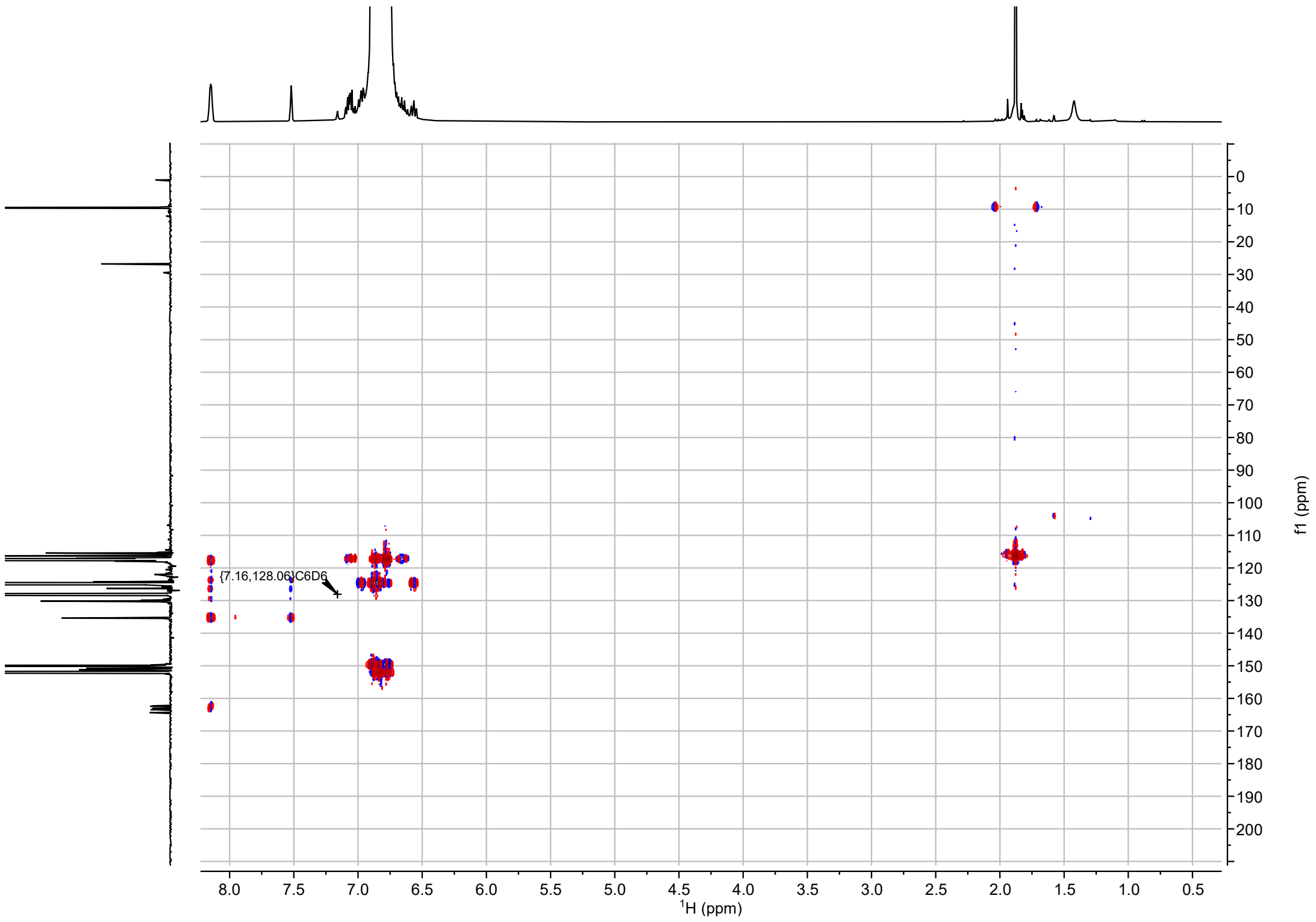

Figure S16. HMBC NMR spectrum of **1** in 1,2-difluorobenzene/10% C_6D_6. The spectrum mainly shows correlation of the BAr^{F24} anion in the aromatics region, as well as Cp* and trimethylacetonitrile.

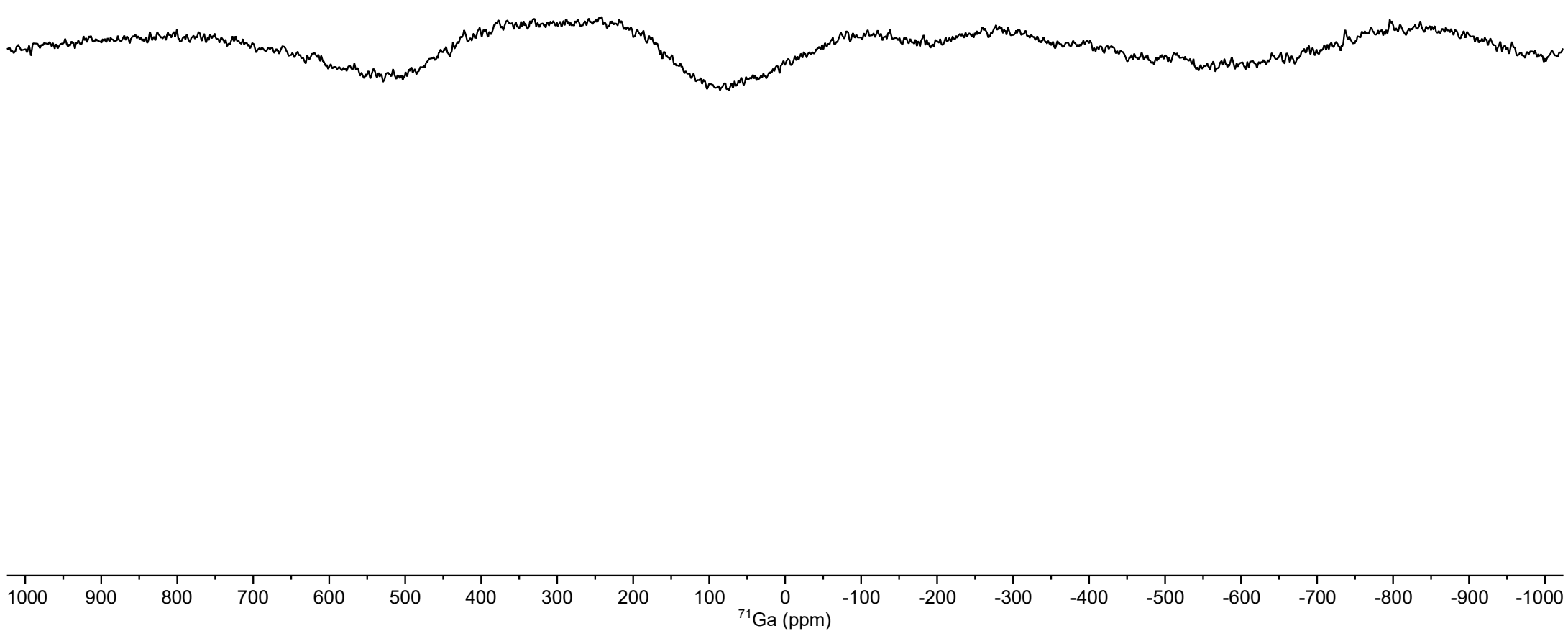

Figure S17. ^{71}Ga NMR spectrum of **1** in 1,2-difluorobenzene/10% C_6D_6 recorded at 295 K. The spectrum shows no distinct peaks, most probably due to fast exchange or Berry pseudorotation.

Compound (2)

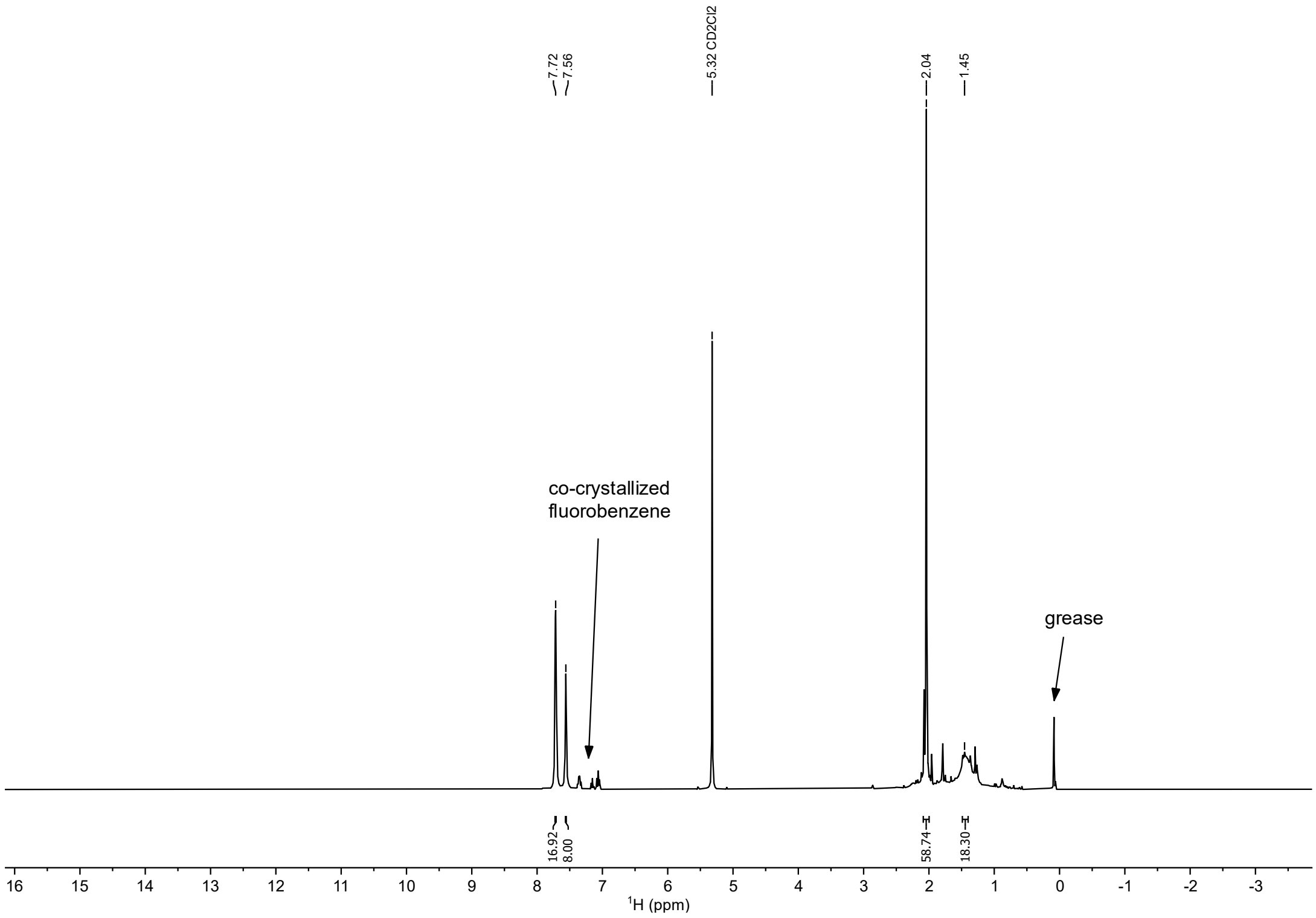

Figure S18. ^{1}H NMR spectrum of **2** in CD_2Cl_2. The singlet at 2.04 ppm (Cp* protons), a broad singlet at 1.45 ppm (NC^{t}Bu) and peaks can be assigned to two BAr^{F24} anions as well as traces of co-crystallised fluorobenzene (see crystal structure). Due to the instability of **2** in solution, slight decomposition is observed.

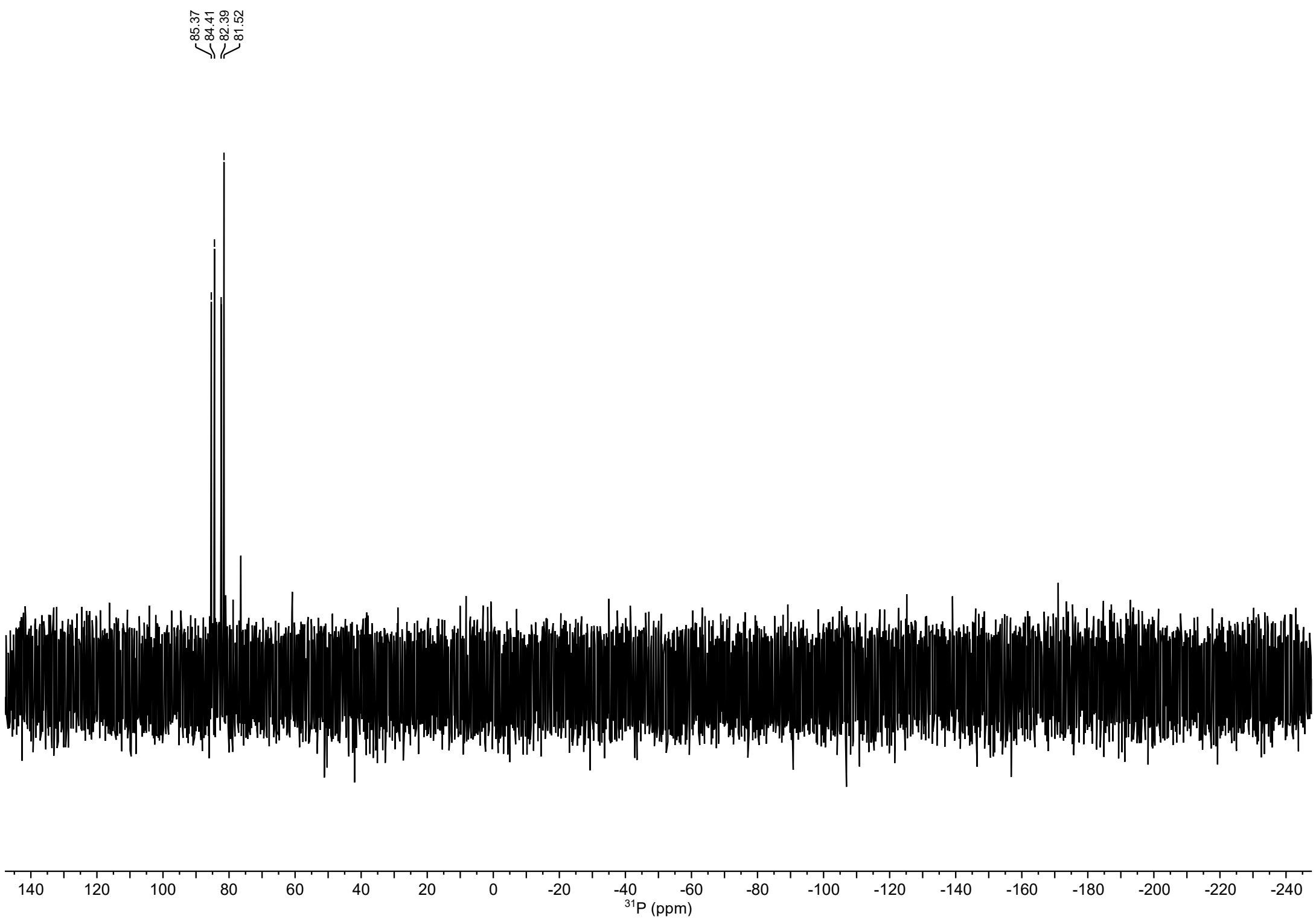

Figure S19. ^{31}P NMR spectrum of a solution of **2** (10 mg, 1.0 eq.) in 1,2-difluorobenzene at 298 K in the presence of substoichiometric amounts of triethylphosphine oxide (0.5 mg, 0.8 eq.). Due to thermal decomposition of **2** in solution, four peaks are observed in the range between 86 ppm and 81 ppm.

[Ni(NC^{t}Bu)$_6$](BAr^{F24})$_2$ & [Ni(NC^{t}Bu)$_6$](BAr^{F20})$_2$

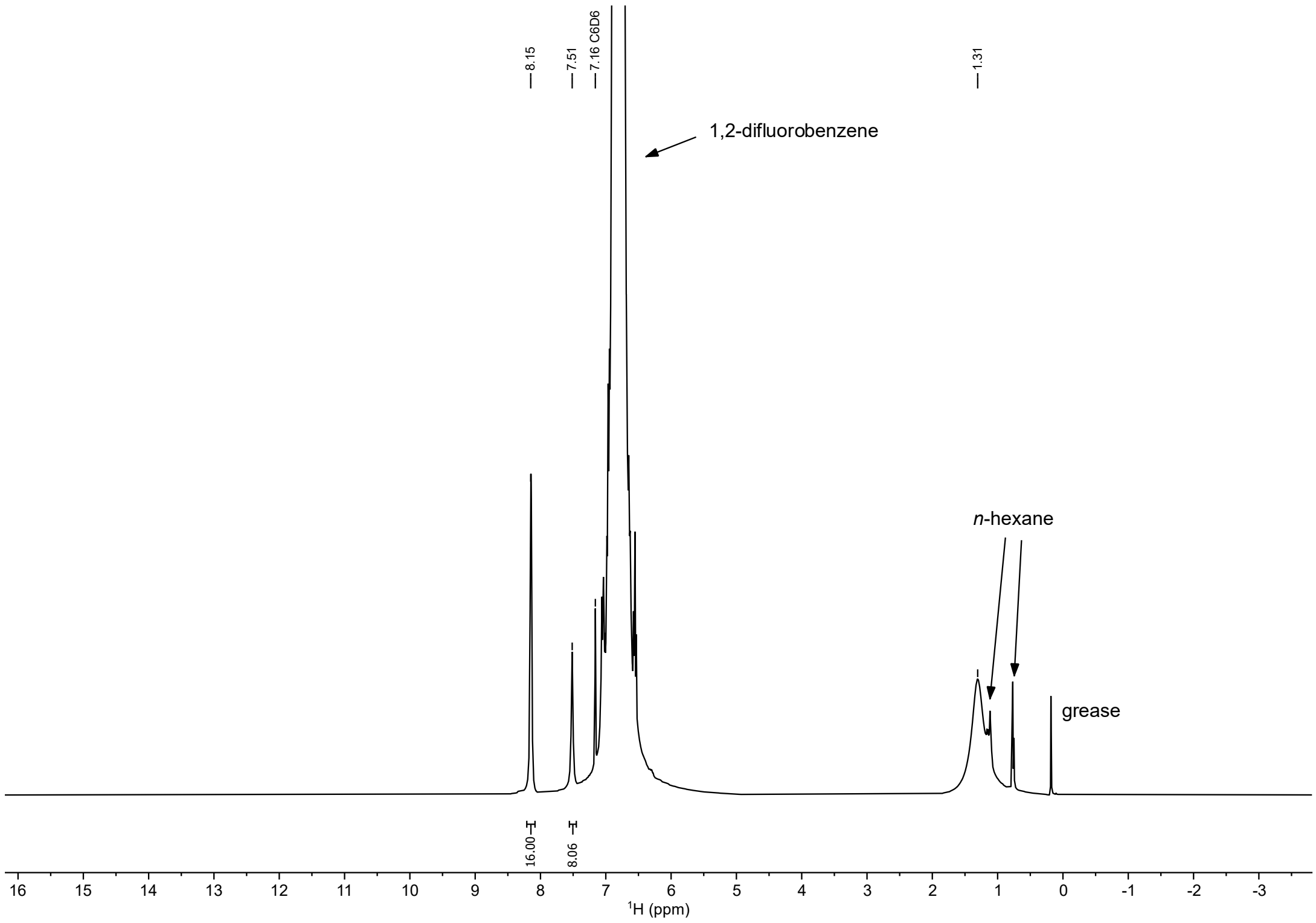

Figure S20. ^{1}H NMR spectrum of [Ni(NC^{t}Bu)$_6$](BAr^{F24})$_2$ in 1,2-difluorobenzene solution, including 10% C_6D_6. Due to the paramagnetic nature of the complex ion, the methyl groups of the nitrile ligands produce a broad signal at 1.31 ppm.

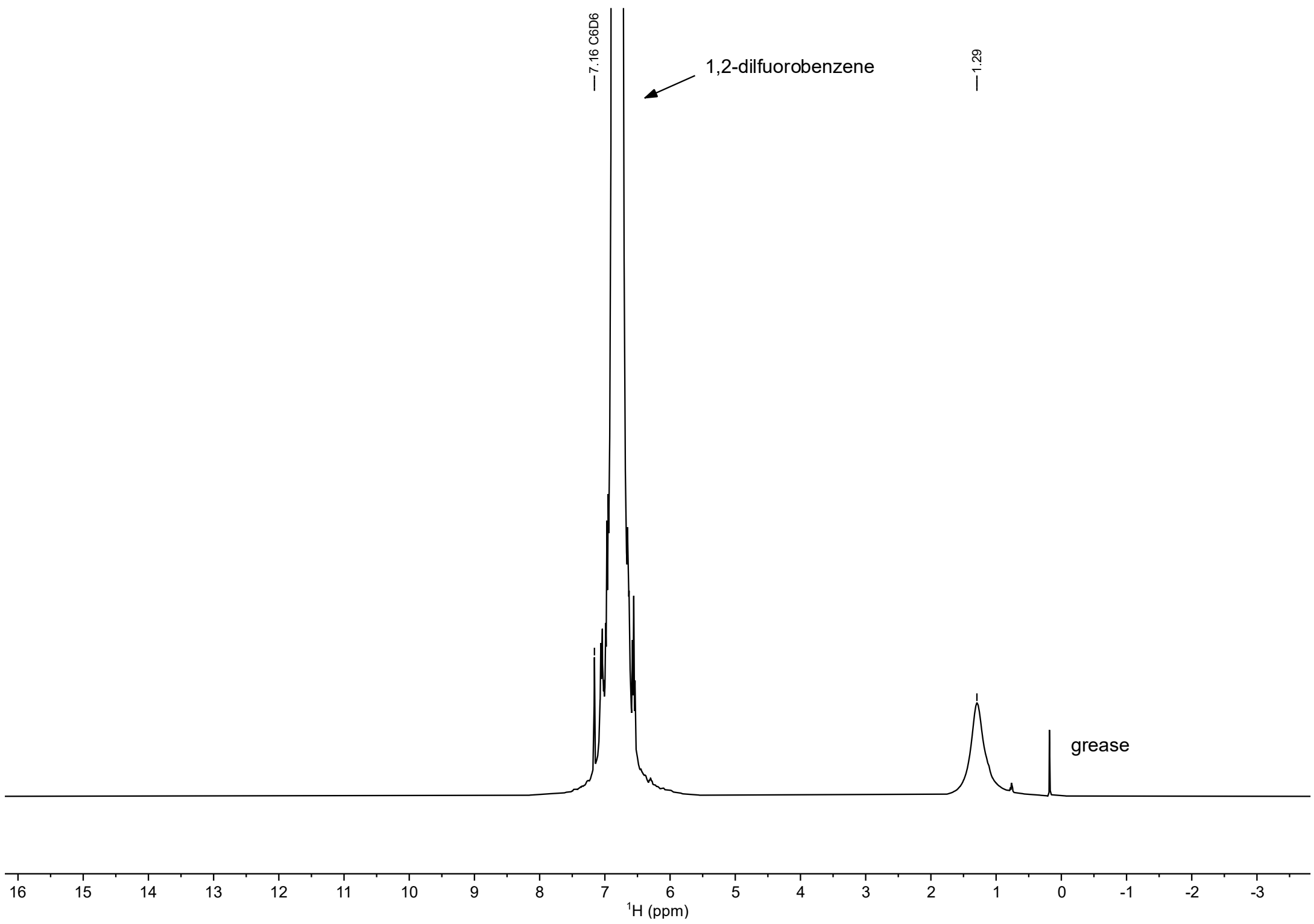

Figure S21. ^{1}H NMR spectrum of [Ni(NC^{t}Bu)$_6$](BAr^{F20})$_2$ in 1,2-difluorobenzene solution, including 10% C_6D_6. Due to the paramagnetic nature of the complex ion, the methyl groups of the nitrile ligands produce a broad signal at 1.29 ppm.

Mechanistic Aspects

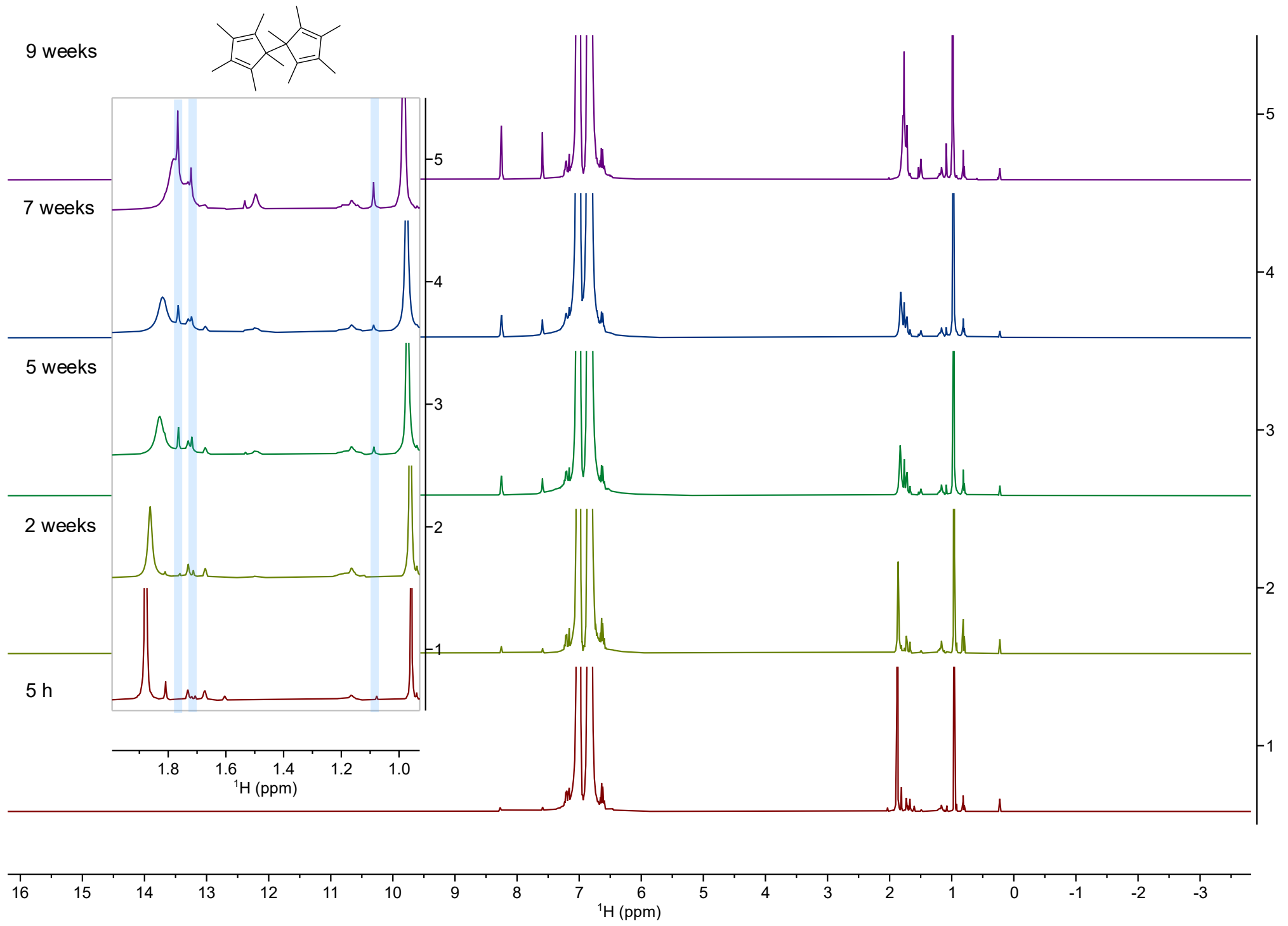

Figure S22. Stacked ^{1}H NMR spectra of the supernatant fluorobenzene solution, recorded over nine weeks until crystallisation of **1** was completed. The zoom-in to the region between 2 ppm and 1 ppm shows signals attributable to decamethyldihydrofulvalene (highlighted blue) as a characteristic decomposition product of Cp* containing species, usually due to a radical coupling mechanism.

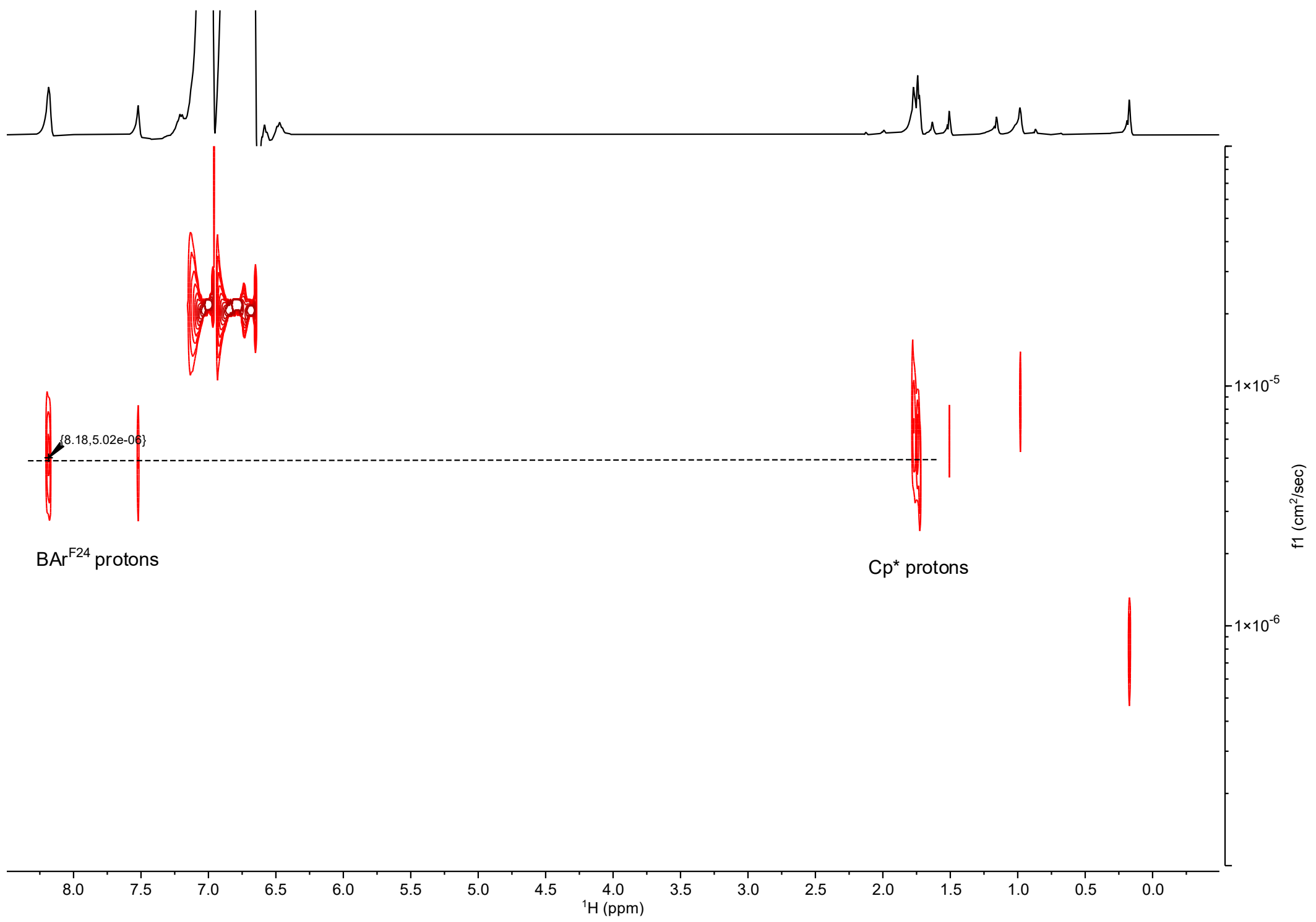

Figure S23. ^{1}H DOSY NMR spectrum of intermediate **2** in a 1:1 mixture of fluorobenzene and 1,2-difluorobenzene (for better solubility) and 10 wt% C_6D_6. The spectrum shows an ion pair for the dicationic part of **2** as deduced from the same diffusion coefficient of both the BAr^{F24} anions and the Cp* protons. Due to the thermal instability of **2** in solution, decomposition products are also observed.

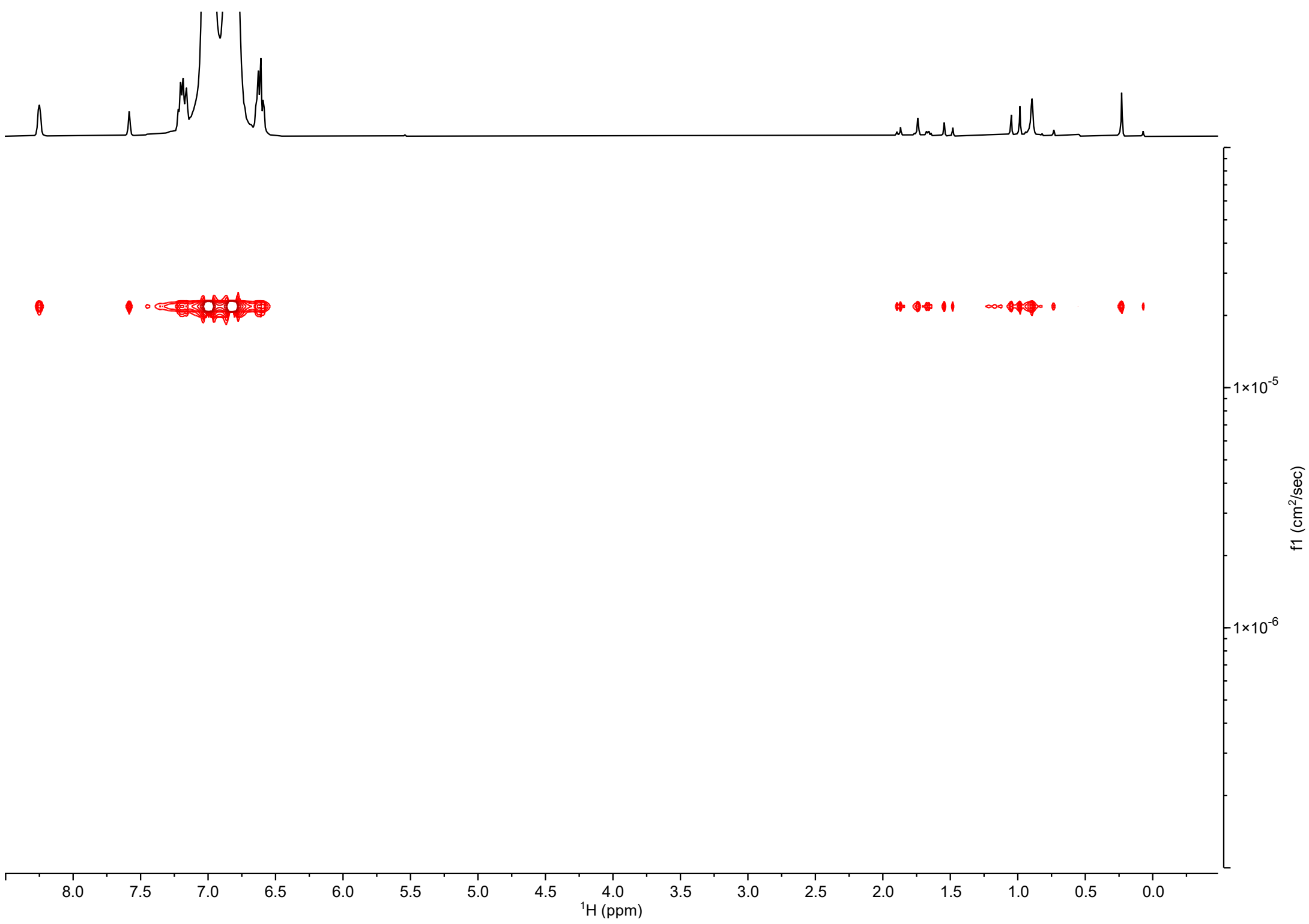

Figure S24. ^{1}H DOSY NMR spectrum of intermediate **2** in fluorobenzene suspension with 10 wt% C_6D_6. The spectrum shows no distinct signals of **2** due to its poor solubility in fluorobenzene.

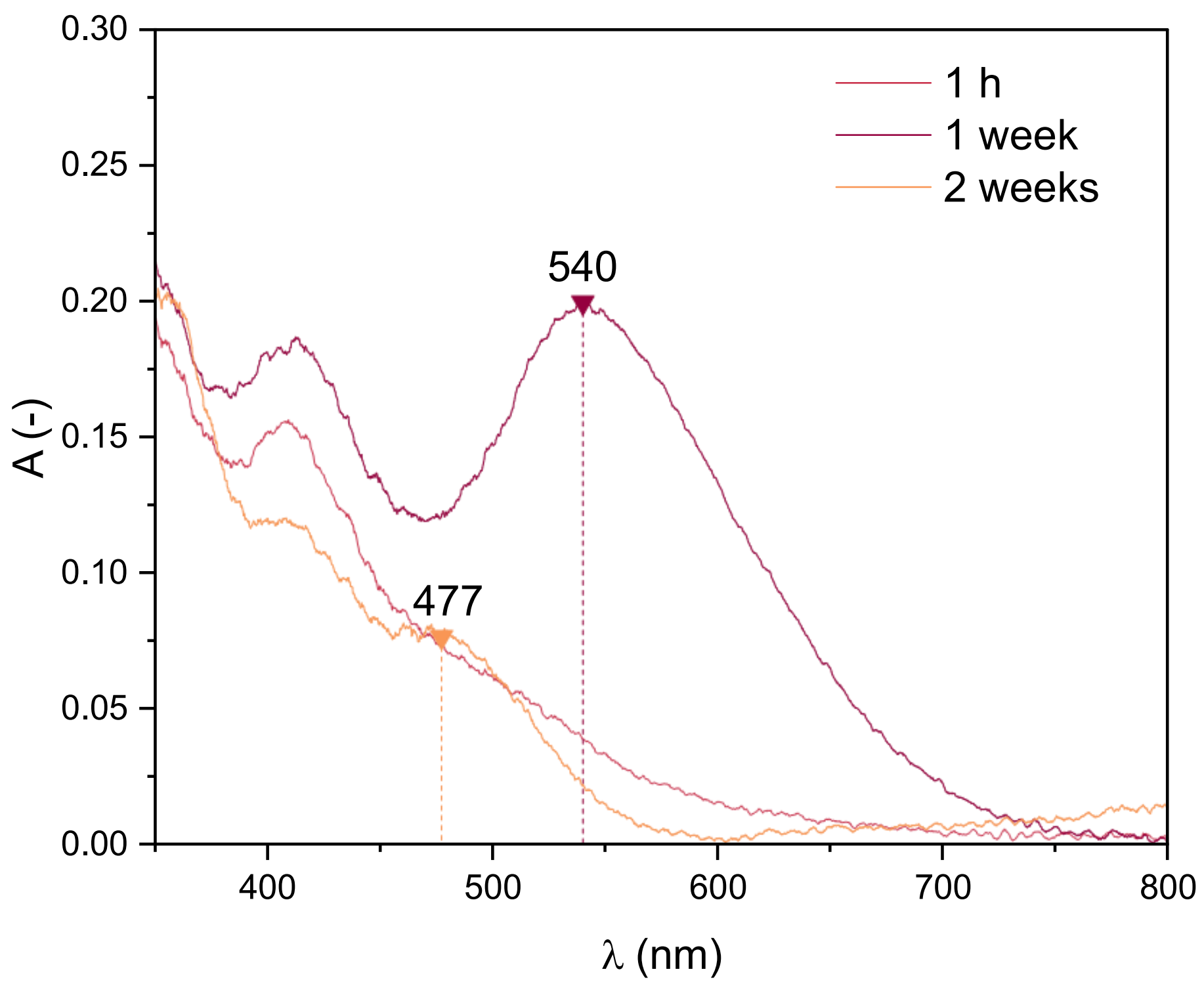

Figure S25. *In situ* UV-Vis spectra acquired with a submersible sensor from the supernatant reaction solution of **1**. The spectrum shows the formation of **2** (540 nm), its subsequent precipitation and formation of **1** in fluorobenzene solution, as identified by its characteristic band at 477 nm.

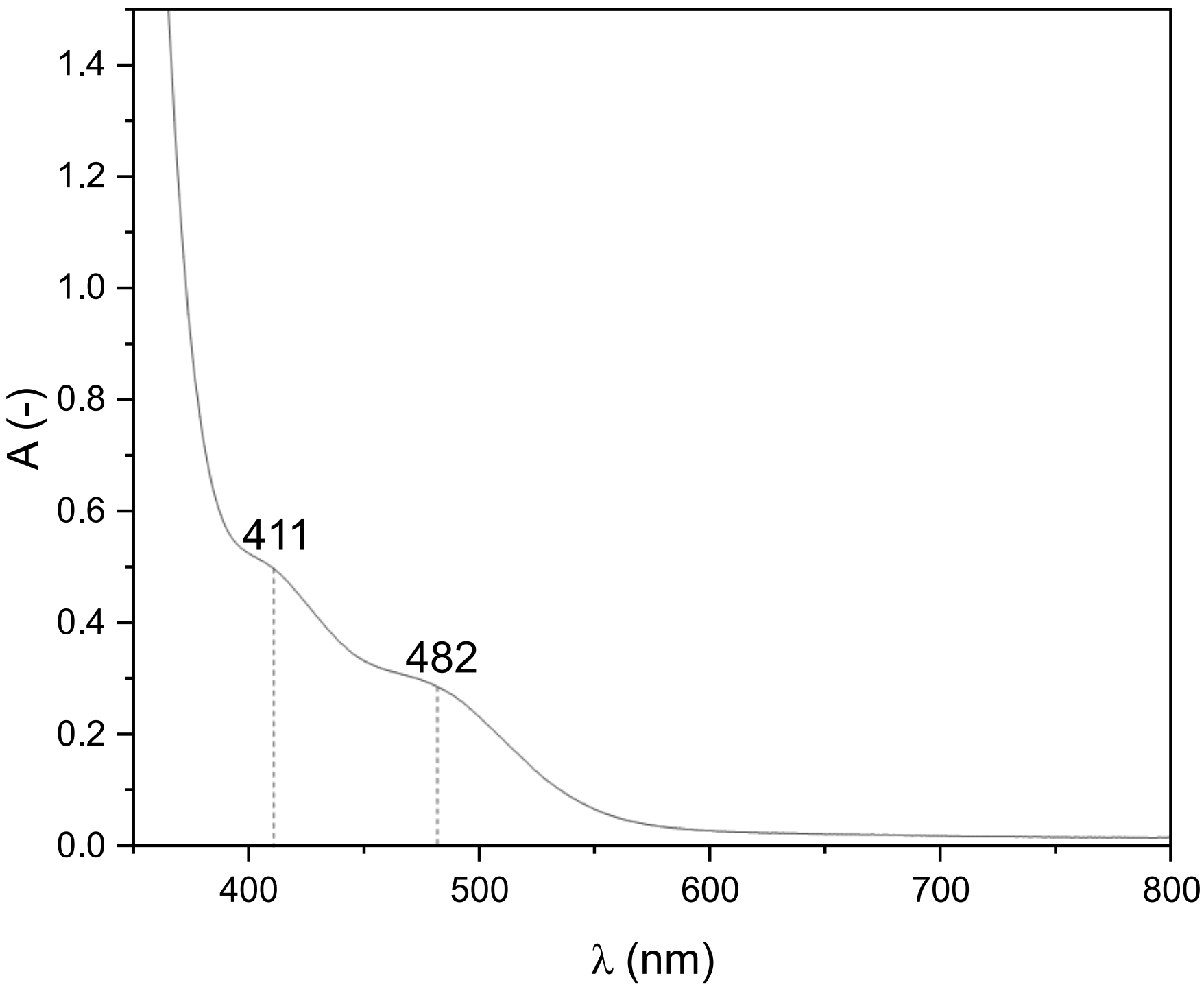

Figure S26. UV-Vis spectrum of **1** recorded in 1,2-difluorobenzene solution. The spectrum shows two characteristic shoulders at 411 and 482 nm.

Fluoride Transfer Experiments

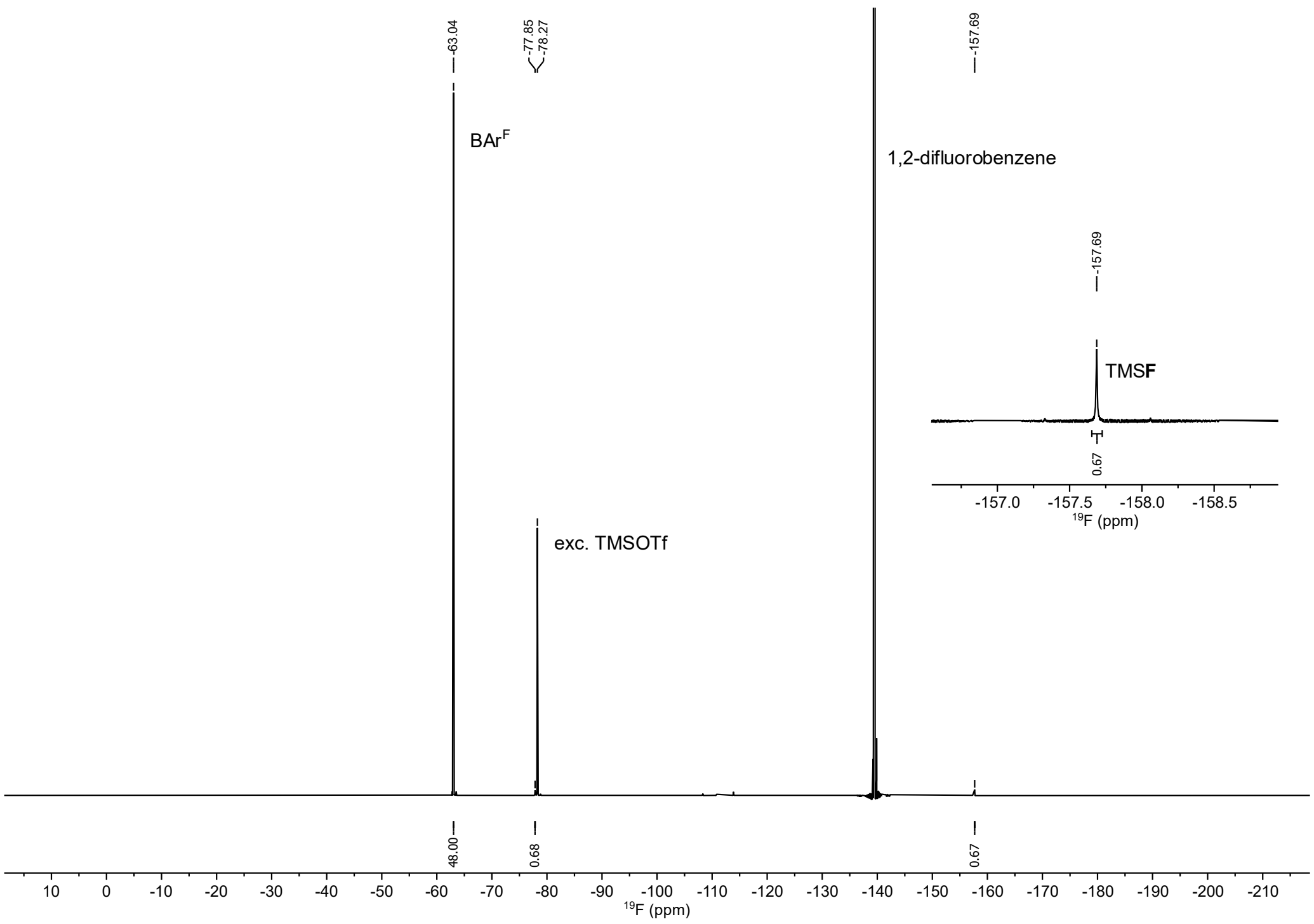

Figure S27. *In situ* ^{19}F NMR spectrum of the reaction solution of **1** in 1,2-difluorobenzene/10 w% C_6D_6 with an excess of TMSOTf. The spectrum was recorded after 15 minutes and shows the formation of TMSF as a characteristic product of fluoride transfer.

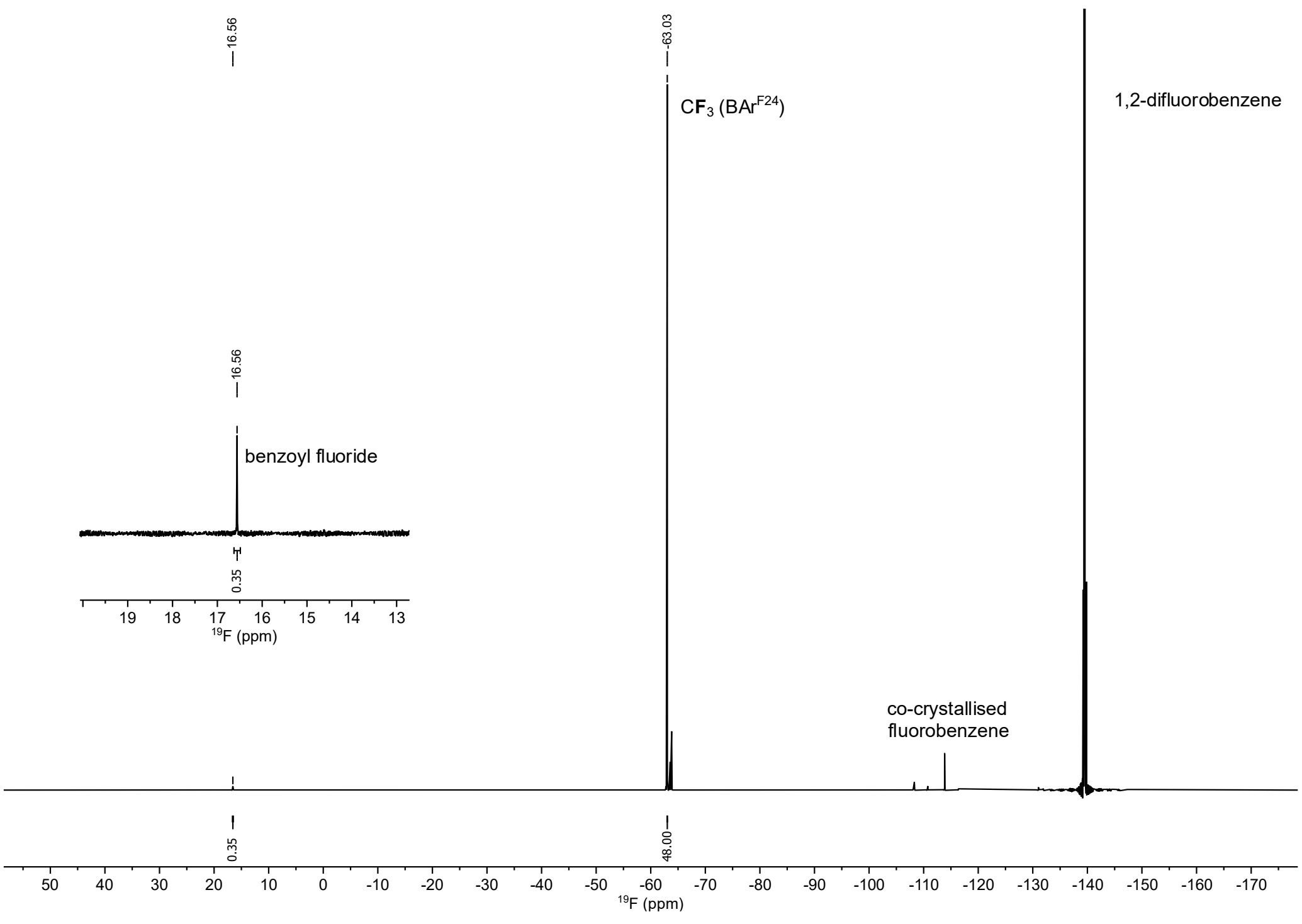

Figure S28. *In situ* ^{19}F NMR spectrum of the reaction solution of **1** in 1,2-difluorobenzene/10 w% C_6D_6 with an excess of benzoyl chloride. The spectrum was recorded after 15 minutes and shows benzoyl fluoride as a characteristic product of fluoride transfer.

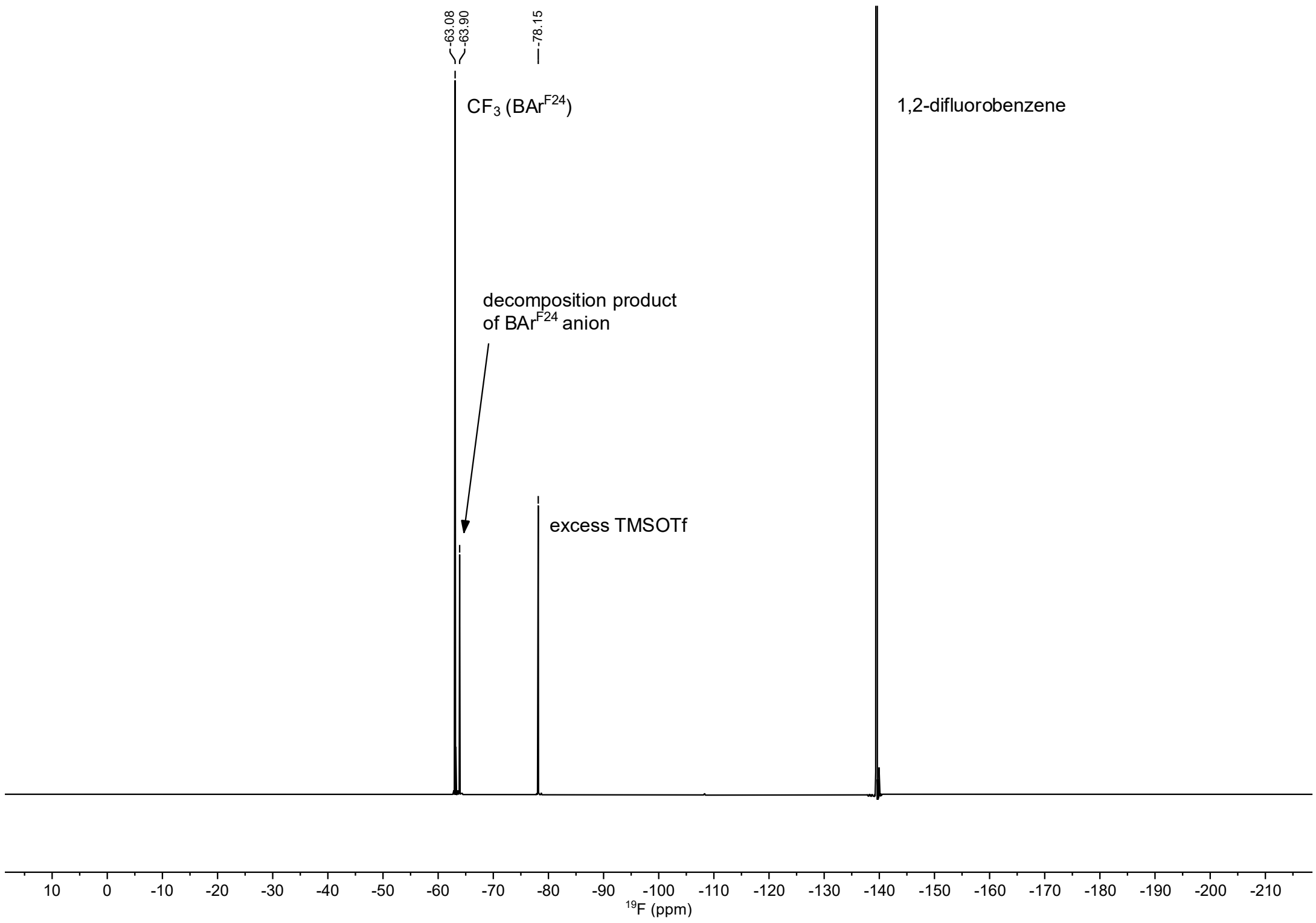

Figure S29. ^{19}F NMR spectrum of the control experiment in 1,2-difluorobenzene/10 w% C_6D_6, using $NaBAr^{F24}$ and excess TMSOTf. The spectrum shows slight decomposition of the BAr^{F24} anion but no signs of TMSF as a product of fluoride transfer.

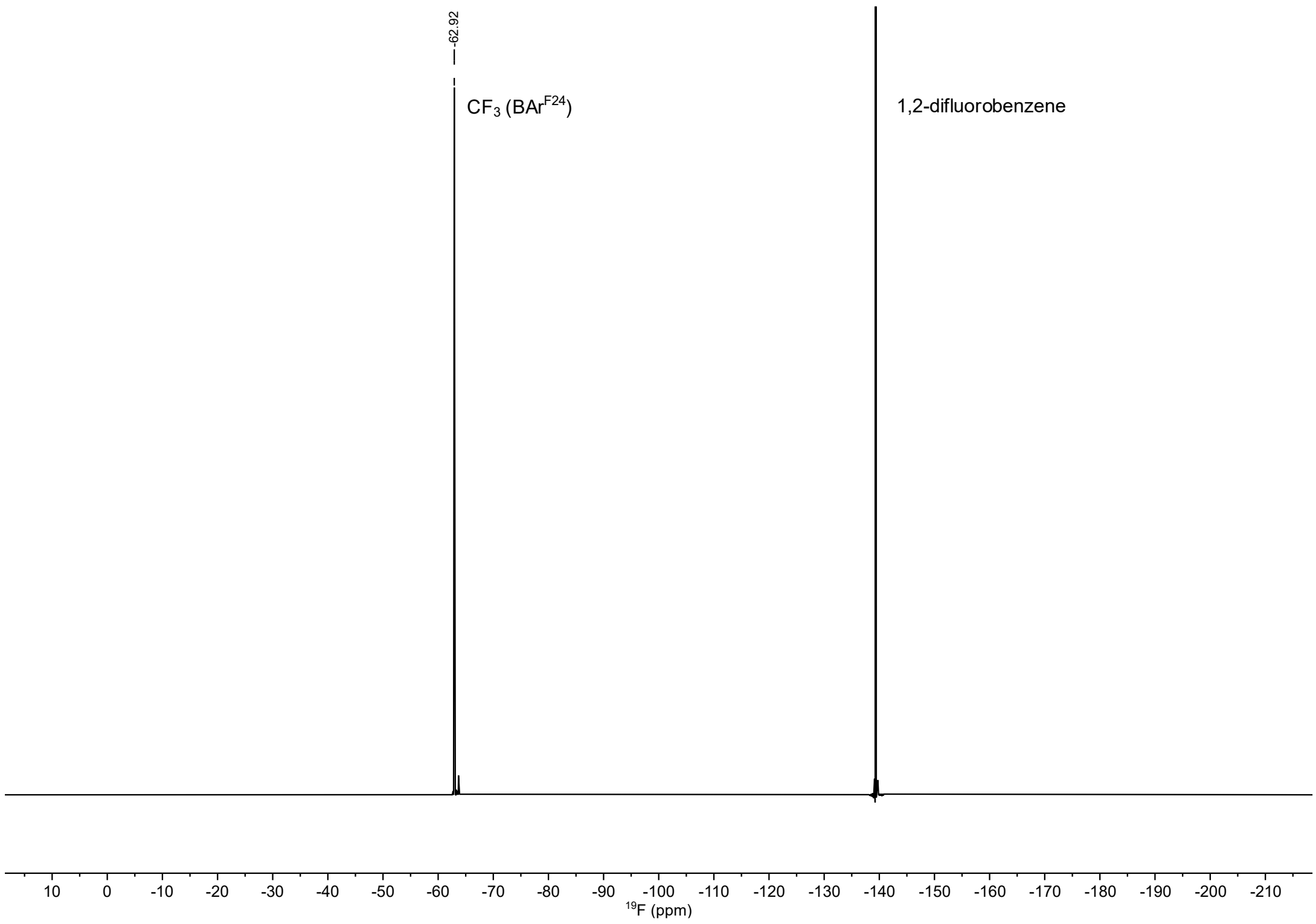

Figure S30. ^{19}F NMR spectrum of the control experiment in 1,2-difluorobenzene/10 w% C_6D_6, using $NaBAr^{F24}$ and excess benzoyl chloride. The spectrum shows no signs of benzoyl fluoride as a product of fluoride transfer.

7. IR Spectra

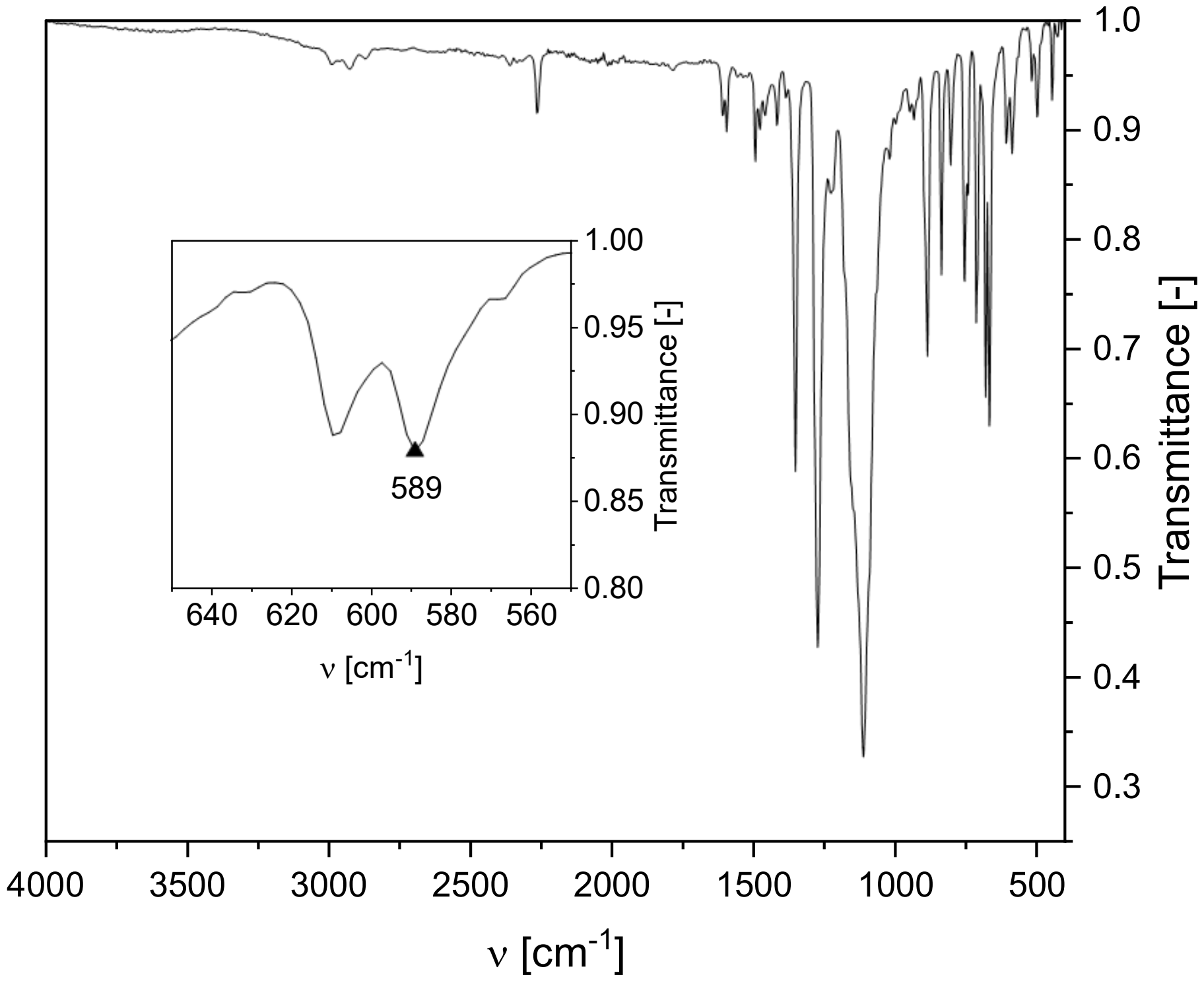

Figure S31. ATR-IR spectrum of [(Cp*Ga)$_4$Ni(GaF(NC^{t}Bu)$_2$)](BAr^{F24})$_2$ (**1**) in the solid state. It shows characteristic bands for coordinated trimethylacetonitrile at 2266 cm^{-1} as well as intensive bands attributable to C-F bonds of the BAr^{F24} anions (1354, 1275 and 1114 cm^{-1}). The Ga–F vibrational band is located at 589 cm^{-}

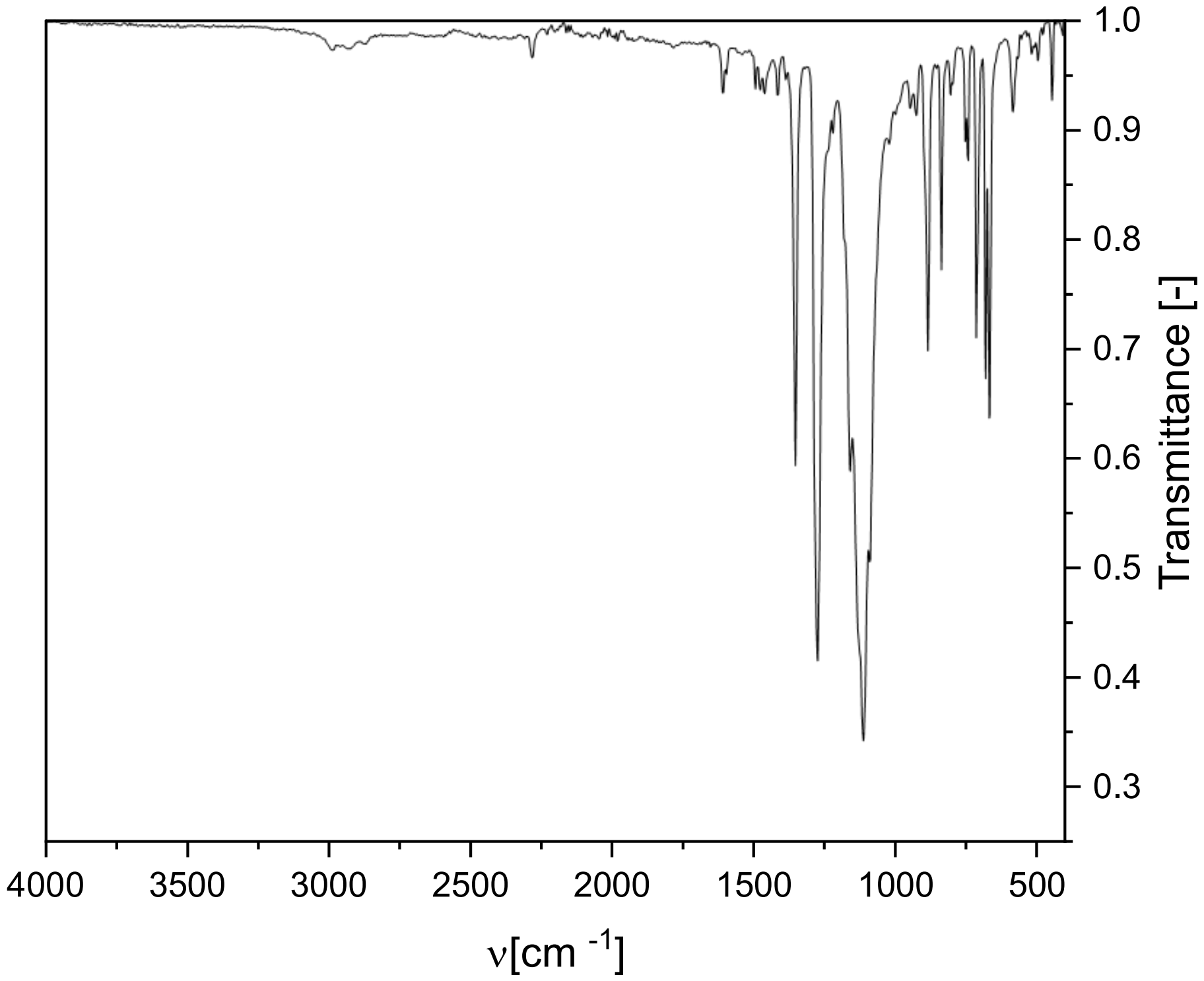

Figure S32. ATR-IR spectrum of [(Cp*Ga)$_4$Ni(NC^{t}Bu)](BAr^{F24})$_2$ (**2**) in the solid state. It shows characteristic bands for coordinated trimethylacetonitrile at 2283 cm^{-1} as well as intensive bands attributable to C-F bonds of the BAr^{F24} anions (1416, 1276 and 1114 cm^{-1}).

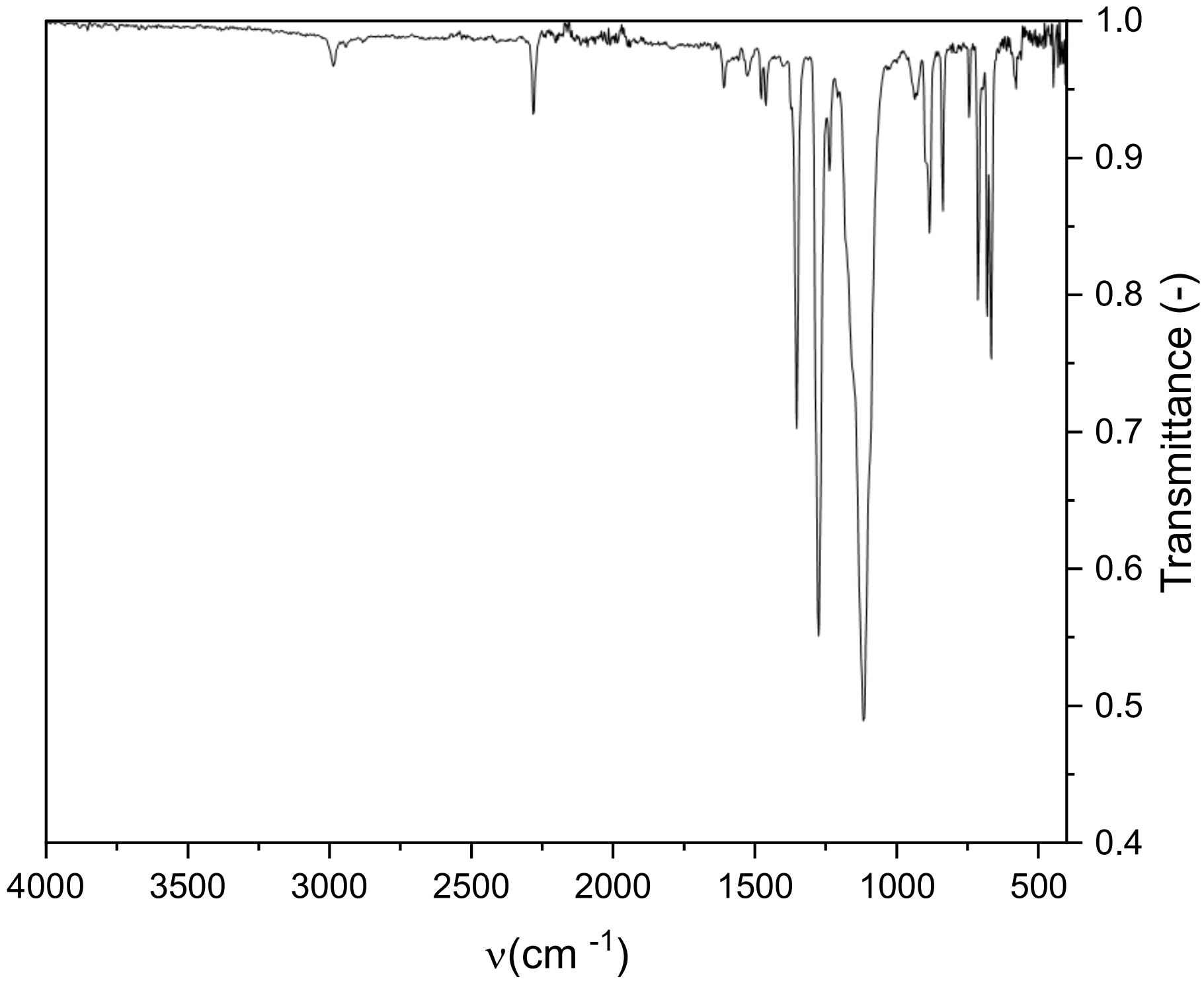

Figure S33. ATR-IR spectrum of [Ni(NC^{t}Bu)$_6$](BAr^{F24})$_2$ in the solid state. The spectrum shows characteristic bands for coordinated trimethylacetonitrile at 2282 cm^{-1} as well as intensive bands attributable to C-F bonds of the BAr^{F24} anions (1354, 1275 and 1114 cm^{-1}).

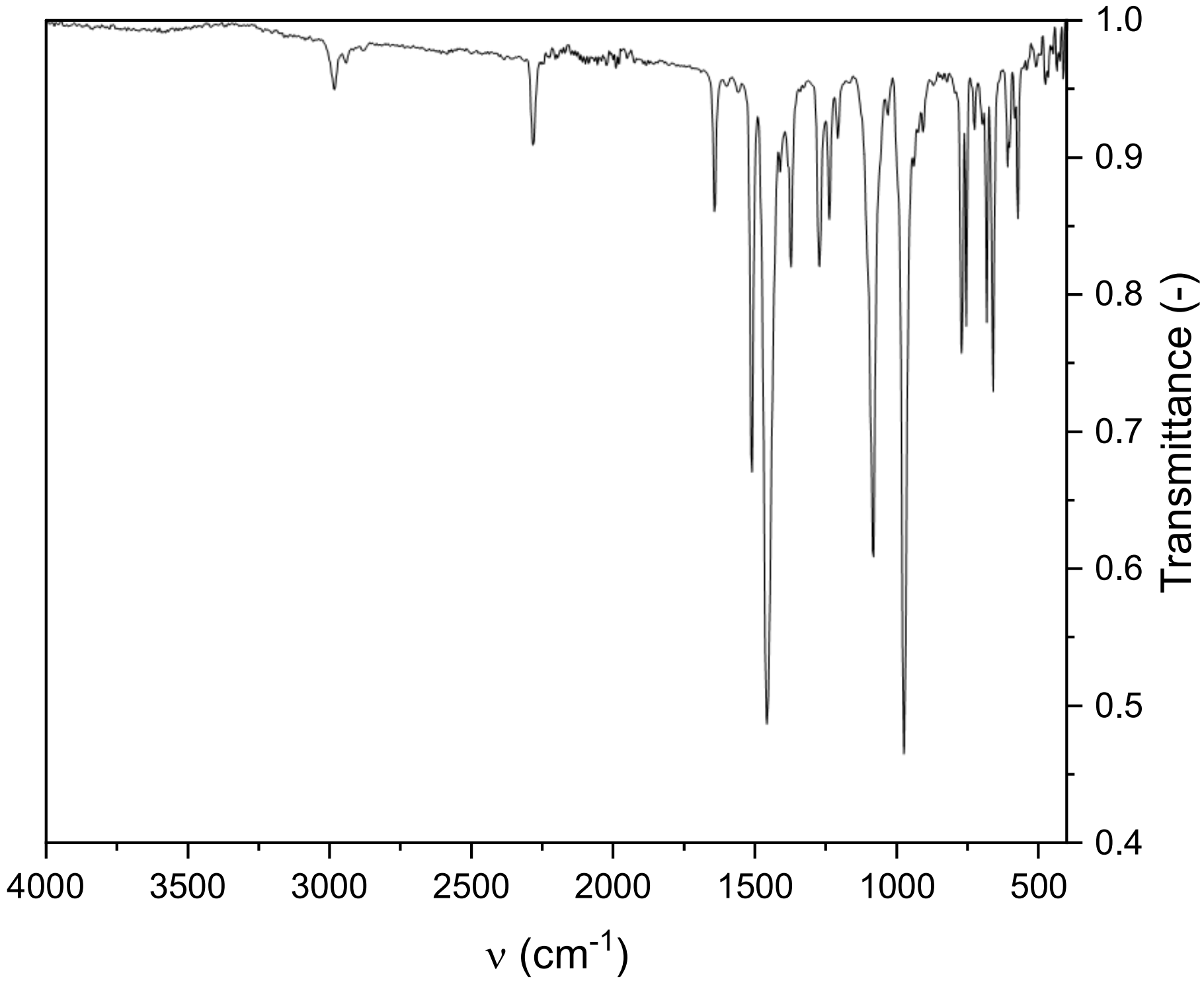

Figure S34. ATR-IR spectrum of [Ni(NC^{t}Bu)$_6$](BAr^{F20})$_2$ in the solid state. The spectrum shows characteristic bands for coordinated trimethylacetonitrile at 2284 cm^{-1} as well as intensive bands attributable to C-F bonds of the BAr^{F24} anions (1459, 1084 and 975 cm^{-1}).

8. References

[1] W. L. F. Armarego, D. D. Perrin, *Purification of Laboratory Chemicals*, Elsevier Science & Technology Books, **1997**.
[2] P. Jutzi, L. O. Schebaum, *J. Organomet. Chem.* **2002**, *654*, 176-179.
[3] J. Stephan, R. Bühler, F. E. Napoli, C. Gemel, R. A. Fischer, *Inorg. Chem.* **2025**, *64*, 15707-15714.
[4] in *Inorganic Syntheses*, **2002**, pp. 75-121.
[5] N. A. Yakelis, R. G. Bergman, *Organometallics* **2005**, *24*, 3579-3581.
[6] C. R. Smith, Aibin Zhang, D. J. Mans, T. V., RajanBabu, *Org. Synth.* **2008**, *85*, 248-266.
[7] M. Brookhart, B. Grant, A. F. Volpe, Jr., *Organometallics* **1992**, *11*, 3920-3922.
[8] L. Omann, M. Oestreich, *Organometallics* **2017**, *36*, 767-776.
[9] D. Roth, J. Stirn, D. W. Stephan, L. Greb, *J. Am. Chem. Soc.* **2021**, *143*, 15845-15851.
[10] M. Olschewski, R. Gustus, M. Marschewski, O. Höfft, F. Endres, *Phys. Chem. Chem. Phys.* **2014**, *16*, 25969-25977.
[11] S. Massicot, T. Sasaki, M. Lexow, S. Shin, F. Maier, S. Kuwabata, H.-P. Steinrück, *Langmuir* **2021**, *37*, 11552-11560.
[12] G. te Velde, F. M. Bickelhaupt, E. J. Baerends, C. Fonseca Guerra, S. J. A. van Gisbergen, J. G. Snijders, T. Ziegler, *J. Comput. Chem.* **2001**, *22*, 931-967.
[13] E. van Lenthe, E. J. Baerends, J. G. Snijders, *J. Chem. Phys.* **1993**, *99*, 4597-4610.
[14] E. van Lenthe, E. J. Baerends, J. G. Snijders, *J. Chem. Phys.* **1994**, *101*, 9783-9792.
[15] E. van Lenthe, E. J. Baerends, *J. Comput. Chem.* **2003**, *24*, 1142-1156.
[16] C. Adamo, V. Barone, *J. Chem. Phys.* **1999**, *110*, 6158-6170.
[17] J. P. Perdew, K. Burke, M. Ernzerhof, *Phys. Rev. Lett.* **1996**, *77*, 3865-3868.
[18] J. P. Perdew, K. Burke, M. Ernzerhof, *Phys. Rev. Lett.* **1997**, *78*, 1396.
[19] S. Grimme, *J. Comput. Chem.* **2006**, *27*, 1787-1799.
[20] E. D. Glendening, J. K. Badenhoop, A. E. Reed, J. E. Carpenter, J. A. Bohmann, C. M. Morales, F. Weinhold, NBO 6.0, University of Wisconsin (Madison, WI, 2001, http://nbo6.chem.wisc.edu).
[21] R. F. W. Bader, *Atoms in Molecules-A Quantum Theory*, Oxford University Press, Oxford, England, **1990**.
[22] J. I. Rodríguez, *J. Comput. Chem.* **2013**, *34*, 681-686.
[23] J. I. Rodríguez, R. F. W. Bader, P. W. Ayers, C. Michel, A. W. Götz, C. Bo, *Chem. Phys. Lett.* **2009**, *472*, 149-152.
[24] F. M. Bickelhaupt, E. J. Baerends, *Reviews in Computational Chemistry*, Wiley, New York **2000**.
[25] K. Morokuma, *J. Chem. Phys.* **1971**, *55*, 1236-1244.
[26] T. Ziegler, A. Rauk, *Inorg. Chem.* **1979**, *18*, 1558-1565.
[27] S. K. Wolff, T. Ziegler, E. van Lenthe, E. J. Baerends, *J. Chem. Phys.* **1999**, *110*, 7689-7698.
[28] K. Momma, F. Izumi, *J. Appl. Crystallogr.* **2011**, *44*, 1272-1276.
[29] M. Lehmann, S. N. Balogun, M. Reimann, M. Kaupp, *Chem. Eur. J.* **2025**, *31*, e202404662.
[30] T. Yanai, D. P. Tew, N. C. Handy, *Chem. Phys. Lett.* **2004**, *393*, 51-57.
[31] A. K. Hijazi, A. Al Hmaideen, S. Syukri, N. Radhakrishnan, E. Herdtweck, B. Voit, F. E. Kühn, *Eur. J. Inorg. Chem.* **2008**, *2008*, 2892-2898.
[32] A. M. Ajlouni, A. K. Hijazi, Z. A. Taha, W. Al-Momani, A. Okour, F. E. Kühn, *Catal. Lett.* **2019**, *149*, 2317-2324.
[33] V. Gutmann, *Coord. Chem. Rev.* **1976**, *18*, 225-255.
[34] M. A. Beckett, G. C. Strickland, J. R. Holland, K. Sukumar Varma, *Polymer* **1996**, *37*, 4629-4631.
[35] J. L. Bourque, M. C. Biesinger, K. M. Baines, *Dalton. Trans.* **2016**, *45*, 7678-7696.
[36] T. Yoshida, K. Yamasaki, *Bull. Chem. Soc. Jap.* **1981**, *54*, 935-936.
[37] M. C. Biesinger, L. W. M. Lau, A. R. Gerson, R. S. C. Smart, *Phys. Chem. Chem. Phys.* **2012**, *14*, 2434-2442.
[38] M. C. Biesinger, B. P. Payne, L. W. M. Lau, A. Gerson, R. S. C. Smart, *Surf. Interface Anal.* **2009**, *41*, 324-332.
[39] I. Antsiburov, R. Bühler, J. Stephan, M. Erdyakov, C. Gemel, S. Kahlal, O. Cador, T. Guizouarn, J.-Y. Saillard, K. Meyer, R. A. Fischer, *Chem. Sci.* **2026**, *17*, 8989-8997.

Crystallography

C1. APEX4 Suite of Crystallographic Software, Version 2021-10.0, Bruker AXS Inc., Madison, Wisconsin, USA (2021).

C2. Bruker, SAINT, V8.40B, Bruker AXS Inc., Madison, Wisconsin, USA (2021).
C3. SADABS, Version 2021, Bruker AXS Inc., Madison, Wisconsin, USA (2021).
C4. Sheldrick, G. M., Crystal structure refinement with SHELXL. *Acta Crystallogr. Sect. C* **71**, 3–8 (2015).
C5. C. B. Hübschle, G. M. Sheldrick, B. Dittrich, *J. Appl. Cryst.* **2011**, *44*, 1281–1284.
C6. *International Tables for Crystallography*, Vol. C (Ed.: A. J. Wilson), Kluwer Academic Publishers, Dordrecht, The Netherlands, 1992, Tables 6.1.1.4 (pp. 500–502), 4.2.6.8 (pp. 219–222), and 4.2.4.2 (pp. 193–199).
C7. D: Kratzer, J. J. Holstein, I. Krossing, *J. Appl. Cryst.* **2015**, *48*, 933–938.
C8. A. L. Spek, *Acta Crystallogr. Sect. C* **2015**, *71*, 9–18.
C9. C. F. Macrae, I. J. Bruno, J. A. Chisholm, P. R. Edgington, P. McCabe, E. Pidcock, L. Rodriguez-Monge, R. Taylor, J. van de Streek, P. A. Wood, *J. Appl. Crystallogr.* **2008**, *41*, 466-470.
C10. D. Kratzert, FinalCif, V150, Freiburg, Germany (2025), https://dkratzert.de/finalcif.html.
